# Anti‐Quenching NIR‐II Excitation Phenylboronic Acid Modified Conjugated Polyelectrolyte for Intracellular Peroxynitrite‐Enhanced Chemo–Photothermal Therapy

**DOI:** 10.1002/advs.202309446

**Published:** 2024-06-17

**Authors:** Pengfei Sun, Danni Hu, Pengfei Chen, Xuanzong Wang, Qingming Shen, Shangyu Chen, Daifeng Li, Quli Fan

**Affiliations:** ^1^ State Key Laboratory of Organic Electronics and Information Displays & Institute of Advanced Materials Jiangsu Key Laboratory for Biosensors Nanjing University of Posts & Telecommunications Nanjing 210023 China; ^2^ Department of Orthopedics The First Affiliated Hospital of Zhengzhou University Zhengzhou 450052 China

**Keywords:** Anti‐NIR‐II fluorescence quenching, conjugated polyelectrolyte, NIR‐II excitation, NIR‐II photothermal therapy, peroxynitrite

## Abstract

Multidrug resistance to clinical chemotherapeutic drugs severely limits antitumor efficacy and patient survival. The integration of chemotherapy with photothermal therapy (PTT) and reactive nitrogen species has become a major strategy to enhance cancer treatment efficacy. Herein, a multifunctional peroxynitrite (ONOO^−^) nanogenerator (PBT/NO/Pt) for NIR‐II fluorescence (NIR‐II FL)/NIR‐II photoacoustic (NIR‐II PA) imaging‐guided chemo/NIR‐II PTT/ONOO^−^ combination therapy is reported. The multifunction nanogenerator is developed by co‐loading a pH‐sensitive nitric oxide donor (DETA NONOate) and nicotinamide adenine dinucleotide phosphate oxidases trigger superoxide (O_2_^•−^) generator chemotherapy drug (CDDP) to an NIR‐II excitation‐conjugated polyelectrolyte (PNC11BA). PNC11BA has non‐conjugated alkyl chain segments in the polymer backbone and abundant positively charged phenylboronic acid in its side chains, which support the anti‐quenching of NIR‐II FL and the integration of DETA NONOate and CDDP into PBT/NO/Pt. In the acidic tumor microenvironment, the coordination bonds between CDDP and PNC11BA are cleaved, releasing CDDP for chemotherapeutic activity. The simultaneous release of nitric oxide (NO) and O_2_^•−^ rapidly leads to the in situ generation of the more cytotoxic reactive physiological nitrogen species ONOO^−^. In vitro and in vivo results prove that PBT/NO/Pt exhibited a markedly ONOO^−^ enhanced chemo–photothermal synergistic therapy for SKOV3/DDP tumor by downregulating the intracellular glutathione and increasing CDDP–DNA adducts.

## Graphical abstract

An NIR‐II (1000–1700 nm) excitation multifunctional ONOO^2212^ nanogenerator (PBT/NO/Pt) is developed by integrating non‐conjugated segments doping conjugated polyelectrolyte, cisplatin (CDDP), and NO donor (DETA NONOate) into an anti‐quenching NIR‐II excitation phenylboronic acid modified conjugated polyelectrolyte for NIR‐II fluorescence imaging and NIR‐II photoacoustic imaging guided synergistic NIR‐II photothermal, chemotherapy, and peroxynitrite therapy.

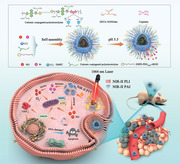

## 1 Introduction

Chemotherapy is the most common clinical strategy for cancer treatment. However, the efficacy of chemotherapeutic drugs such as doxorubicin, epirubicin, and platinum (Pt) is limited due to multidrug resistance, which consequently reducing patient survival.^[^ ^1^, ^2^, ^3^, ^4^, ^5^ ^]^ For example, Pt resistance occurs when Pt drugs bind to intracellular glutathione (GSH) and form GSH–Pt conjugates, which are subsequently exported from cancer cells via glutathione‐S‐conjugate pumps.^[^ ^6^, ^7^, ^8^, ^9^, ^10^, ^11^ ^]^ Photothermal therapy (PTT) ablates cancer cells through localized hyperthermia converted from near‐infrared (NIR) light by photothermal transfer agents.^[^ ^12^, ^13^, ^14^, ^15^ ^]^ PTT enhances cancer therapeutic selectivity and alleviates multidrug resistance by increasing tumor sensitivity to chemotherapy drugs.^[^ ^16^, ^17^ ^]^ Recent studies have confirmed that the combination of PTT with chemotherapy could overcome the limitations of chemotherapy alone and improve therapeutic outcomes.^[^ ^18^, ^19^ ^]^ However, PTT did not solve the problem of cancer cells’ low efficiently in responding to chemotherapeutic drugs. Thus, the key to enhancing chemotherapeutic efficacy in cancer is to eliminate multidrug resistance.

Reactive physiological nitrogen species (RNS) are active free radical molecules that can damage intercellular biomolecules (lipids, proteins, and DNA) through oxidation, nitration, and nitrosylation.^[^ ^20^, ^21^, ^22^ ^]^ The RNS peroxynitrite (ONOO^−^) is the product of a diffusion‐limited reaction between nitric oxide (NO) and superoxide (O_2_^•−^), and shows great interest in cancer therapy because it can directly induce cancer cells apoptosis by destroying mitochondrial function and causing DNA strand breaks.^[^ ^23^, ^24^ ^]^ In addition, the nitrification capacity of ONOO^−^ also leads to the denaturation of glutamine synthetase (GS) and DNA damage repair proteins, thereby inhibiting DNA repair and GSH‐related chemotherapy resistance.^[^ ^25^, ^26^, ^27^ ^]^ Several clinical anticancer drugs (such as DOX and Pt (II)) activate intracellular nicotinamide adenine dinucleotide phosphate oxidases (NOXs) to catalyze the conversion of nicotinamide adenine dinucleotide phosphate (NADPH) to NADP^+^ while converting O_2_ to O_2_^•−^. The generated O_2_^•−^ reacts with NO to produce ONOO^−^.^[^ ^28^, ^29^, ^30^, ^31^ ^]^ Therefore, the tumor‐targeted co‐delivery of DOX or Pt (II) with a NO donor to induce intracellular ONOO^−^ generation can be utilized to combat drug resistance and enhance chemotherapeutic efficacy.

Conjugated polymers (CPs) are based on donor–acceptor (D–A) structures and have been widely used as cancer drug‐delivery systems owing to their easily tunable structures, enhanced tumor‐targeting efficacy, and low biological toxicity. CPs also enable in vivo optical imaging, which can improve chemotherapeutic sensitivity and accuracy.^[^ ^32^ ^]^ In recent years, various CPs with near‐infrared II (NIR‐II, 1000–1700 nm) absorption have been developed for NIR‐II fluorescence (NIR‐II FL) imaging, NIR‐II photoacoustic (NIR‐II PA) imaging, and NIR‐II PTT.^[^ ^33^, ^34^, ^35^, ^36^ ^]^ However, these NIR‐II absorbing CPs lack functional groups for conjugating drug molecules, resulting in poor drug loading efficiency and uncontrolled drug release. NIR‐II absorbing CPs also exhibit low NIR‐II fluorescence quantum yield in aqueous solution due to the strong nonradiative thermal deactivation of the polymer and severe aggregation‐induced fluorescence quenching. Thus, there is an urgent need of novel NIR‐II absorbing CPs with functional group modifications for improving drug loading and enhancing NIR‐II fluorescence intensity, enabling precise NIR‐II FL and NIR‐II PA imaging, as well as imaging‐guided drug and NO donor delivery.

**Scheme 1:**
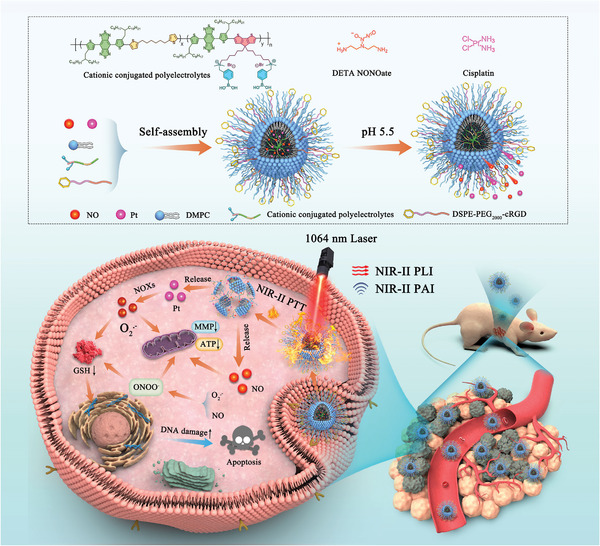
Schematic of NIR‐II light excitation multifunctional ONOO^−^ nanogenerator (PBT/NO/Pt) for NIR‐II FL/NIR‐II PA imaging‐guided synergistic NIR‐II PTT/chemotherapy/peroxynitrite therapy.

Cationic conjugated polyelectrolytes (CPEs) are positively charged flexible sidechain– modified CPs with outstanding capacity for loading chemotherapy drugs and nucleic acids. In addition, they can reduce the aggregation of the conjugated backbone to enhance fluorescence intensity.^[^ ^37^ ^]^ Herein, we reported an NIR‐II light excitation multifunctional ONOO^−^ nanogenerator (PBT/NO/Pt) for NIR‐II FL/NIR‐II PA imaging‐guided synergistic NIR‐II PTT/chemotherapy/peroxynitrite therapy (**Scheme** **1**). The PBT/NO/Pt was constructed by integrating phenylboronic acid modified NIR‐II conjugated polyelectrolytes (PNC11BA), a pH‐sensitive NO donor (DETA NONOate), and an NOX‐triggered O_2_^•−^‐generating chemotherapy drug (cisplatin, CDDP). First, we tactfully incorporated strong electron donors (CPDT) and non‐conjugated segments (C6) into the polymer backbone, as well as cationic phenylboronic acid groups with side chains to produce the NIR‐II polymer PNC11BA. Our prepared PNC11BA possess three significant advantages over previous NIR‐II conjugated polymers. First, the conjugated polyelectrolyte PNC11BA exhibits peak absorption at 1064 nm and high NIR‐II photothermal conversion efficiency (PCE) value (53.65%), which makes the PBT/NO/Pt suitable for NIR‐II PA imaging and NIR‐II PTT. Second, PNC11BA contains C6 segments in the polymer backbone that can simultaneously achieve a strong NIR‐II absorption extinction coefficient and NIR‐II fluorescence by reducing nonradiative decay. Third, the cationic side chains PBA can significantly reduce aggregation‐induced quenching (ACQ) in aqueous environments and result in PBT/NO/Pt with excellent NIR‐II fluorescence brightness when PNC11BA is carried by DMPC and DSPE–PEG_2000_–cRGD. Finally, the phenylboronic acid groups on the side chains of PNC11BA will enhance the loading efficiency of DETA NONOate and CDDP drugs in PBT/NO/Pt through donor‐acceptor interactions. In vitro studies show that the as‐prepared PBT/NO/Pt efficiently releases of NO and O_2_^•−^, as well as generation ONOO^−^ in the acidity and high NOXs concentration environment. The in situ generation of ONOO^−^ sensitizes resistant tumors to CDDP by downregulating intracellular GSH and increasing the level of CDDP–DNA adducts. PBT/NO/Pt also exhibited bright NIR‐II FL and NIR‐II PA imaging for in vivo tumor diagnosis, as well as synergistic therapy NIR‐PTT/ONOO^−^ for in vivo anti‐cancer application. More importantly, the remarkable NIR‐II PTT and chemotherapy enhanced by ONOO^−^ of PBT/NO/Pt endow them with a good suppression effect for CDDP‐resistant SKOV3 tumors.

## 2 Results and Discussion

### 2.1 Synthesis and Optical Properties of PBA‐Modified NDCPEs

**Scheme 2:**
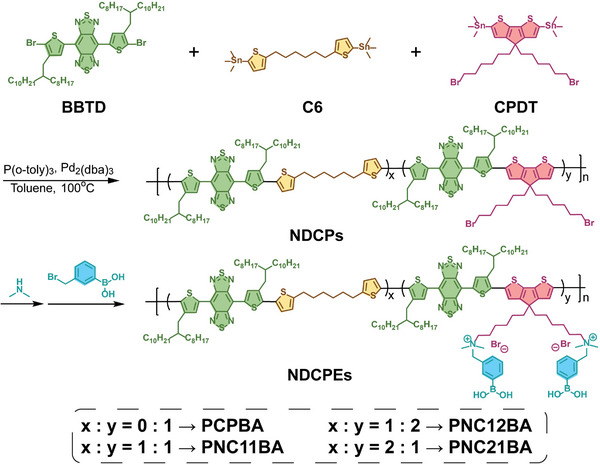
The synthetic routes of the phenylboronic acid–modified NDCPEs.

Three non‐conjugated segments doping conjugated polymers (NDCPs) with different conjugated segment densities were synthesized by Stille coupling polymerization between a strong electron acceptor (BBTD), an electron donor (CPDT), and the non‐conjugated unit (thiophen‐hexane, C6) (**Scheme** **2**). These copolymers, produced with different molar feed ratios of C6:CPDT at 2:1, 1:1, and 1:2, were named PNC21, PNC11, and PNC12, respectively. The electron donor CPDT in these polymers also has brominated side chains to allow side chain post‐functionalization. As shown in Scheme 2, NDCPs (PNC21, PNC11, and PNC12) were converted to phenylboronic acid–modified non‐conjugated segments doping conjugated polyelectrolyte (NDCPEs: PNC21BA, PNC11BA, PNC12BA) via two‐step reactions with dimethylamine and 3‐bromomethyl phenylboronic acid (PBA). Traditional CPs (PCP) and phenylboronic acid–modified conjugated polyelectrolyte (PCPBA) with BBTD as the electron acceptor and CPDT as the electron donor were also synthesized. The chemical structures of PNC21, PNC11, PNC12, PCP, PNC21BA, PNC11BA, PNC12BA, and PCPBA were confirmed by ^1^H NMR (Figures S1–S8, Supporting Information). Average molecular weights (M_n_) were around 10 000 g mol^−1^ for PNC21, PNC11, PNC12, and PCP copolymers as demonstrated by gel‐permeation chromatography (Figures S9–S12 and Table S1, Supporting Information).

**Figure 1:**
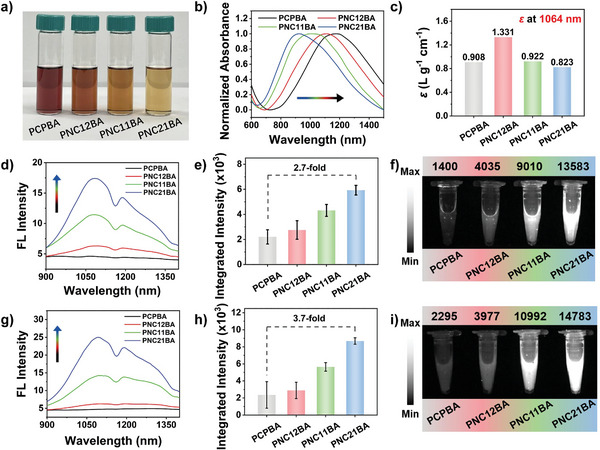
Characterization of NDCPEs (PCPBA, PNC12BA, PNC11BA, and PNC21BA) in DCM. a) Images of NDCPEs in DCM (1.0 mg mL^−1^). b) Normalized absorption spectra and c) *ɛ*_1064_ of NDCPEs. d) NIR‐II fluorescence spectra, e) quantified integrated intensity (range, 900–1400 nm), and f) NIR‐II FL images (808 nm excitation, 1064 nm long‐pass filter) and image intensities of NDCPEs at the same concentration. g) NIR‐II FL spectra, h) quantified integrated intensity (range, 900–1400 nm), and i) NIR‐II FL images (808 nm excitation, 1064 nm long‐pass filter) and image intensities of NDCPEs at the same 808 nm optical density. Error bars, mean ± SD.

As shown in Figure S13 (Supporting Information), NDCPs (PCP, PNC12, PNC11, and PNC21) are fully soluble at 0.1 mg mL^−1^ in tetrahydrofuran (THF). The polymer solutions changed from dull red (PCP) to brown (PNC21) after doping of nonconjugated segments. In contrast, the solubility of NDCPEs (PCPBA, PNC12BA, PNC11BA, and PNC21BA) with cationic phenylboronic acid side groups were dramatically reduced in THF, yielded aggregates (Figure S14, Supporting Information). As shown in **Figure** **1**a, NDCPEs (PCPBA, PNC12BA, PNC11BA, and PNC21BA) fully dissolved in dichloromethane (DCM) (1.0 mg mL^−1^). Figure 1b shows the UV–vis–NIR absorption spectra in DCM (0.1 mg mL^−1^). All four NDCPEs exhibited broad and intense absorbance at 800–1200 nm. Absorption peaks were blue‐shifted from 1177 to 925 nm with increasing non‐conjugated density in the polymer backbone from PCPBA to PNC21BA, due to the reduced conjugation length in polymer chain. The spectral properties of NDCPEs were similar to those of NDCPs (Figures S15 and S16, Supporting Information). As depicted in Figures 1c and Figure S17 (Supporting Information), the extinction coefficients of PCPBA, PNC12BA, PNC11BA, and PNC21BA at 1064 nm were 0.908, 1.331, 0.922, and 0.823 L g^−1^ cm^−1^, respectively. The NIR‐II fluorescence spectra of the polymers in DCM at the same concentration are shown in Figure 1d, and the non‐conjugated segments doped PNC12BA, PNC11BA, and PNC21BA polymers show similar NIR‐II emission spectra and an emission peak at 1092 nm. In contrast, PCPBA did not have a distinctive NIR‐II emission spectrum. The NIR‐II emission intensity increased with the increasing density of non‐conjugated C6 in polymer chain, 2.7‐fold greater at 1092 nm from PNC12BA to PNC21BA at the same concentration (Figure 1e). The NIR‐II fluorescence imaging results in Figure 1f show that PNC21BA emitted the strongest signals at the same concentration, 9.7‐fold greater than PCPBA. It was interesting to note that when the 808 nm absorption intensity was normalized, the NIR‐II fluorescence intensity of the NDCPEs gradually increased with the increasing doping density of non‐conjugated C6 units (Figure 1 g; Figure S18, Supporting Information). The NIR‐II fluorescence intensity of the maximum emission peak and the imaging intensity for PNC21BA were ≈3.7 and 6.4 times stronger than PCPBA (Figure 1h,i). These findings showed that non‐conjugated monomer doping in the conjugated backbone improves the NIR‐II emission of the conjugated polymer. It should be noted that similar NIR‐II fluorescence amplification was also detected for the NDCPs (Figure S19, Supporting Information).

### 2.2 Ionization‐Enhanced NIR‐II Fluorescence in NDCPEs‐Based Nanoparticles

**Figure 2:**
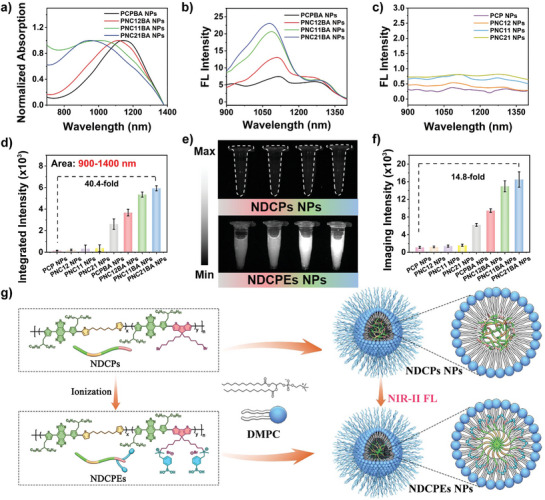
a) Absorption and b) NIR‐II fluorescence spectra (Ex: 808 nm) of NDCPEs NPs (PCPBA NPs, PNC12BA NPs, PNC11BA NPs, and PNC21BA NPs). c) NIR‐II fluorescence spectra of NDCPs NPs (PCP NPs, PNC12 NPs, PNC11 NPs, and PNC21 NPs) at the same concentration as b). d) Corresponding quantified fluorescence integrated intensity (range, 900–1400 nm) of (b) and (c). e) NIR‐II FL images (Ex: 808 nm; 1064 nm long‐pass filter) and f) corresponding imaging intensities of NDCPs NPs (up, left to right: PCP, PNC12, PNC11, and PNC21) and NDCPEs NPs (down, left to right: PCPBA, PNC12BA, PNC11BA, and PNC21BA) at the same concentration. g) Schematic diagram of NDCPs NPs and NDCPEs NPs assembly in aqueous solution. Error bars, mean ± SD.

Previously reported conjugation polymers exhibited ACQ and significantly reduced NIR‐II FL intensity, making it difficult to use them for biomedical imaging applications. Our phenylboronic acid–modified NDCPEs were encapsulated in amphiphilic liposomes (1,2‐dimyristoyl‐sn‐glycero‐3‐phosphocholine, DMPC) to form water‐soluble nanoparticles (PCPBA NPs, PNC12BA NPs, PNC11BA NPs, and PNC21BA NPs) through nanoprecipitation. Four NDCPs NPs (PCP NPs, PNC12 NPs, PNC11 NPs, and PNC21 NPs) were also prepared using a similar method for comparison. In solution, these water‐soluble nanoparticles exhibit colors (Figures S20 and S21, Supporting Information). As shown in **Figures** **2**a and S22 (Supporting Information), these nanoparticles all have broad NIR‐II absorption spectra from 980 to 1350 nm. In contrast, PCPBA NPs, PNC12BA NPs, PNC11BA NPs, and PNC21BA NPs are red‐shifted compared to PCP NPs, PNC12 NPs, PNC11 NPs, and PNC21 NPs. Furthermore, a difference was observed in the NIR‐II fluorescence spectra of the eight nanoparticles (Figure 2b,c). Only NDCPEs NPs exhibited significant NIR‐II fluorescence, suggesting that NDCPEs may have lower aggregation profiles in liposome nanoparticles and improved NIR‐II emission. More importantly, the increase in non‐conjugation segment density in the NDCPEs results in a higher NIR‐II FL intensity for the water‐soluble nanoparticles, with PNC21BA NPs having the strongest NIR‐II emission intensity (40.4‐fold stronger than that of PCP NPs) (Figure 2d). NIR‐II FL imaging results in Figure 2e,f illustrate that PNC21BA NPs emit the strongest signals at the same concentration, a 14.8‐fold improvement compared to PCP NPs. Similarly, PNC21BA NPs had the strongest NIR‐II emission intensity at the same 808 nm absorbance (Figure S23, Supporting Information). Figures S24 and S25 (Supporting Information) show the extinction coefficients of NDCPs NPs and NDCPEs NPs. We compared the NIR‐II fluorescence intensities of PCPBA NPs, PNC12BA NPs, PNC11BA NPs, and PNC21BA NPs in aqueous solution with PCPBA, PNC12BA, PNC11BA, and PNC21BA in DCM at the same concentration to measure the degree of quenching (Figures S26 and S27, Supporting Information). The NIR‐II fluorescence quenching yield ratios of the nanoparticles relative to the polymers in DCM are plotted in Figure S27 (Supporting Information). The NIR‐II FL intensity of the PCPBA NPs in aqueous solution reached 60% of the polymer levels in DCM, indicating that the NIR‐II fluorescence of the NDCPEs was largely retained in the nanoparticles with only moderate quenching. Fluorescence was at 105% for PNC21BA NPs versus PCPBA NPs (60%), supporting the hypothesis that the nonconjugated segments in the polymer backbone reduce NIR‐II fluorescence quenching. These results also show that the NIR‐II emission properties of NDCPEs NPs could be fine‐tuned by regulating the density of non‐conjugated segments in the polymer backbone. The difference in NIR‐II optical properties demonstrates the significant effect of cationic PBA modification in the NDCPEs side chain on NIR‐II fluorescence via ionic interaction between amphiphilic liposomes inhibiting intermolecular aggregation (Figure 2g).

### 2.3 Preparation and Characterization of PBT/NO/Pt

**Figure 3:**
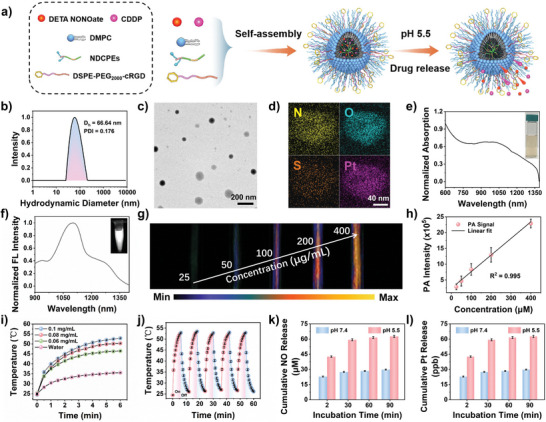
Characterization of PBT/NO/Pt in aqueous solution. a) Schematic diagram of PBT/NO/Pt self‐assembly in aqueous solution. b) *D*_h_ of PBT/NO/Pt measured by DLS. c) TEM image of PBT/NO/Pt (scale bar: 200 nm). d) TEM‐EDS element mapping of PBT/NO/Pt (scale bar: 40 nm). e) Normalized absorption spectra of PBT/NO/Pt. Inset: Photograph of PBT/NO/Pt solutions. f) Normalized NIR‐II FL spectra of PBT/NO/Pt. Inset: NIR‐II FL imaging of PBT/NO/Pt. g) In vitro PA images of varying concentrations of PBT/NO/Pt under 1064 nm irradiation. h) Linear relationship between PA signal and PBT/NO/Pt concentration (*n* = 3). i) Photothermal heating curves of water and PBT/NO/Pt at 0.1, 0.08, and 0.06 mg mL^−1^ under 1064 nm laser irradiation (1.0 W cm^−2^) (*n* = 3). j) Photothermal stability of PBT/NO/Pt over five on–off cycles (*n* = 3). k) In vitro NO and l) Pt release profiles for PBT/NO/Pt upon different treatments (*n* = 3). Error bars, mean ± SD (*n* = 3).

Given the excellent 1064 nm extinction coefficient of PNC11BA and bright NIR‐II fluorescence of PNC11BA NPs, we used them to prepare phototheranostic agents. The PBA group on the side chain of PNC11BA was easily loaded with cisplatin (CDDP) and the NO donor (DETA NONOate) via donor–acceptor coordination. As shown in **Figure** **3**a, self‐assembly of PNC11BA, CDDP, DETA NONOate, DMPC, and DSPE‐PEG_2000_‐cRGD were realized by conventional precipitation, affording multifunctional phototheranostic nanoparticles (named PBT/NO/Pt). CDDP was loaded on these nanoparticles as the chemotherapy drug and NOX active O_2_^•−^ generator. DETA NONOate was selected as the pH‐responsive NO donor. PNC11BA NPs loaded with DETA NONOate or CDDP alone or without DETA NONOate and CDDP loading were prepared as controls (denoted as PBT/NO, PBT/Pt, and PBT). Dynamic light scattering (DLS) showed that the hydrodynamic diameter (*D*_h_) of PBT/NO/Pt was 66.64 nm with a low PDI of 0.176 (Figure 3b). As obtained PBT/NO/Pt possessed spherical nanoparticles with the diameter of 80 nm measured by transmission electron microscopy (TEM) (Figure 3c). DLS results and TEM images of PBT, PBT/NO, and PBT/Pt showed good dispersion (Figures S28 and S29, Supporting Information), and the *D*_h_ of PBT/NO/Pt remained nearly constant in various aqueous media, including PBS buffer, fetal bovine serum (FBS), and Dulbecco's Modified Eagle Medium (DMEM), validating the dispersibility of PBT/NO/Pt (Figure S30, Supporting Information). The *D*_h_ of PBT/NO/Pt in PBS changed very little over 5 days at 4 °C, supporting its stability under storage (Figure S31, Supporting Information). Energy dispersive X‐ray element mapping (EDS) showed the Pt elements were well distributed across nanoparticles, demonstrating successful CDDP loading (Figure 3d; Figure S32, Supporting Information). According to the standard curves obtained with different concentrations by microplate reader and inductively coupled plasma mass spectrometer (ICP‐MS), the loading efficiency of DETA NONOate and CDDP in PBT/NO/Pt was calculated to be 5.42% and 3.18%, respectively (Figures S33 and S34, Supporting Information).

As shown in Figure 3e, PBT/NO/Pt in aqueous solution exhibited strong NIR absorption at 800–1350 nm, and the extinction coefficient was 1.195 L g^−1^ cm^−1^ at 1064 nm (Figure S35, Supporting Information). Under 808 nm light excitation in aqueous solution, PBT/NO/Pt showed strong NIR‐II fluorescence centered at 1101 nm with a long tail extending to 1350 nm (Figure 3f). NIR‐II FL intensity remained nearly constant over 60 min (808 nm, 0.5 W cm^−2^), indicating that PBT/NO/Pt is photostable (Figure S36, Supporting Information). Figure 3g shows NIR‐II PA images of PBT/NO/Pt at varying concentrations. The NIR‐II PA signals demonstrated an excellent linear relationship with NPs concentration (Figure 3h). The photothermal performance of PBT/NO/Pt was verified under 1064 nm laser irradiation (1.0 W cm^−2^). As shown in Figure 3i, the temperature of the aqueous solution increased with increasing PBT/NO/Pt concentration. For example, the temperature increased to 53 °C within 6 min at 0.1 mg mL^−1^ PBT/NO/Pt, and the PCE was calculated to be 53.65% (Figures S37 and S38, Supporting Information). In addition, the stability of PBT/NO/Pt under photothermal effect was faithfully proved, the temperature changes of PBT/NO/Pt solution were smaller than 2 °C upon five times light on/off irradiation cycles (Figure 3j).

We next explored the acid‐responsive release profiles of DETA NONOate and CDDP from the PBT/NO/Pt. A typical Griess assay was used to quantify DETA NONOate release in aqueous solution at pH 7.4 and pH 5.5. As shown in Figure 3k, NO generation from PBT/NO/Pt was minimal after 90 min at pH 7.4. In contrast, NO release was more than 60 µm after 90 min at pH 5.5, reflecting acid‐responsive dissociation of donor–acceptor coordination interactions between DETA NONOate and the PBA groups and DETA NONOate degradation. Acid‐responsive release of CDDP from PBT/NO/Pt is a critical step in this design, since CDDP can only activate NOXs to produce O_2_^•−^ in the weakly acidic intracellular environment. Release of CDDP from PBT/NO/Pt was evaluated by ICP‐OES (Figure 3l), which showed negligible release after 90 min at pH 7.4, while more than 60% of CDDP was released at pH 5.5 due to the accelerated dissociation of the amino and PBA groups. These results suggest unfavorable DETA NONOate and CDDP leakage from PBT/NO/Pt could be limited during blood circulation, supporting the targeted release of NO and CDDP in the acidic tumor tissue and cancer cells.

### 2.4 NO, O_2_^•−^, and ONOO^−^ Generation in Cancer Cells

**Figure 4:**
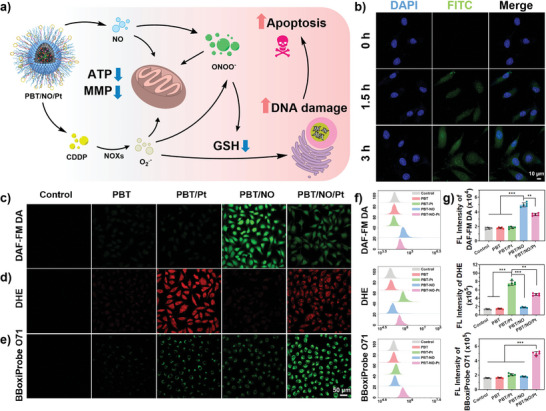
Intracellular generation of NO, O_2_^•−^, and ONOO^−^. a) Schematic illustration of in vitro ONOO^−^ generation by PBT/NO/Pt. b) CLSM imaging of SKOV3/DDP cells stained with FITC/PBT/NO/Pt (green) and DAPI (blue) (scale bar: 10 µm). CLSM images of SKOV3/DDP to evaluate c) intracellular NO generation detected by probe DAF‐FM DA (green); d) intracellular ROS generation detected by O_2_^•−^ probe DHE (red); e) intracellular ONOO^−^ generation detected by probe BBoxiProbe O71 (green) (scale bar: 50 µm). f) Quantitative analysis of intracellular NO, O_2_^•−^, and ONOO^−^ levels by flow cytometry. g) Quantification of the FL Intensity from (f). (*n* = 5). Data are represented as mean ± standard deviation (SD) (^*^ *p* < 0.05, ^**^ *p* < 0.01, ^***^ *p* < 0.001).

**Figure** **4**a shows how intracellular NO, O_2_^•−^, and ONOO^−^ are produced. The cellular uptake of PBT/NO/Pt was first examined by confocal laser scanning microscopy (CLSM) and flow cytometry. Upon incubation with FITC dyes loaded PBT/NO/Pt for 1.5 h, substantial green fluorescence was found in SKOV3/DDP cells. Much stronger green fluorescence was observed after the incubation time extending to 3 h, indicating the cellular entrance of PBT/NO/Pt (Figure 4b; Figure S39). Given the demonstrated acid‐triggered NO generation and CDDP release, intracellular NO, O_2_^•−^, and in situ ONOO^−^ formation were then monitored. A commercial NO fluorescence probe (DAF‐FM DA) was used to evaluate intracellular NO levels. As shown in Figure 4c, stronger green fluorescence was observed after 3 h incubation of SKOV3/DDP cells with PBT/NO/Pt and PBT/NO due to acid‐accelerated decomposition of DETA NONOate. In comparison, PBT/Pt and PBT‐treated SKOV3/DDP cells and controls exhibited very weak fluorescence, indicating the lack of NO generation from these nanoparticles. CDDP is released in the acidic intracellular environment, then catalyzes NOXs to produce O_2_^−^. We monitored intracellular O_2_^•−^ levels in SKOV3/DDP cells with the O_2_^•−^ probe dihydroethidium (DHE). As depicted in Figure 4d, significant red fluorescence (O_2_^•−^) was observed in PBT/NO/Pt and PBT/Pt treated SKOV3/DDP cells, indicating efficient intracellular O_2_^•−^ production. However, due to the lack of CDDP, no red fluorescence was detected in PBT/NO and PBT‐treated SKOV3/DDP cells and controls. The combination of NO and O_2_^•−^ produces ONOO^−^, therefore, intracellular ONOO^−^ was measured using a commercial probe BBoxiProbe O71. As shown in Figure 4e, SKOV3/DDP cells incubated with PBT/NO and PBT/Pt showed no significant green fluorescence due to the absence of O_2_^•−^ and NO, respectively. As expected, green fluorescence was observed in SKOV3/DDP cells incubated with PBT/NO/Pt, indicating the generation of abundant ONOO^−^ by in situ production of NO and O_2_^•−^. The similar intracellular NO, O_2_^•−^ and ONOO^−^ generation from PBT, PBT/NO/Pt, PBT/NO and PBT/NO/Pt treated SKOV3/DDP cells was further quantitative statistics investigated by flow cytometric analysis (Figure 4f,g), which agreed with above CLSM imaging results.

### 2.5 In Vitro Cancer Cell Inhibition and Action Mechanism of PBT/NO/Pt

**Figure 5:**
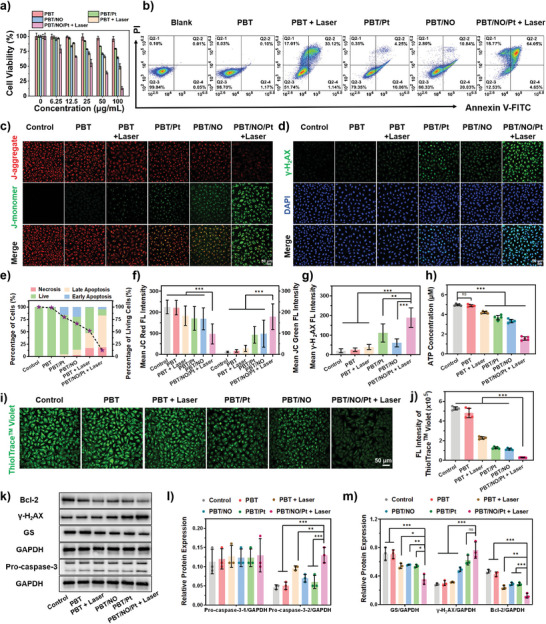
In vitro evaluation of the anticancer activity and mechanism of PBT/NO/Pt in SKOV3/DDP cells. a) Viability of SKOV3/DDP cells by MTT assay after different treatments (*n* = 5). b) Cell apoptosis and necrosis were analyzed by flow cytometry with Annexin V‐FITC/PI double staining after different treatments. c) CLSM images of JC‐1 stained SKOV3/DDP cells after different treatments (scale bar: 50 µm). d) γ‐H_2_AX assays showed the DNA double‐strand break status of SKOV3/DDP cells after different treatments (scale bar: 50 µm). e) Cell death stages were analyzed by flow cytometry and the survival probability of viable cells under different treatment conditions. f) Fluorescence quantitation of JC red and JC green from (c) (analyzed by Image J). g) Fluorescence quantitation of γ‐H_2_AX from (d) (analyzed by Image J). h) Quantification of adenosine triphosphate (ATP) in SKOV3/DDP cells after different treatments (*n* = 5). i) CLSM images of ThioTracker Violet stained SKOV3/DDP cells after different treatments (scale bar: 50 µm). j) Quantitative flow cytometry of GSH fluorescence (*n* = 5). k) Bcl‐2, Pro‐caspase‐3, γ‐H_2_AX, and GS protein expression in SKOV3/DDP cells. l) Relative expression of Pro‐caspase‐3 (protein/GAPDH, analyzed by Image J, *n* = 3). m) Relative expression of GS, γ‐H_2_AX, and Bcl‐2 (protein/GAPDH, analyzed by Image J, *n* = 3). (The “Control” group is the untreated cells). Data are represented as mean ± standard deviation (SD). (No significance: n.s., ^*^ *p* < 0.05, ^**^ *p* < 0.01, ^***^ *p* < 0.001).

PBT/NO/Pt triggered the formation of NO, O_2_^•−^, and ONOO^−^ in cancer cells. We next tested the anticancer effect of PBT/NO/Pt in SKOV3/DDP cells by the methyl thiazolyl tetrazolium (MTT) assay. As shown in **Figure** **5**a, viability decreased slightly in the PBT treated group. In contrast, survival in PBT/Pt, PBT/NO, and PBT + Laser treated SKOV3/DDP cells fell to 79.1%, 64.3%, and 49.8% at 100 µg mL^−1^. Among all groups, the 1064 nm laser (6 min, 1.0 W cm^−2^) treated PBT/NO/Pt group exhibited the strongest inhibition (cell survival fell to 13.38%), demonstrating the excellent synergistic effect of NIR‐II PTT and ONOO^−^ in PBT/NO/Pt. Apoptosis and necrosis induced by PBT/NO/Pt were also analyzed by flow cytometry (Figure 5b), which also demonstrated the maximum cytotoxicity was found in PBT/NO/Pt + Laser group with the highest apoptosis ratio about 87%. In contrast, PBT/NO, PBT/Pt, and PBT + Laser induced only moderate levels of apoptosis. These results demonstrate the effective anticancer activity of PBT/NO/Pt in SKOV3/DDP cells via codelivery NO and CDDP and in situ generation of ONOO^−^ (Figure 5e).

To visualize the therapeutic efficacy of PBT/NO/Pt, mitochondrial damage was evaluated by JC‐1 staining. Confocal imaging and quantitation showed the brightest green fluorescence in the PBT/NO/Pt + Laser treated group, suggesting the loss of mitochondrial membrane potential; similar results were obtained by flow cytometry (Figure 5c,f; Figure S40, Supporting Information). Intracellular ATP levels declined (about 30%) in the PBT/NO/Pt + Laser group, perhaps due to the inhibition of mitochondrial function (Figure 5h; Figure S41, Supporting Information). The extent of DNA damage caused by PBT/NO/Pt was assessed using γ‐H_2_AX, a conventional marker of DNA double‐strand breaks. The brightest green fluorescence was observed in PBT/NO/Pt + Laser cells, suggesting the efficient formation of CDDP–DNA adducts and more severe DNA damage by ONOO^−^ (Figure 5d,g). Subsequently, the therapeutic mechanism of PBT/NO/Pt was explored in SKOV3/DDP cells. It was earlier reported that CDDP could form Pt‐GSH complexes and decrease the generation of CDDP‐DNA adducts, thus inducing the detoxification of CDDP. The influence of ONOO^−^ formation by PBT/NO/Pt on intracellular GSH was measured in SKOV3/DDP cells using ThioTracker Violet. In Figure 5i, bright green fluorescence is observed in the blank control and PBT, PBT/NO, and PBT/Pt‐treated SKOV3/DDP cells. In comparison, PBT/NO/Pt + Laser treated SKOV3/DDP cells showed significantly less green fluorescence, indicating a loss of GSH content. Similar results were obtained by flow cytometry and quantitative analysis, which showed that PBT/NO/Pt + Laser reduced intracellular GSH (Figure 5j; Figure S42, Supporting Information). Thus, ONOO^−^ down‐regulates intracellular GSH, which improves CDDP binding to target DNA and promotes cancer cell apoptosis.

We investigated the expression of related proteins (Pro‐caspase‐3, Bcl‐2, γ‐H2AX, and GS) by western blotting (Figure 5k,l,m). Cleaved caspase‐3 expression was significantly induced and Bcl‐2 was downregulated in SKOV3/DDP cells treated with PBT/NO/Pt and 1064 nm light, indicating more cells initiated apoptosis (Figure 5k,l). As expected, the expression of γ‐H_2_AX and GS was consistent with the previous results, explaining why PBT/NO/Pt + Laser irradiation caused more serious DNA damage. These results confirmed that in situ ONOO^−^ generation downregulated intracellular GSH and increased the level of CDDP–DNA adducts and cleaved caspase‐3 to promote apoptosis.

### 2.6 In vivo NIR‐II FL/NIR‐II PA Imaging‐Guided Cancer Therapy with PBT/NO/Pt

**Figure 6:**
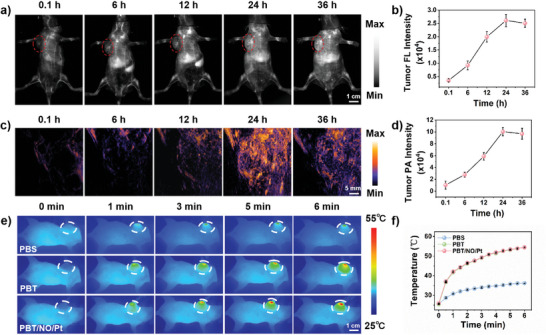
In vivo NIR‐II FL, NIR‐II PA, and photothermal imaging of SKOV3/DDP tumor‐bearing mice. a) NIR‐II FL (Ex: 808 nm, 1064 nm LP filter with 300 ms exposure time, scale bar: 1.0 cm) and c) NIR‐II PA (ex: 1064 nm, scale bar: 5 mm) images of mice upon intravenous injection with PBT/NO/Pt. b) Quantification NIR‐II FL images intensity of tumor sites in (a) (*n* = 3). d) Quantification NIR‐II PA images intensity of tumor sites in (c) (*n* = 3). e) Infrared thermal images of mice (scale bar: 1.0 cm) and f) tumor site temperature variation 24 h postinjection with PBT/NO/Pt or PBS upon 1064 nm laser irradiation (1.0 W cm^−2^) (*n* = 3). Data represent mean ± standard deviation (SD).

The in vivo anticancer efficiency of PBT/NO/Pt was investigated. Prior to the in vivo experiments, PBT/NO/Pt accumulation at tumor sites was visualized by NIR‐II FL and NIR‐II PA imaging systems. SKOV3/DDP tumor xenograft‐bearing mice were treated with intravenous PBT/NO/Pt (150 µL, 2.0 mg mL^−1^) and images were acquired at 0.1, 6, 12, 24, and 36 h postinjection. As shown in **Figure** **6**a,c, NIR‐II FL and NIR‐II PA signals in the tumor sites became bright at 6 h and reach maxima at 24 h. We concluded that NIR‐II FL and NIR‐II PA imaging could provide precise guidance for 1064 nm laser treatment. Figure 6b,d shows the quantitative imaging analysis of tumor sites, in which imaging intensity was 7.3‐ and 9.4‐fold higher than before injection in NIR‐II FL and NIR‐II PA, respectively, indicating PBT/NO/Pt accumulation at the tumor site via the enhanced permeability and retention (EPR) effect. At 24 h after injection, NIR‐II FL and NIR‐II PA images of the tumors and major organs were obtained (Figures S43 and S44, Supporting Information). Ex vivo data showed that more PBT/NO/Pt accumulated in the tumor sites than in other tissues, including the metabolizing organs. Additionally, typical in vivo photothermal imaging of tumor sites was performed at 24 h post‐injection with PBT/NO/Pt (Figure 6e,f). The tumor temperature gradually increased after 1064 nm laser irradiation (1.0 W cm^−2^) and peaked at 53 °C at about 6 min. In tumor‐bearing mice treated with PBS, no significant temperature increase was detected.

**Figure 7:**
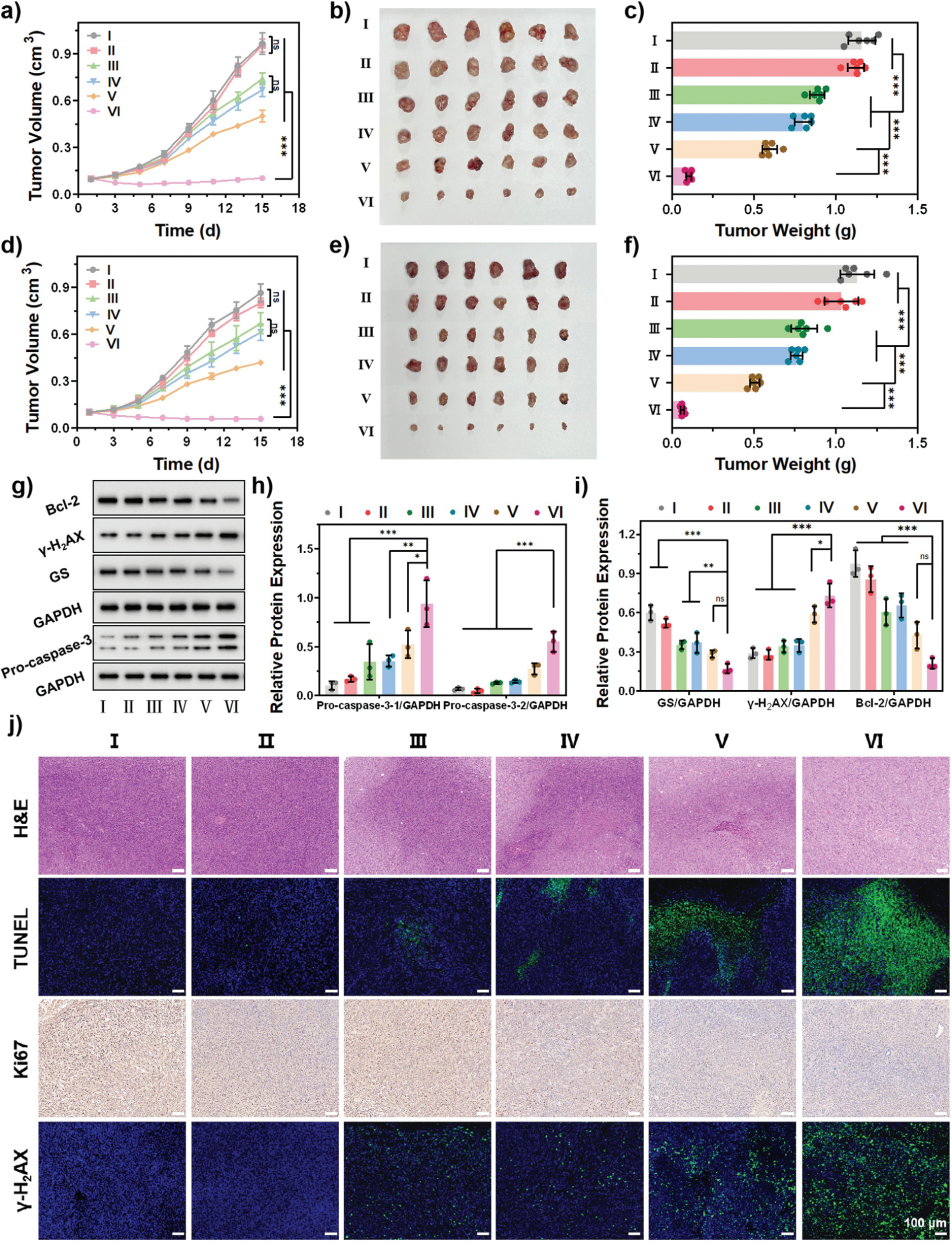
In vivo tumor inhibition against SKOV3 and SKOV3/DDP tumor‐bearing mice. a) Tumor growth curves of SKOV3 xenografted mouse models upon different treatments (*n* = 6). b) Tumor images of SKOV3 xenografted tumors collected at day 15 after diverse treatments. c) Tumor weight of SKOV3 xenografted mouse after diverse treatments (*n* = 6). d) Tumor growth curves of SKOV3/DDP xenografted mouse models upon different treatments (*n* = 6). e) Tumor images of SKOV3/DDP xenografted tumors collected at day 15 after diverse treatments. f) Tumor weight of SKOV3/DDP xenografted mouse after diverse treatments (*n* = 6). g) Western blot. h) Relative expression of pro‐caspase‐3 (*n* = 3). i) Relative expression of GS, γ‐H_2_AX, and Bcl‐2 (*n* = 3). j) H&E staining, TUNEL staining, and immunohistochemical analysis of Ki67 and γ‐H_2_AX staining of tumor tissues after different treatments. TUNEL and γ‐H_2_AX‐positive cells are all stained green. (Scale bar: 100 µm). (I: PBS, II: PBT, III: PBT/Pt, IV: PBT/NO, V: PBT + Laser, VI: PBT/NO/Pt + Laser, (1.0 W cm^−2^)). Data are represented as mean ± standard deviation (SD). (No significance: n.s., ^*^ *p* < 0.05, ^**^ *p* < 0.01, ^***^ *p* < 0.001).

In vivo cancer inhibition by PBT/NO/Pt was evaluated in two subcutaneous tumor models (SKOV3 and SKOV3/DDP xenograft‐bearing nude mice). The tumor‐bearing mice were randomly divided into six groups: (I) Control, (II) PBT, (III) PBT/Pt, (IV) PBT/NO, (V) PBT + Laser, and (VI) PBT/NO/Pt + Laser. Tumor size was monitored every 2 days after intravenous administration of PBS, PBT, PBT/Pt, PBT/NO, or PBT/NO/Pt (150 µL, 2.0 mg mL^−1^). At 24 h postinjection, tumor sites in the PBT + Laser and PBT/NO/Pt + Laser groups were exposed to 1064 nm laser (1.0 W cm^−2^) irradiation for 6 min. As shown in **Figures** **7**a and S45 (Supporting Information), after 15 days, compared with the control group (10.2‐fold tumor volume increase), PBT/Pt exhibited very weak anticancer efficacy (7.4‐fold tumor volume increase). PBT/NO and PBT + Laser groups showed a 7.0‐ and 5.2‐fold increase in tumor volume, respectively, indicating single NO gas and NIR‐II PTT therapy provided better tumor growth inhibition than PBT/Pt. In sharp contrast, PBT/NO/Pt + Laser yielded the best tumor suppression capacity due to the synergistic effect of Pt, NO, and NIR‐II PTT as well as the ONOO^−^ burst. After 15 days, the tumors were excised from each group and imaged (Figure 7b). The weights and volumes of the collected tumors were also recorded. The average tumor weights were 1.16, 1.12, 0.89, 0.80, 0.60, and 0.10 g for control, PBT, PBT/Pt, PBT/NO, PBT + Laser, and PBT/NO/Pt + Laser, respectively, confirming the remarkable cancer inhibitory activity of PBT/NO/Pt after 1064 nm laser irradiation (Figure 7c). Figure 7d–f illustrates the outstanding cancer inhibition of PBT/NO/Pt + Laser in SKOV3/DDP tumor‐bearing mice. We further detected expression of apoptosis‐related proteins (Pro‐caspase‐3, Bcl‐2, γ‐H_2_AX, and GS) in SKOV3 tumors isolated on day 15 (Figure 7g,h,i). Consistent with the previous results, PBT/NO/Pt + Laser downregulated GSH, led to more severe DNA damage, and inhibited tumor growth.

To explore the tumor inhibition capacity of PBT/NO/Pt, histologic sections were analyzed by hematoxylin–eosin (H&E) staining, terminal deoxynucleotidyl transferase dUTP nick‐end labeling (TUNEL) staining, immunohistochemistry (Ki‐67 antibody staining), and immunofluorescence staining of γ‐H_2_AX. As shown in Figure 7j, PBT/NO/Pt + Laser induced the strongest apoptotic response, tumor suppression, and DNA damage, verifying the amplified therapeutic efficiency of PBT/NO/Pt. These results demonstrate that CDDP and DETA NONOate co‐delivered by PBT/NO/Pt caused DNA damage and cell apoptosis by generating ONOO^−^ and the synergistic effects of Pt, NO, and NIR‐II PTT.

Finally, the safety profile of PBT/NO/Pt was evaluated. Body weights did not change significantly throughout the experiment (Figure S47 and S48, Supporting Information). Histologic analysis of the major organs is shown in Figure S49 (Supporting Information), no pathological abnormalities were observed in the treatment groups versus the controls, suggesting the minimal side effects of PBT/NO/Pt. Finally, the blood hematology and biochemistry indices showed normal liver and kidney functions (Figure S50, Supporting Information), again demonstrating the biocompatibility of PBT/NO/Pt. In this work, we developed a phototheranostic platform PBT/NO/Pt and shows several advantages for clinical application potential. Conjugated polymer PNC11BA has intrinsic biocompatibility and can achieve the bio‐degradability by rational design of the polymer backbone. PBT/NO/Pt displays excellent selectivity and sensitivity to the acidic and high NOXs expressed in the tumor microenvironment, which benefits the elimination of cancer with multidrug resistance. PBT/NO/Pt holds excellent photo and colloidal stability.

## 3 Conclusion

We constructed an NIR‐II light‐triggered multifunctional ONOO^−^ nanogenerator (PBT/NO/Pt) for synergistic NIR‐II PTT/chemotherapy/peroxynitrite therapy guided by NIR‐II FL/NIR‐II PA imaging. PBT/NO/Pt exhibited strong NIR‐II absorption, excellent NIR‐II fluorescence emission, intense NIR‐II PA signal under 1064 nm laser irradiation, and excellent PCE (*η* = 53.65%). Moreover, PBT/NO/Pt reaches the tumor site, and the acidity and high concentration of NOXs in the tumor microenvironment enable simultaneous release of NO and O_2_^•−^ and subsequent formation of ONOO^−^ in situ. This in situ generation of ONOO^−^ has a significant anticancer effect against SKOV3/DDP cells through downregulation of intracellular GSH and an increase in DNA damage. In vitro and in vivo studies have shown that PBT/NO/Pt had considerable anti‐SKOV3 and SKOV3/DDP tumor‐suppressive effects under NIR‐II PTT enhanced by ONOO^−^ and chemotherapy. Therefore, the developed NIR‐II light excitation multifunctional ONOO^−^ nanogenerator is a promising strategy for treating drug‐resistant tumors.

## 4 Experimental Section

### Synthesis of PCP, PNC12, PNC11, and PNC21

Chemical of 4,8‐bis (5‐bromo‐4‐ (2‐octyldodecyl) thiophene‐2‐yl) – benzo [1,2‐c: 4,5‐c ‘] bis [1,2,5] thiadiazole, 1,6‐bis (5‐trimethylbenzoyl) thiophene‐2‐yl) hexane, (4,4‐ bis (6‐ bromohexyl) −4H‐ cyclopentadieno [2,1‐b:3,4‐b’] dithiene −2,6‐ diyl) bis (trimethylstannane), Pd_2_(dba)_3_, P(o‐tol)3 and anhydrous toluene (2.5 mL) were added into a polymerization tube to keep the whole system in an anhydrous and oxygen‐free environment. Then, it is placed in an oil bath and the temperature is kept at 100 °C for the reaction, which ends after 4 h. Cool it to room temperature, extract the reaction solution, settle with methanol, then filter, and finally obtain a black solid as the final product.

### Synthesis of PCPBA, PNC12BA, PNC11BA, and PNC21BA

Take the corresponding products in the previous step, put them into a round‐bottomed flask, add ultra‐dry tetrahydrofuran into the flask, and slowly add dimethylamine solution under the condition of an external ice bath after three times of vacuumizing and blowing nitrogen. After reacting in an ice bath for 1 h, the reaction bottle was put into an oil bath pot at 50 °C, and after 3 days of reaction, the solvent was removed by rotary evaporation. Add ultra‐dry tetrahydrofuran and 3‐Bromomethylphenylboronic acid into the reaction bottle. React for three days under the condition of removing oxygen and water.

### Cell Culture

SKOV3/DDP, a Human ovarian cancer cell line cisplatin‐resistant strain was cultured with McCoy's 5A supplemented with 10% fetal bovine serum (FBS) in a humidified 5% CO_2_ environment at 37 °C.

### Animal Experiments

The animal protocols used in this study were approved by the Institutional Committee on the Ethics of Animal Experiments of Zhengzhou University (2023‐KY‐0433‐001, Zhengzhou, China). Five to six‐week‐old BALB/c mice were obtained from the Shanghai Laboratory Animal Center with pathogen‐free feeding environment. SKOV3 or SKOV3/DDP tumors were established in female BALB/c nude mice by injecting SKOV3 or SKOV3/DDP cells mixed with 50 µL of PBS under the skin beside the left armpit. The tumor volume was measured using the equation V = 0.5 L W^2^, where L refers to the longitudinal diameter and W indicates the transverse diameters of the tumors.

### Histological Analysis

All major organs (heart, liver, spleen, lung, and kidney) and tumors were achieved from healthy ICR mice (*n* = 3 per group) at 15 days. They were embedded in an optimal cutting temperature sample (Tissue‐Tek, Sakura Finetek, USA). After that, these samples were sliced into 4 µm sections with a microtome in the cryostat at −20 °C and then transferred to a microscope slide for analysis of hematoxylin‐eosin (H&E).

### Graphpad Prism was used for Statistical Analysis

Quantitative data were performed for at least three times and the data were expressed as mean ± standard deviation (SD). Data were analyzed for statistical significance using Student's *t*‐test. ^*^ *p* < 0.05 was considered statistically significant, while ^*^ *p* < 0.05, ^**^ *p* < 0.01, ^***^ *p* < 0.001 were considered highly and extremely significant.

## Conflict of Interest

The authors declare no conflict of interest.

## Author Contributions

P.S. and D.H. contributed equally to this work. All authors have given approval to the final version of the manuscript.

## Supporting information

Supporting Information

## Data Availability

The data that support the findings of this study are available from the corresponding author upon reasonable request.
