# Supplementary material for: Anti‐Quenching NIR‐II Excitation Phenylboronic Acid Modified Conjugated Polyelectrolyte for Intracellular Peroxynitrite‐Enhanced Chemo–Photothermal Therapy
Source: Adv Sci (Weinh). 2024 Jun 17;11(30):2309446. doi: 10.1002/advs.202309446 (PMC11321672; doi:10.1002/advs.202309446)
Supplement: Supplementary file 1 — Supporting Information [file ADVS-11-2309446-s001.docx]

**Supporting Information**

Anti-Quenching NIR-II Excitation Phenylboronic Acid Modified Conjugated Polyelectrolyte for Intracellular Peroxynitrite-Enhanced Chemo-Photothermal Therapy

Pengfei Sun^1,a^, Danni Hu^1,a^, Pengfei Chen^1^, Xuanzong Wang^2^, Qingming Shen^1^, Shangyu Chen^1,*^, Daifeng Li^2,*^, and Quli Fan^1,*^

**Experimental section**

**Materials.** 4,8-Bis(5-bromo-4-(2-octyldodecy)thiophen-2-yl)-benzo[1,2-*c*;4,5-*c*']bis[1,2,5]thiadaole) (BBTD, 98%), 1,6-bis (5-trimethylbenzoyl) thiophene-2-yl) hexane (C6) were purchased from SunaTech Inc and (4,4- bis (6- bromohexyl) -4H- cyclopentadieno [2,1-b:3,4-b'] dithiene -2,6- diyl) bis (trimethylstannane) (CPDT, 95%) were purchased from Hangzhou Order Scientific & Technology Co., Ltd. 1,2-Dimyristoyl-sn-glycero-3-phosphocholine (DMPC, 99%) were purchased from *J&K* Scientific Ltd and DSPE-PEG_2000_-cRGD were purchased from Xi'an ruixi Biological Technology Co., Ltd. Tris(dibenzylideneacetone)dipalladium, tris(2-methylphenyl)phosphine were purchased from commercial sources (such as J&K Scientific Ltd., and Sigma-Aldrich). 3-Bromomethylphenylboronic acid, Dimethylamine, and Extra Dry Tetrahydrofuran were purchased from J&K Scientific Ltd. Unless indicated otherwise, all synthetic procedures were performed in an anhydrous and oxygenfree environment, and all reagents were received from commercial sources. These regents were used without further purification, except toluene which was dried and distilled with N2 before use. McCoy’s 5A, SKOV3/DDP cells, Annexin V-FITC/propidium iodide (PI) cell apoptosis kit, JC-1 (5,5’,6,6’-tetrachloro-1,1’,3,3’ tetraethylbenzimidazolcarbocyanine iodide) and Methylthiazole diphenyltetrazolium bromide (MTT) were obtained from KeyGen Biotech. Co., Ltd (Nanjing, China). Fetal bovine serum (FBS, Gibco, U.S.) was obtained from Gene Tech Co. (Shanghai, China). ATP Assay Kit, DNA Damage Assay Kit by γ-H2AX Immunofluorescence, Dihydroethidium, 3-Amino,4-aminomethyl-2',7'-difluorescein, diacetate (DAF-FM DA), Griess Reagent was obtained from Beyotime Institute of Biotechnology. BBoxiProbe O71 was obtained from Shanghai BestBio Biotechnology. ThiolTrace Violet 500 was obtained from NanJing Ningyang Biotech Co.,Ltd. DETA NONOate was obtained from Glpbio Technology Inc (Montclair, CA, USA). 5-Isothiocyanato fluorescein (FITC) and cis-Diammineplatinum dichloride (CDDP) was purchased from Macklin. HE staining, Hematoxylin dye, DAB color development kit, Fluorescein (FITC) Tunel Cell Apoptosis Detection Kit, Tissue autofluorescence quencher, Bovine serum albumin (BSA) was purchased from Servicebio.

**Characterization.** All samples were characterized using nuclear magnetic resonance (NMR) spectroscopy with a Bruker Ultra Shield Plus 400 MHz (^1^H) spectrometer. Tetramethylsilane (TMS) was selected as the internal standard and used at 298 K with CDCl_3_ or CD₃OD as the solvent for measurement. Gel permeation chromatography (GPC) was performed on Shim-pack GPC-80 X columns with THF as the eluent to determine the number-average (*M*_n_) molecular weight and polydispersity (PDI) of polymers. The morphology of nanoparticles was determined by transmission electron microscope (HT7700, TEM) under 100 KV acceleration voltage. Dynamic light scattering (DLS) analysis using a commercial laser light scattering spectrometer (ALV-7004; ALV, Langan, Germany) is equipped with a multi-τ digital time correlator and a He-Ne laser (at λ = 632.8 nm). The ⟨*D*_h_⟩ data was extracted by CONTIN analysis. All samples we used for testing were optically cleared with a Millipore filter (0.45 μm). Tests were performed at room temperature at a scattering Angle of 90°. Scanning transmission electron microscopy-energy dispersive X-ray (STEM-EDX) element mapping was performed on JEOL 2100F with ultra-high resolution (UHR) configuration (accelerating voltage: 200 kV). NIR-II fluorescence spectra were measured using an NIR-II spectrophotometer (Fluorolog 3, Horiba). NIR InGaAs was selected as the detector, with an excitation wavelength of 1064 nm obtained from a diode laser operating at 25.0 ± 0.5 °C. After the raw emission data were collected, the fluorescence signal was further confirmed and corrected for the sensitivity of InGaAs detector profile and output through the T1c channel. The laser was purchased from Changchun New Industries Optoelectronics Technology Co., Ltd. The *in vitro* and *in vivo* NIR-II FI experiments were conducted on an NIR-II imaging system (Wuhan Grand-imaging Technology Co., Ltd) with 1064 nm LP and 1200 LP filters and two types of lenses (50 or 100 mm) under the 808 nm or 1064 nm laser irradiation. A 640 × 512 pixel two-dimensional InGaAs array from Princeton Instruments in NIR-II fluorescence windows was equipped in this NIR-II FI system. All photothermal tests were detected using a Fotric 225 instrument (IR thermal camera, ± 2 °C) purchased from Fotric. (Shanghai, China). The 3-(4,5-dimethylthiazol-2-yl)-2,5-diphenyltetrazolium bromide (MTT) assay was carried out on A PowerWave XS/XS2 microplate spectrophotometer (BioTek, Winooski, VT). The flow cytometry experiments were performed using a Flow Sight Imaging Flow Cytometer (Merck Millipore, Darmstadt, Germany). All *in vitro*/*in vivo* mice photoacoustic experiments were performed using the NIR-I & NIR-II small animal whole body 3D photoacoustic imaging system, model LOIS-3D/LOIS-3D Plus, manufactured by TomoWave Laboratories, USA.

**Synthesis of PCP.** 4,8-bis (5-bromo-4 - (2-octyldodecyl) thiophene-2-yl) - benzo [1,2-c: 4,5-c '] bis [1,2,5] thiadiazole (BBTD) (193.92 mg, 0.18 mmol), (4,4- bis (6- bromohexyl) -4H- cyclopentadieno [2,1-b:3,4-b'] dithiene -2,6- diyl) bis (trimethylstannane) (CPDT) (149.4 mg, 0.18 mmol), Pd2(dba)3 (2.0 mg, 0.0064 mmol), P(o-tol)3 (4.0 mg, 0.00436 mmol) and anhydrous toluene (2.5 mL) were added into a 10 mL polymerization tube to keep the whole system in an anhydrous and oxygen free environment. Then, it was placed in an oil bath and the temperature was maintained at 100 °C for reaction. When the color of the reaction solution changed from dark green to red brown, the reaction ended. Cool it to room temperature, extract the reaction solution, settle with methanol, then filter, and finally obtain black solid as the final PCP product. Yield: 228.0 mg.

**Synthesis of PNC12.** 4,8-bis (5-bromo-4- (2-octyldodecyl) thiophene-2-yl) - benzo [1,2-c: 4,5-c '] bis [1,2,5] thiadiazole (BBTD) (193.92 mg, 0.18 mmol), 1,6-bis (5-trimethylbenzoyl) thiophene-2-yl) hexane (C6) (34.56 mg, 0.06 mmol), (4,4- bis (6- bromohexyl) -4H- cyclopentadieno [2,1-b:3,4-b'] dithiene -2,6- diyl) bis (trimethylstannane) (CPDT) (99.60 mg, 0.12 mmol), Pd2(dba)3 (2.0 mg, 0.0064 mmol), P(o-tol)3 (4.0 mg, 0.00436 mmol) and anhydrous toluene (2.5 mL) were added into a 10 mL polymerization tube to keep the whole system in an anhydrous and oxygen free environment. Then, it was placed in an oil bath and the temperature was maintained at 100 °C for reaction. When the color of the reaction solution changed from dark green to red brown, the reaction ended. Cool it to room temperature, extract the reaction solution, settle with methanol, then filter, and finally obtain black solid as the final PNC12 product. Yield: 241.0 mg.

**Synthesis of PNC11.** 4,8-bis (5-bromo-4 - (2-octyldodecyl) thiophene-2-yl) - benzo [1,2-c: 4,5-c '] bis [1,2,5] thiadiazole (BBTD) (193.92 mg, 0.18 mmol), 1,6-bis (5-trimethylbenzoyl) thiophene-2-yl) hexane (C6) (51.84 mg, 0.09 mmol), (4,4- bis (6- bromohexyl) -4H- cyclopentadieno [2,1-b:3,4-b'] dithiene -2,6- diyl) bis (trimethylstannane) (CPDT) (74.70 mg, 0.09 mmol), Pd2(dba)3 (2.0 mg, 0.0064 mmol), P(o-tol)3 (4.0 mg, 0.00436 mmol) and anhydrous toluene (2.5 mL) were added into a 10 mL polymerization tube to keep the whole system in an anhydrous and oxygen free environment. Then, it was placed in an oil bath and the temperature was maintained at 100 °C for reaction. When the color of the reaction solution changed from dark green to brown, the reaction ended. Cool it to room temperature, extract the reaction solution, settle with methanol, then filter, and finally obtain black solid as the final PNC11 product. Yield: 237.0 mg.

**Synthesis of PNC21.** 4,8-bis (5-bromo-4 - (2-octyldodecyl) thiophene-2-yl) - benzo [1,2-c: 4,5-c '] bis [1,2,5] thiadiazole (BBTD) (193.92 mg, 0.18 mmol), 1,6-bis (5-trimethylbenzoyl) thiophene-2-yl) hexane (C6) (69.12 mg, 0.12 mmol), (4,4- bis (6- bromohexyl) -4H- cyclopentadieno [2,1-b:3,4-b'] dithiene -2,6- diyl) bis (trimethylstannane) (CPDT) (49.8 mg, 0.06 mmol), Pd2(dba)3 (2.0 mg, 0.0064 mmol), P(o-tol)3 (4.0 mg, 0.00436 mmol) and anhydrous toluene (2.5 mL) were added into a 10 mL polymerization tube to keep the whole system in an anhydrous and oxygen free environment. Then, it was placed in an oil bath and the temperature was maintained at 100 °C for reaction. When the color of the reaction solution changed from dark green to brown, the reaction ended. Cool it to room temperature, extract the reaction solution, settle with methanol, then filter, and finally obtain black solid as the final PNC21 product. Yield: 237.0 mg.

**Synthesis of PCPBA, PNC12BA, PNC11BA, PNC21BA.** Take 100.0 mg of the corresponding products in the previous step, put them into a 50 mL round-bottomed flask, add 10 mL of ultra-dry tetrahydrofuran into the flask, and slowly add 3 mL of dimethylamine solution under the condition of external ice bath after three times of vacuumizing and blowing nitrogen. After reacting in ice bath for 1 hour, the reaction bottle was put into an oil bath pot at 50 ℃, and after 3 days of reaction, the solvent was removed by rotary evaporation. Add 10 mL of ultra-dry tetrahydrofuran and 20.0 mg of 3-Bromomethylphenylboronic acid into the reaction bottle. React for three days under the condition of removing oxygen and water. The product will eventually precipitate in the solvent.

**Preparation of PCP NPs, PNC12 NPs, PNC11 NPs, PNC21 NPs.** First, 1.0 mg PCP, PNC12, PNC11 or PNC21 was dissolved in 1 mL THF solution, and 10.0 mg DMPC was dissolved in 0.1 mL DCM solution. The two solvents were mixed together, quickly injected into a glass bottle filled with 10 mL of water, and ultrasonicated for 3 min.

**Preparation of PCPBA NPs, PNC12BA NPs, PNC11BA NPs, PNC21BA NPs.** First, 1.0 mg PCPBA, PNC12BA, PNC11BA or PNC21BA was dissolved in 1 mL of a mixed solvent of THF and DCM, and 10.0 mg DMPC was dissolved in 0.1 mL DCM solution. The two solvents were mixed together, quickly injected into a glass bottle filled with 10 mL of water, and ultrasonicated for 3 min.

**Preparation of PBT/NO.** First, 1.0 mg PNC11BA was dissolved in 1 mL of a mixed solvent of THF and DCM, and 10.0 mg DMPC was dissolved in 0.1 mL DCM solution. The two solvents are mixed together, and a certain amount of DCM solution is evaporated under the high temperature evaporation in a water bath pot. Then, 1.0 mg of DETA NONOate and 1.0 mg of DSPE-PEG_2000_-cRGD were weighed and dissolved in 10 mL of water. Finally, the organic solvent was quickly pumped into a glass bottle filled with water, and ultrasonic was performed for 1 min.

**Preparation of PBT/Pt.** First, 1.0 mg PNC11BA was dissolved in 1 mL of a mixed solvent of THF and DCM, and 10.0 mg DMPC was dissolved in 0.1 mL DCM solution. The two solvents are mixed together, and a certain amount of DCM solution is evaporated under the high temperature evaporation in a water bath pot. Then, 1.0 mg of DSPE-PEG_2000_-cRGD was dissolved in 10 mL of water, and 1.0 mg of Cis-Diaminodichloroplatinum (CDDP) was dissolved in 0.5 mL of THF. Finally, the organic solvent was quickly pumped into a glass bottle filled with water, and ultrasonic was performed for 1 min.

**Preparation of PBT/NO/Pt.** First, 1.0 mg PNC11BA was dissolved in 1 mL of a mixed solvent of THF and DCM, and 10.0 mg DMPC was dissolved in 0.1 mL DCM solution. The two solvents are mixed together, and a certain amount of DCM solution is evaporated under the high temperature evaporation in a water bath pot. Then, 1.0 mg of DETA NONOate and 1.0 mg of DSPE-PEG_2000_-cRGD were dissolved in 10 mL of water, and 1.0 mg of Cis-Diaminodichloroplatinum (CDDP) was dissolved in 0.5 mL of THF. Finally, the organic solvent was quickly pumped into a glass bottle filled with water, and ultrasonic was performed for 1 min.

***In vitro* photothermal effect and photothermal conversion efficiency.** To evaluate the photothermal effect of PBT/NO/Pt, 200 μL of two nanoparticle solutions with concentrations of 0.1, 0.08 and 0.06 mg mL^-1^ were successively irradiated with 1064 nm laser (1.0 W cm^-2^, 6 min). The temperature changes of the PBT/NO/Pt solutions were performed with an IR thermal camera, and these data were recorded every 30 s.

To study the photothermal conversion behavior of PBT/NO/Pt, a thermal imaging camera (Fotric 225, Fotric Precision Instruments, USA, ± 2 °C) was used to perform the thermal imaging of NPs in an aqueous solution. First, PBT/NO/Pt (0.1 mg mL^-1^) was prepared, and 200 μL of each nanoparticle was added into a 200 μL centrifuge tube. Second, the temperature changes of the fixed concentration of PBT/NO/Pt (0.1 mg mL^-1^) was irradiated with a 1064 nm laser (1.0 W cm^-2^, 6 min), and then the laser was shut off. Finally, we can obtain a temperature increase and drop curve.

The photothermal conversion efficiency (ŋ) was calculated using equations (1) and (2) expressed below. The photothermal conversion efficiency of the PBT/NO/Pt was determined to be through the collected data and equation.

ŋ = [hS(T_max_-T_suur_)-Q_dis_]/[I(1-10^-A1064^)] (1)

τ_s_ =m_D_C_D_/hS (2)

The parameters S, h, T_max_, T_surr_, Q_dis_, I and A_1064_ are the container’s surface area, heat-transfer coefficient, maximum laser-trigger temperature, indoor temperature, heat dissipation caused by the light absorbing of quartz cuvette, intensity of laser (1.0 W cm^-2^) and absorbance of PBT/NO/Pt at 1064 nm, respectively.

Parameter τ_s_ is the time constant of the sample system. The parameters m_D_ and C_D_ are the mass and heat capacity of the solvent, respectively.

***In vitro* NO release.** We first prepared NaNO_2_ aqueous solutions with different concentrations and added them into 96-well plates. Each test hole was added with 50 μL Griess Ⅰ and 50 μL Griess Ⅱ respectively. Make a standard curve with the absorbance value detected by enzyme-labeled instrument at 540 nm as shown in Figure S33. 0.1 mg mL^−1^ of PBT/NO/Pt was prepared, put in an ultrafiltration tube, concentrated by a centrifuge, and finally prepared into a 2.0 mg mL^−1^ aqueous solution. Add 200 μL of PBT/NO/Pt to buffer solutions with different pH values, and add 50 μL Griess Ⅰ and 50 μL Griess Ⅱ. The absorption curve of the solution was detected by UV, and finally the release value of DETA NONOate was calculated from the standard curve and the absorption at 540 nm.

***In vitro* Pt release.** Standard solutions of Pt were prepared, different concentrations of Pt solution were diluted with dilute nitric acid, and the standard curve was measured by ICP-MS, as shown in Figure S34. 0.1 mg mL^−1^ of PBT/NO/Pt was prepared, put in an ultrafiltration tube, concentrated by a centrifuge, and finally prepared into a 0.2 mg mL^−1^ aqueous solution. Add 100 μL of PBT/NO/Pt to 4.9 mL buffer solutions with different pH values. The content of Pt at different time points was measured by ICP-MS.

***In vitro* Cellular Uptake.** The SKOV3/DDP cells were used for the assessment of cellular uptake. First, cells, seeded 4 × 10^4^ per well, were incubated in McCoy’s 5A in a 6-well plate at 37 °C and 5% CO_2_ condition for 24 h. The treated cells were further incubated at 37 °C and 5% CO_2_ for 0 h, 1.5 h and 3 h, respectively. FITC was used as a fluorescent marker to determine whether the material was uptaken by cells. Afterward, the upper supernatant was sucked out and 1 mL of PBS was added to clean twice and remove dead cells. Subsequently, PBS was removed and 500 μL trypsin digestion solution without EDTA was added, and the cells were dissolved in an incubator at 37 °C and 5% CO_2_ for 420 s. Later 1 mL of McCoy’s 5A was added and the cells were transferred into a 2 mL centrifuge tube and centrifuged for 3 min. The supernatant was then removed, and 100 μL of PBS was added to the centrifuge tube. Finally, the uptake of the intracellular material was measured by flow cytometry.

**Cellular internalization**. To track internalization of PBT/NO/Pt in tumor cells, SKOV3/DDP cells (1 × 10^4^) were grown on slices in a 6-well culture plate at 37 °C and 5% CO_2_ condition for 24 h, and then incubated with FITC/PBT/NO/Pt (0.1 mg mL^-1^). FITC was used as a fluorescent marker to determine whether the material was uptaken by cells. After 1.5 h and 3 h, the cells were washed with PBS for three times, followed by costaining with DAPI (nucleus indicator, 10 min). Then, the cells were imaged by CLSM (Olympus Fluoview FV1000).

**Intracellular NO** **detection by confocal imaging and flow cytometry.** SKOV3/DDP cells (1 × 10^4^) were grown in a confocal dish at 37 °C and 5% CO_2_ condition for 24 h. After that, each dish of the confocal dish was added with a solution of PBT, PBT/Pt, PBT/NO, PBT/NO/Pt with McCoy’s 5A dilution and the plate was cultured for accessional 6 h in CO_2_ incubator. The media were removed and cells were washed three times with 1 mL PBS solution, then 1 mL DAF-FM DA diluent was added to stain cells for 20 min. Then, cells were washed three times with 1 mL PBS solution and 2 mL fresh McCoy’s 5A was added to the cell culture dish. Then, the cells were imaged with CLSM (Olympus Fluoview FV1000), respectively.

**Intracellular O_2_^•−^ detection by confocal imaging and flow cytometry.** SKOV3/DDP cells (1 × 10^4^) were grown in a confocal dish at 37 °C and 5% CO_2_ condition for 24 h. After that, each dish of the confocal dish was added with a solution of PBT, PBT/Pt, PBT/NO, PBT/NO/Pt with McCoy’s 5A dilution and the plate was cultured for accessional 6 h in CO_2_ incubator and the media were removed and cells were washed three times with 1 mL PBS solution, then 1 mL DHE diluent was added to stain cells for 30 min. Then, cells were washed three times with 1 mL PBS solution and 2 mL fresh McCoy’s 5A was added to the cell culture dish. Then, the cells were imaged with CLSM (Olympus Fluoview FV1000), respectively.

**Intracellular ONOO^−^ detection by confocal imaging and flow cytometry.** SKOV3/DDP cells (1 × 10^4^) were grown in a confocal dish at 37 °C and 5% CO_2_ condition for 24 h. After that, each dish of the confocal dish was added with a solution of PBT, PBT/Pt, PBT/NO, PBT/NO/Pt with McCoy’s 5A dilution and the plate was cultured for accessional 6 h in CO_2_ incubator and the media were removed and cells were washed three times with 1 mL PBS solution, then 1 mL BBoxiProbe O71 diluent was added to stain cells for 30 min. Then, cells were washed three times with 1 mL PBS solution and 2 mL fresh McCoy’s 5A was added to the cell culture dish. Then, the cells were imaged with CLSM (Olympus Fluoview FV1000), respectively.

***In vitro* cytotoxicity assay.** SKOV3/DDP cells were cultured with McCoy’s 5A supplemented with 10% FBS. The surrounding environment is at 37 ℃ with a humidified 5% CO_2_. Cells (1×10^4^ cells/well) were incubated in a 96-well plate for 24 h, different concentrations of PBT (100 μL) were diluted in McCoy’s 5A and added to the wells, respectively. Cells were cultured for another 24 h. After that, each well of the microliter plate was added with a solution of methylthiazole diphenyltetrazolium bromide (MTT) with McCoy’s 5A dilution and the plate was cultured for accessional 4 h in CO_2_ incubator. Finally, 100 μL DMSO was added to each well, and it was detected by Bio-tek Synergy HTX microplate spectrophotometer to determine the 490 nm absorbance of each well. The following formula was used to calculate the viability of cell growth: Viability (%) = (mean absorbance value of treatment group/mean absorbance value of control group) × 100%.

**Assessment of NIR-II photothermal/chemotherapy/peroxynitrite synergistic therapies *in vitro* using the MTT assay.** For the cell viability analysis, SKOV3/DDP cells were first cultured in 96-well plates at a density of 2×10^4^ cells per well. After 24 h, cells were transferred to a mixed medium containing different doses (0, 6.25, 12.5, 25, 50, and 100 mg μL^-1^) of materials from different groups (PBT + Laser, PBT/Pt, PBT/NO, PBT/NO/Pt + Laser) and incubated in the dark. Thereafter, selected wells from the different groups were illuminated with or without an 1064 nm laser at a power of 1.0 W cm^-2^ for 6 min. After another incubation for 24 h, 100 μL fresh McCoy’s 5A and 10 μL of methylthiazole diphenyltetrazolium bromide (MTT) solution were added to each well and incubated for another 4 h in CO_2_ incubator. Finally, 100 μL DMSO was added to each well, and it was detected by Bio-tek Synergy HTX microplate spectrophotometer to determine the 490 nm absorbance of each well.

**Assessment of NIR-II photothermal/chemotherapy/peroxynitrite synergistic therapies *in vitro* using flow cytometry.** SKOV3/DDP cells were cultured with McCoy’s 5A in 6-well plates until the cell density increased to 1×10^5^ cells mL^-1^ per well. Materials in different groups (PBT, PBT + Laser, PBT/Pt, PBT/NO, PBT/NO/Pt + Laser) were added to fresh McCoy’s 5A to obtain the mixed medium (0.1 mg mL^-1^). After the SKOV3/DDP cells had been co-incubated with this mixed medium for 4 h in the dark, the media were removed and replaced with 1 mL fresh McCoy’s 5A. Next, the selected wells from different groups were illuminated with or without a 1064 nm laser at a power of 1.0 W cm^-2^ for 6 min. After a 12 h incubation to allow cells to undergo apoptosis, the SKOV3/DDP cells that had detached from the 6-well plates were washed several times with 1 mL PBS. Subsequently, PBS was removed and 500 μL trypsin digestion solution without EDTA was added, and the cells were dissolved in an incubator at 37 °C and 5% CO_2_ for 420 s. Later 1 mL of McCoy’s 5A was added and the cells were transferred into a 2 mL centrifuge tube and centrifuged for 3 min. An Annexin V-FITC/PI staining solution was then mixed with the collected cells for 15 min, and cells were analyzed using flow cytometry.

***In vivo* NIR-II fluorescence imaging.** When the tumors reached a volume of 90-120 mm^3^, SKOV3/DDP tumor bearing mice were intravenously injected with PBT/NO/Pt (150 μL, 2.0 mg mL^-1^). The tumor bearing mice were imaged alive by anesthetizing them with isoflurane during the test time (just about 10 min) in the case of the eﬀect of respiration. The real-time *in vivo* NIR-II fluorescence imaging was performed at diﬀerent post-injection times by using an *in vivo* NIR-II fluorescence imaging system. The analysis of the signal intensity of NIR-II image was performed using the NIR-II *in vivo* imaging system software.

***In vivo* NIR-II photoacoustic imaging.** When the tumors reached a volume of 90-120 mm^3^, SKOV3/DDP tumor bearing mice were intravenously injected with PBT/NO/Pt (150 μL, 2.0 mg mL^-1^). Mice are mounted on specially designed holders, which rotate 360 degrees with sophisticated rotating motors. Four short pulsed laser beams (biorthogonal, bioblique) are emitted by a laser the tissue absorbs light energy to generate ultrasonic signal, which is received by a professionally designed 125° are transducer. The signal was collected at 360°, and the photoacoustic image was reconstructed by special 3D reconstruction software through unit transformation of DAQ data.

***In vivo* NIR-II photothermal/chemotherapy/peroxynitrite synergistic therapies.** When the tumor volume reached 90-120 mm^3^, the SKOV3/DDP tumor-bearing mice were weighed, randomly divided into different treatment groups. Tumor size was monitored every 2 days after intravenous administration of PBS, PBT, PBT/Pt, PBT/NO, or PBT/NO/Pt (150 μL, 2.0 mg mL^−1^). At 24 h postinjection, tumor sites in the PBT + Laser and PBT/NO/Pt + Laser groups were exposed to 1064 nm laser (1.0 W cm^−2^) irradiation for 6 min. After 15 days, these mice were sacrificed, and their major organs were stained with hematoxylin-eosin (H&E) staining, and their tumors were stained with hematoxylin-eosin (H&E) staining, Ki-67, γ-H_2_AX expression and TUNEL staining. The histological tumor sections were observed using an optical microscope. The weight of the tumors were also measured.

**Blood hematology and biochemistry analysis.** Collect the blood samples of PBS, PBT, PBT/Pt, PBT/NO, PBT + Laser and PBT/NO/Pt + Laser treated group from the mice (n = 3 per group) with a concentration of 830 μM (150 μL) at the day 15. First, all blood samples were centrifuged at 1200 rpm for 15 min and placed at 25 ℃ for 2.5 h until the RBCs and blood plasma separated. After repeated washes with PBS, the blood plasma was analyzed. Renal function markers (CRE & BUN) and hepatic function markers (ALT and AST) were measured, and detailed routine blood parameters including the white blood cells (WBC), RBC, hematocrit (HCT), hemoglobin (HGB), platelets (PLT), mean corpuscular HGB concentration (MCHC), mean corpuscular volume (MCV), and mean corpuscular HGB (MCH) were analyzed.


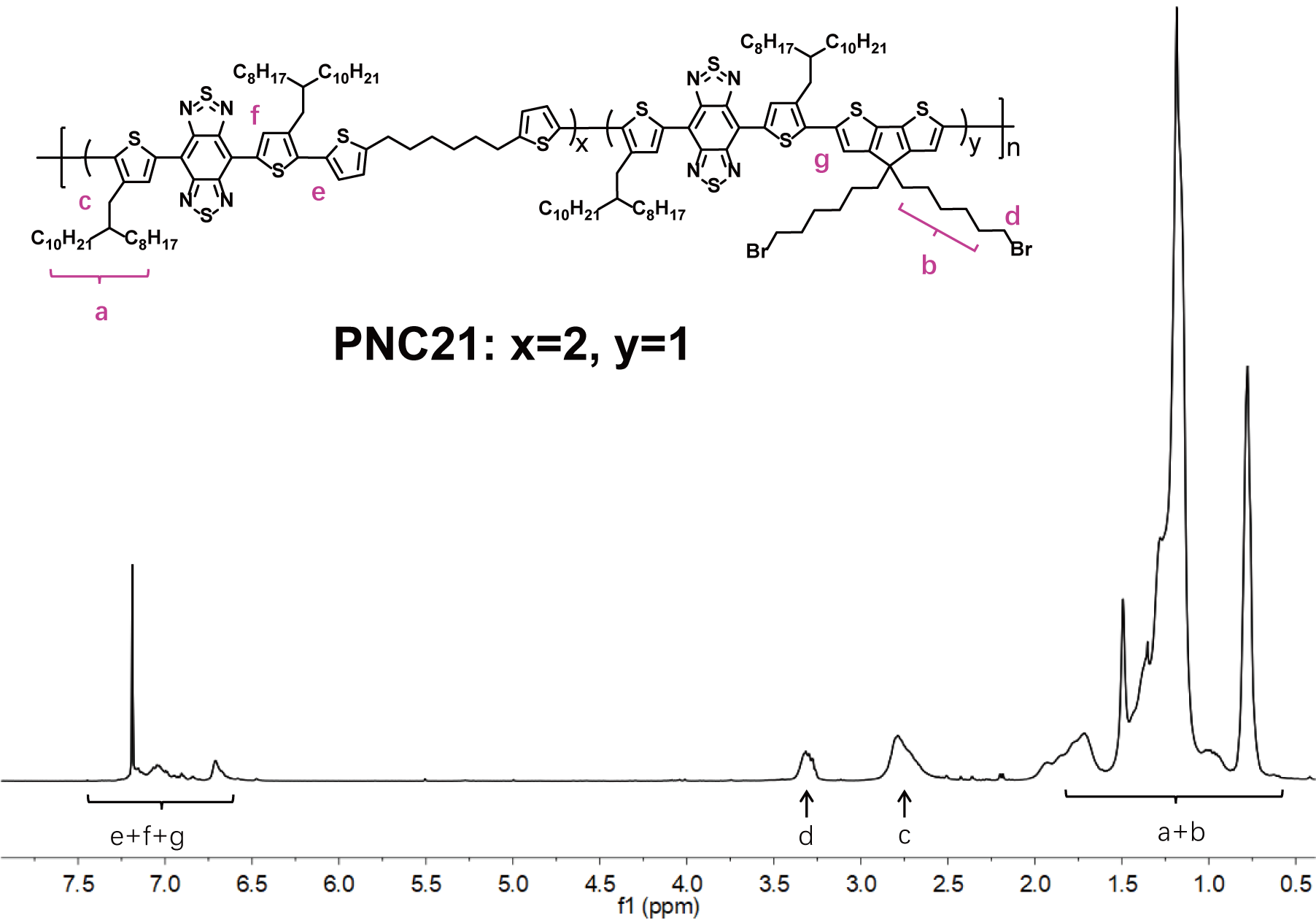


**Figure S1.** ^1^H NMR spectrum of PNC21 in CDCl_3_.


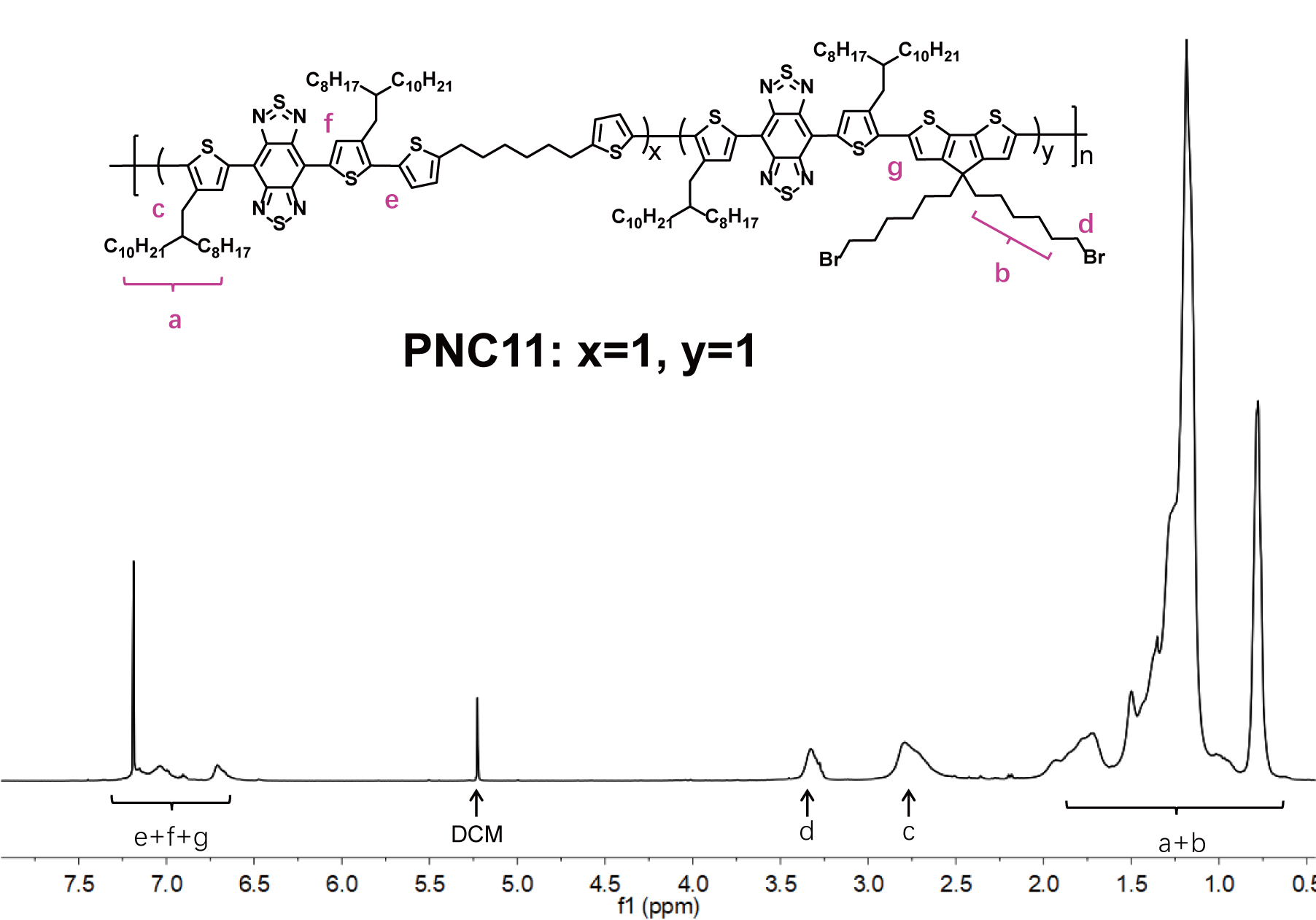


**Figure S2.** ^1^H NMR spectrum of PNC11 in CDCl_3_.


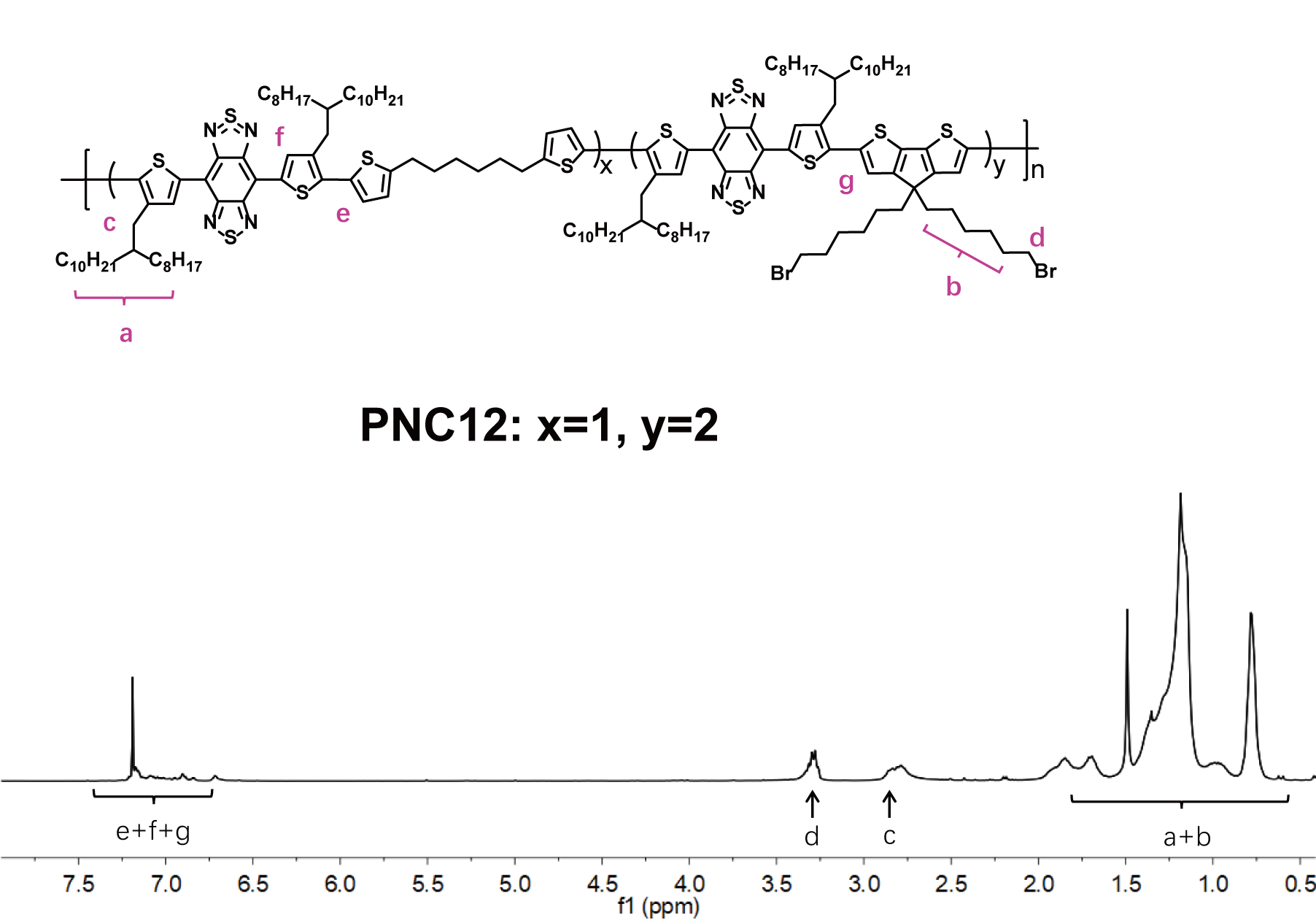


**Figure S3.** ^1^H NMR spectrum of PNC12 in CDCl_3_.

**
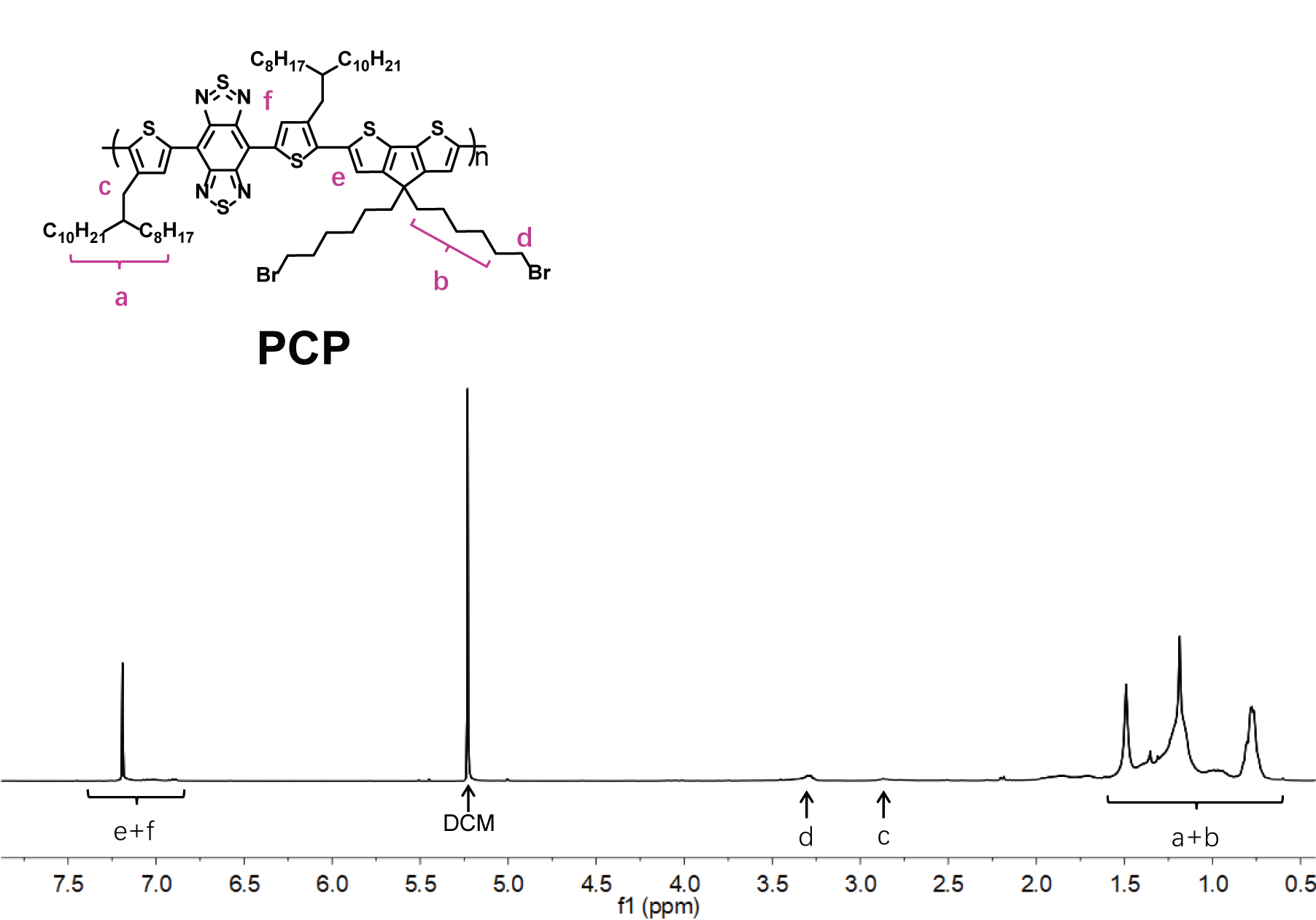
**

**Figure S4.** ^1^H NMR spectrum of PCP in CDCl_3_.


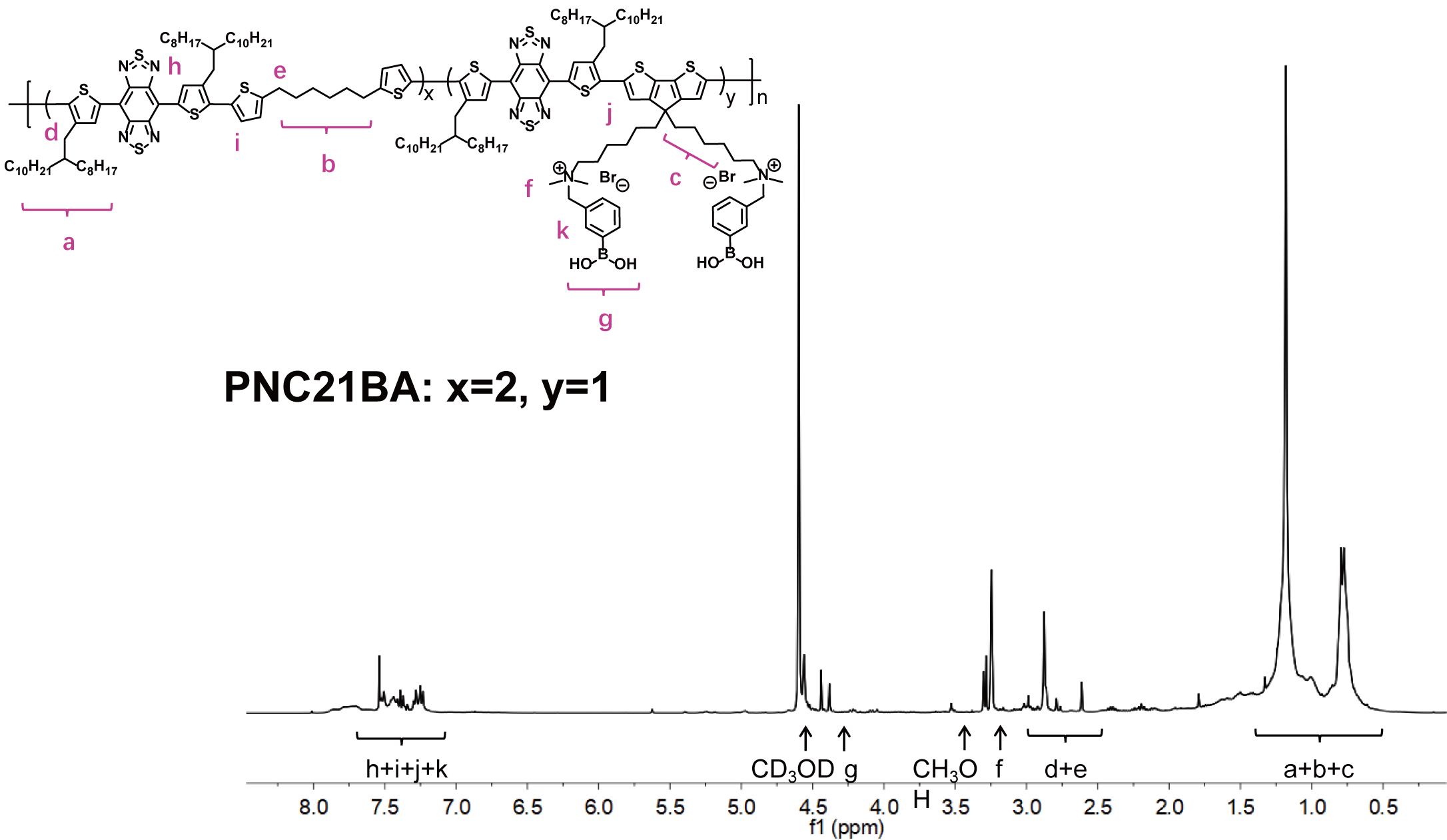


**Figure S5.** ^1^H NMR spectrum of PNC21BA in CDCl_3_ and CD₃OD (1:1).


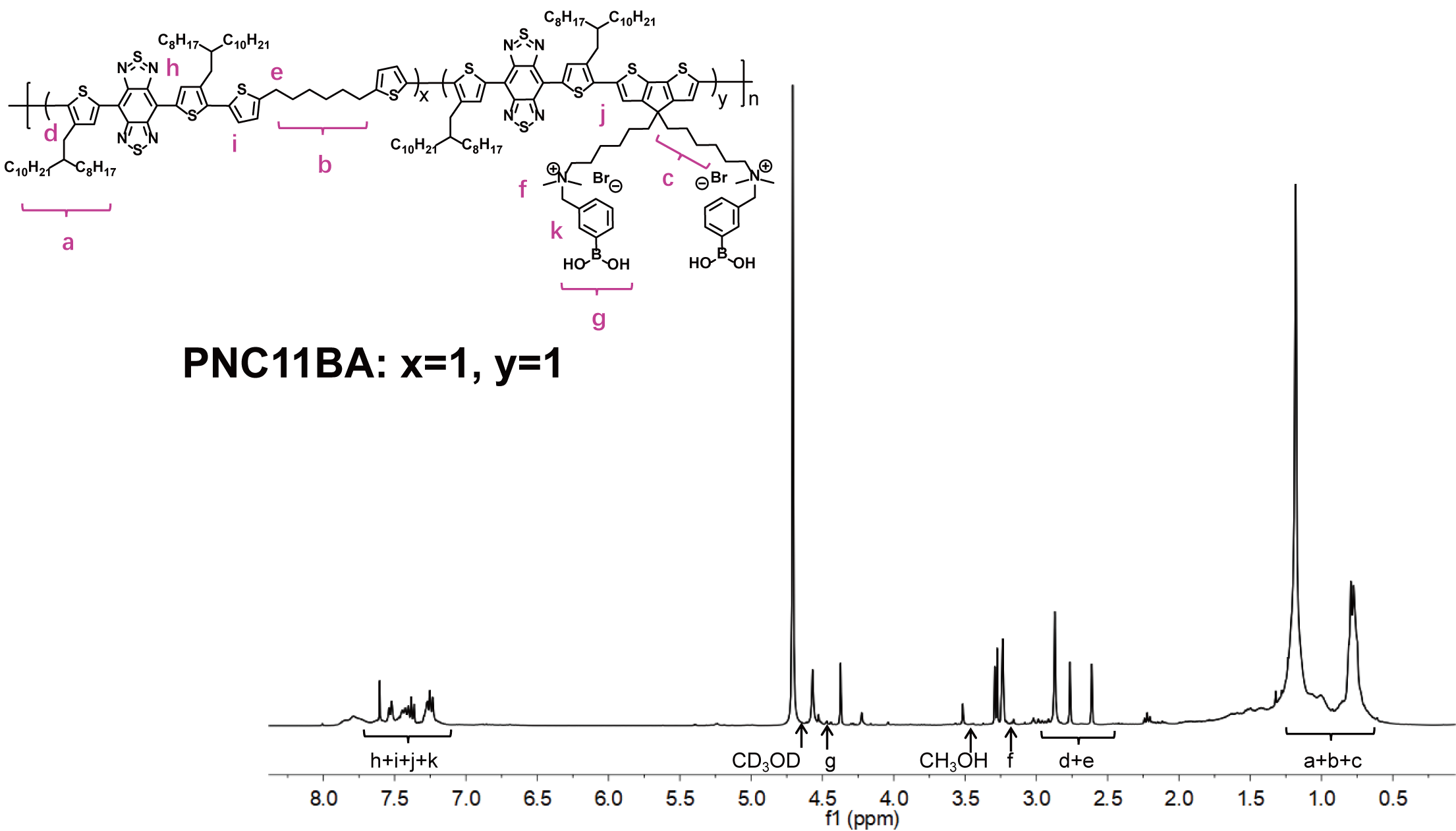


**Figure S6.** ^1^H NMR spectrum of PNC11BA in CDCl_3_ and CD₃OD (1:1).


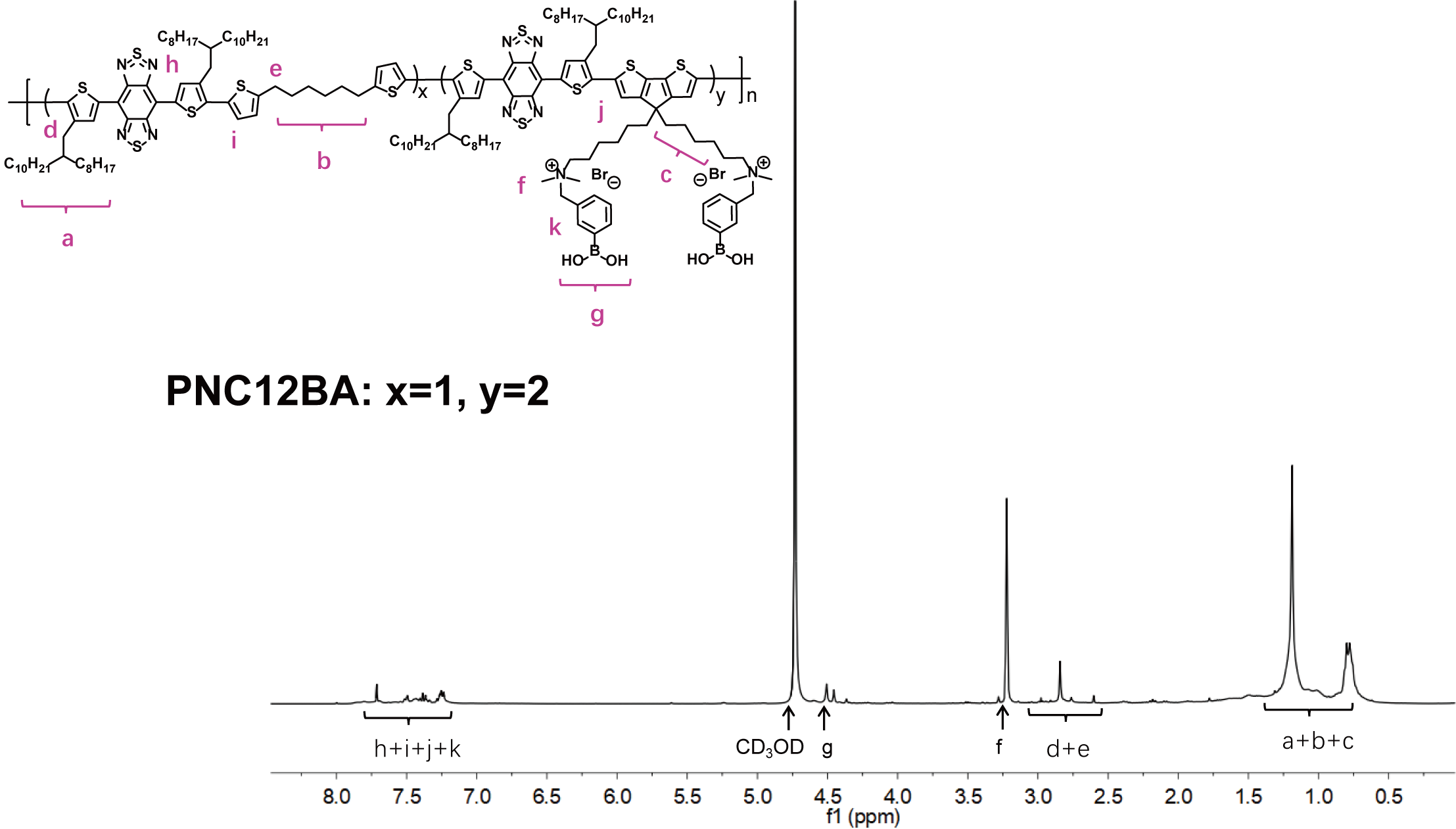


**Figure S7.** ^1^H NMR spectrum of PNC12BA in CDCl_3_ and CD₃OD (1:1).


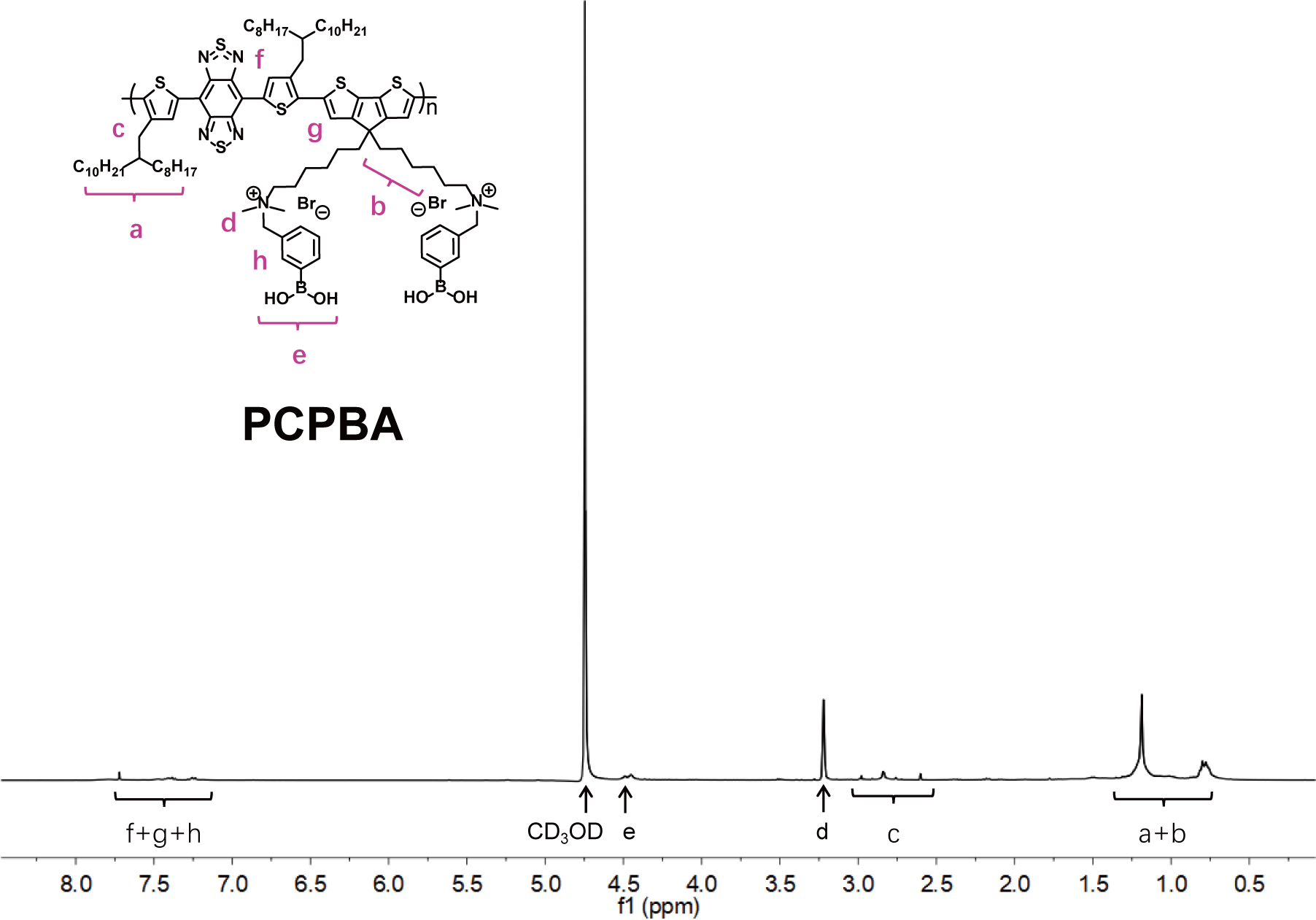


**Figure S8.** ^1^H NMR spectrum of PCPBA in CDCl_3_ and CD₃OD (1:1).

**
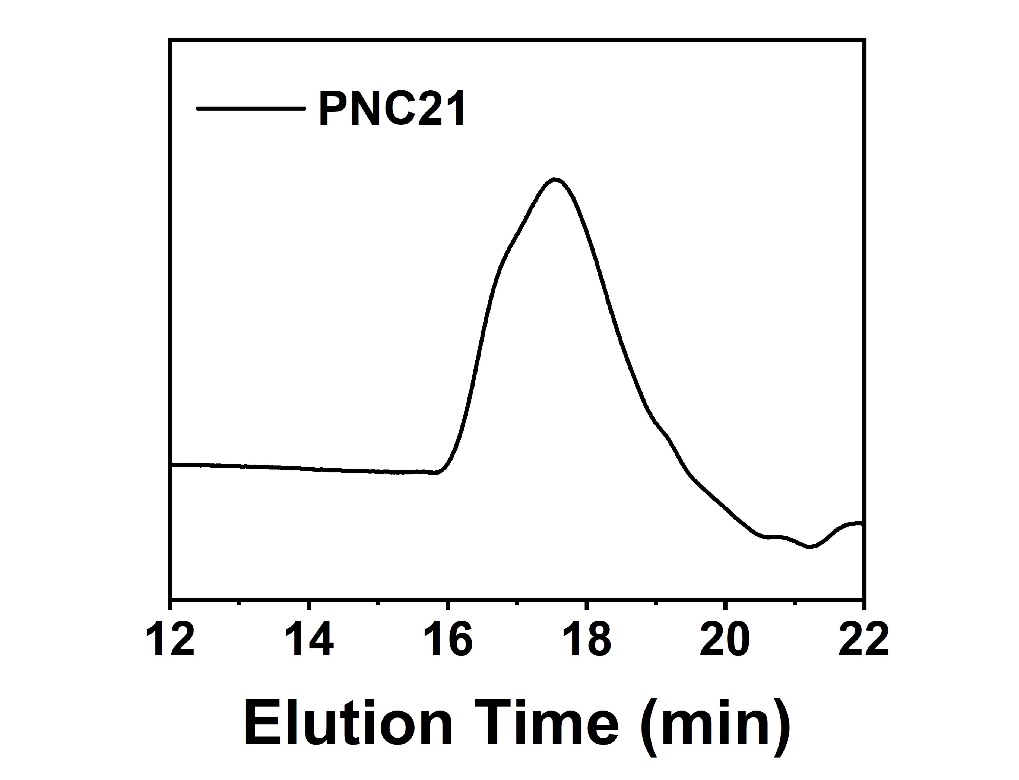
**

**Figure S9.** The GPC curve of PNC21 using THF as eluent.


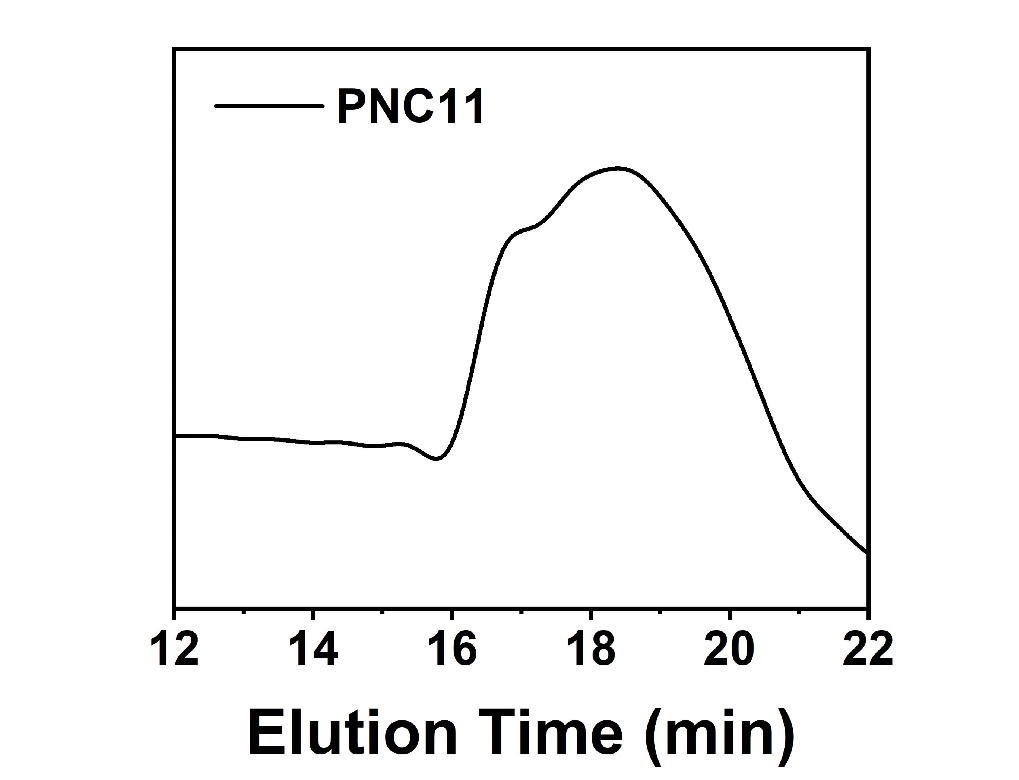


**Figure S10.** The GPC curve of PNC11 using THF as eluent.

**
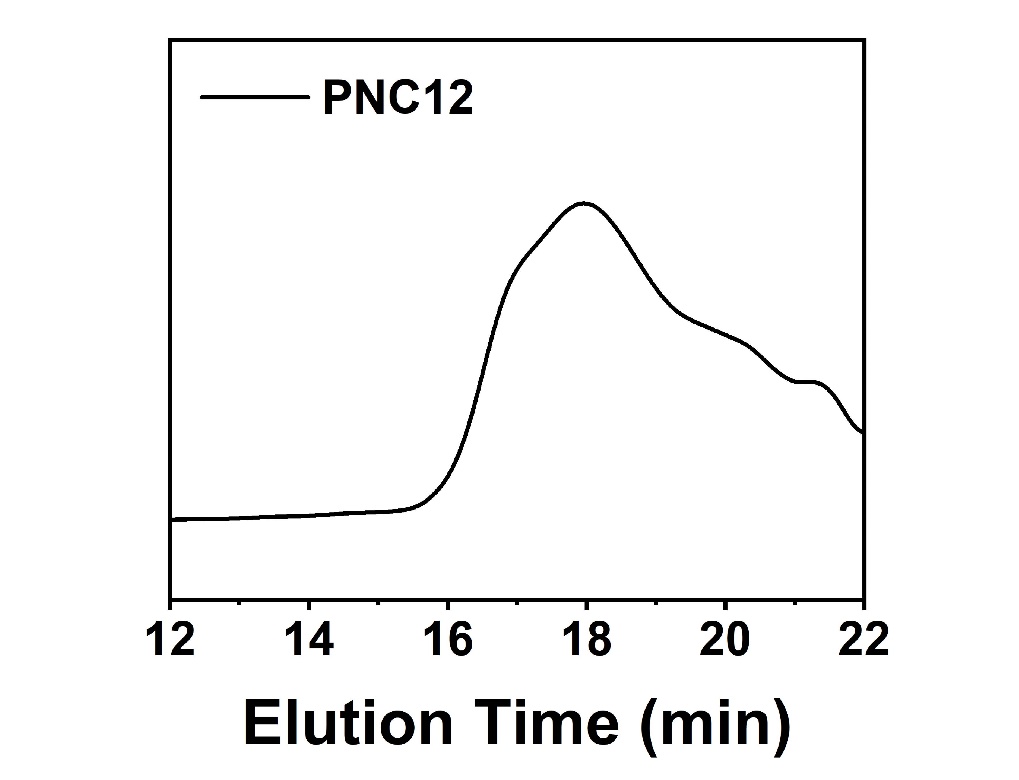
**

**Figure S11.** The GPC curve of PNC12 using THF as eluent.

**
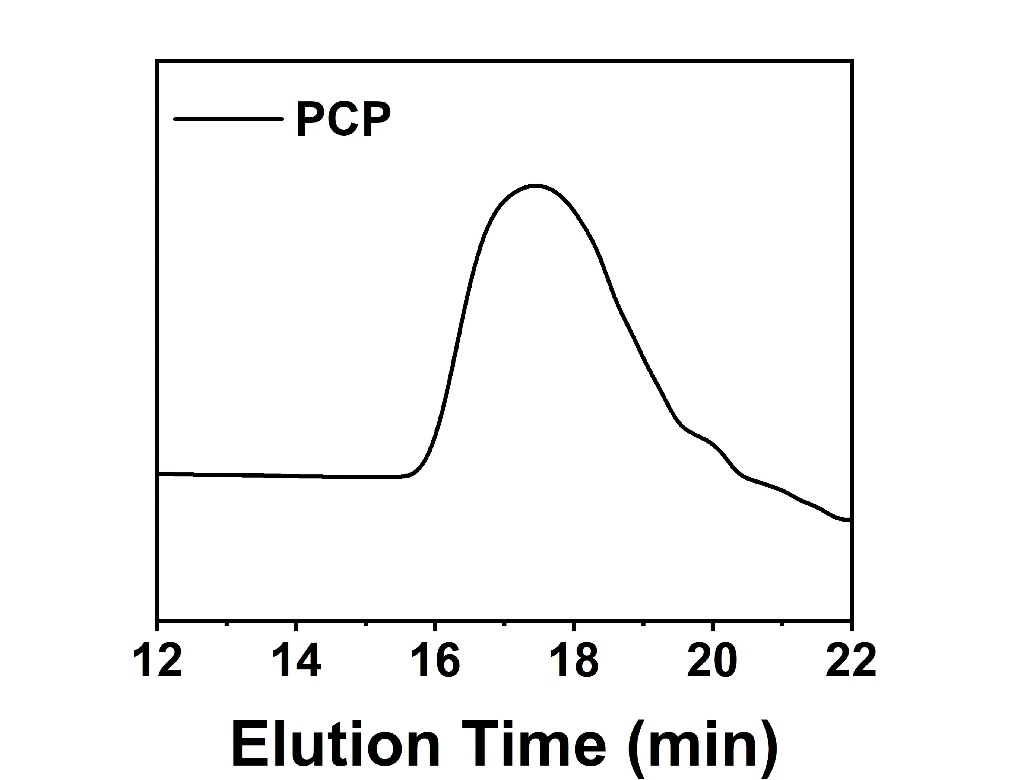
**

**Figure S12.** The GPC curve of PCP using THF as eluent.

**Table S1.** The number average molecular weights (*M*n) of four polymers.

| **Sample name** | ***M*n (g mol^-1^)** |
| --- | --- |
| PCP | 10600 |
| PNC12 | 25000 |
| PNC11 | 15200 |
| PNC21 | 13200 |


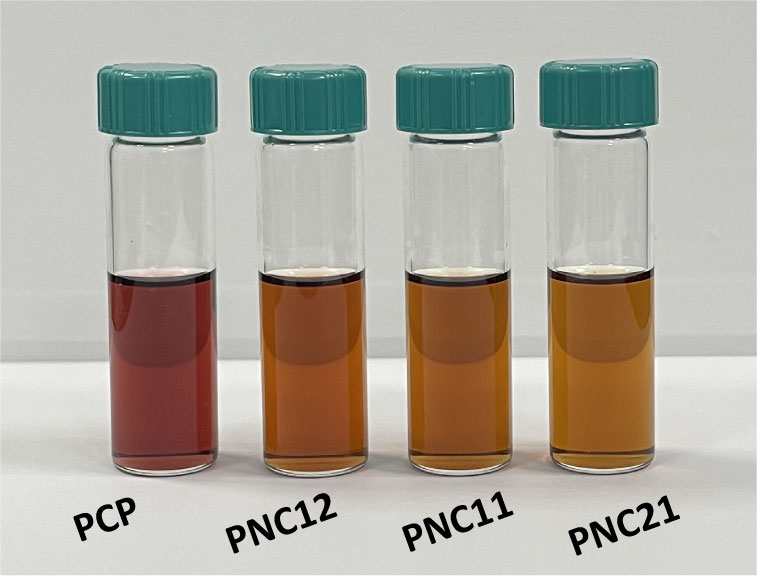


**Figure S13.** Photograph of polymers in THF solution (0.1 mg mL^-1^).


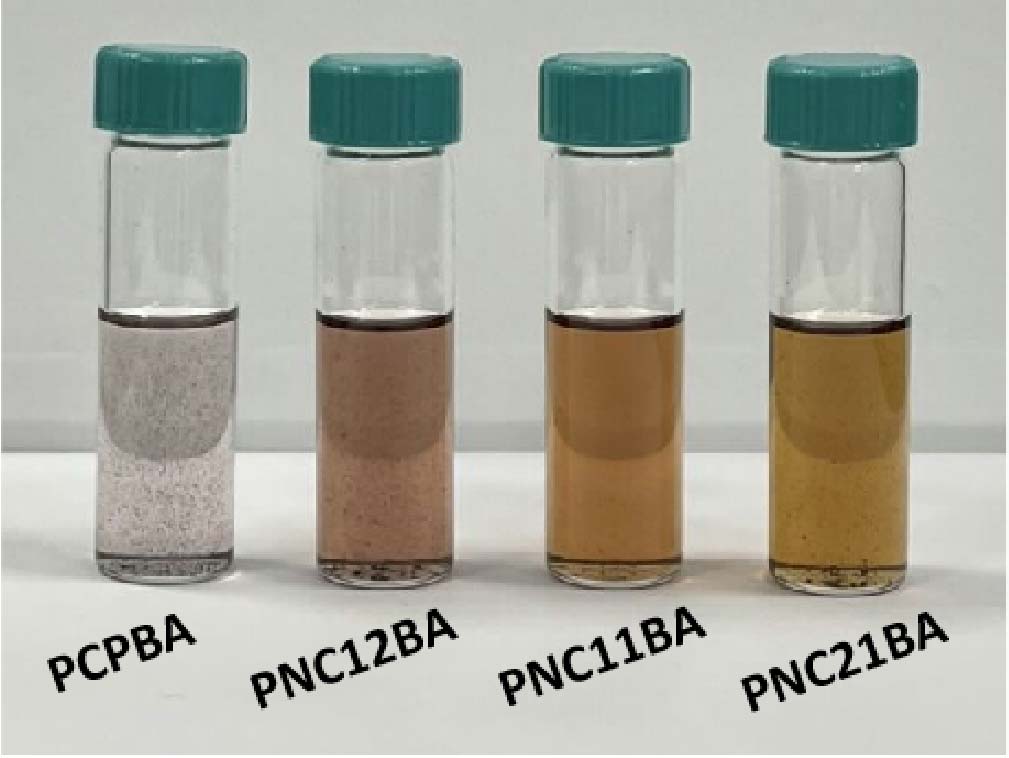


**Figure S14.** Photograph of polymers in THF solution (1.0 mg mL^-1^).


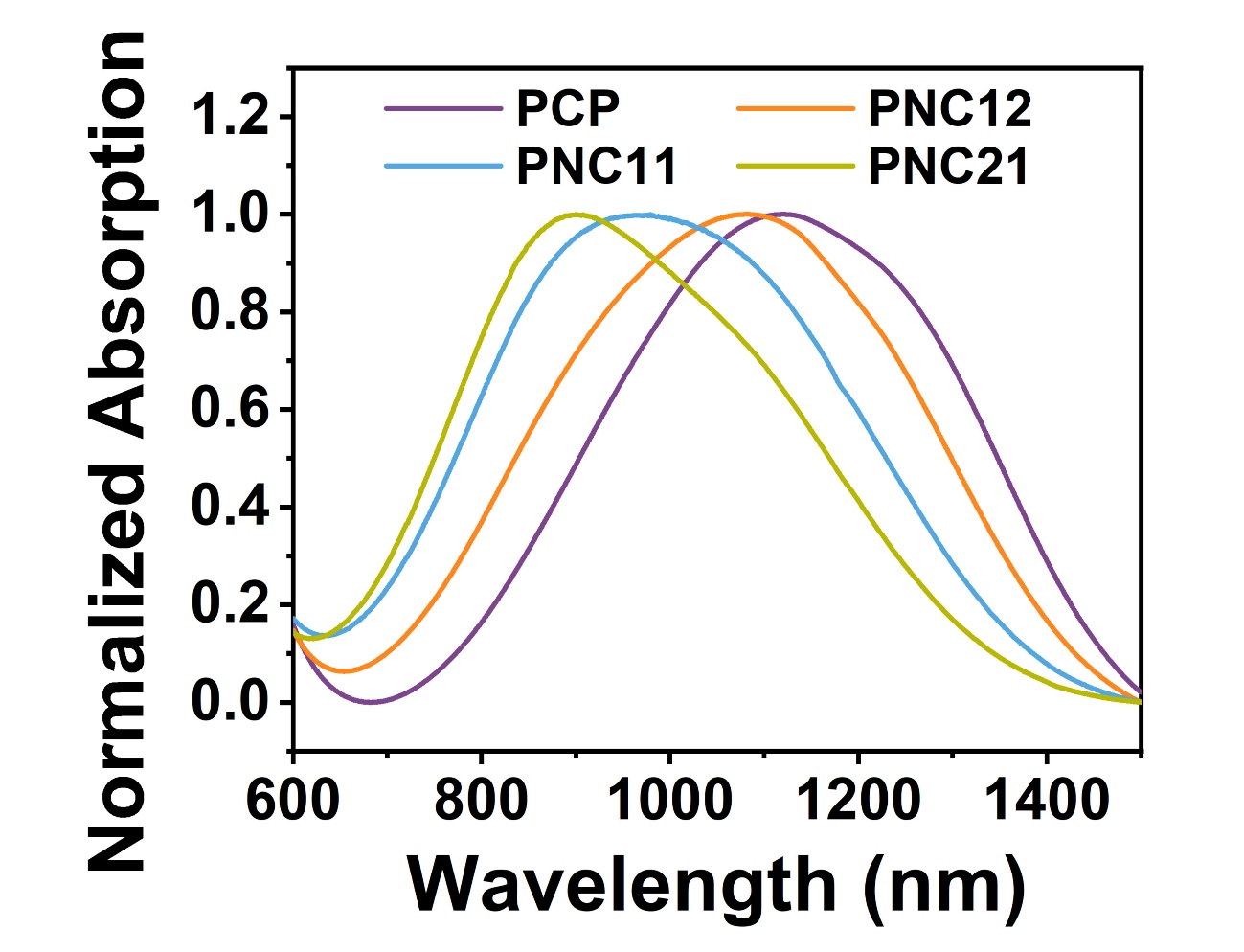


**Figure S15.** Normalized absorption spectra of PCP, PNC12, PNC11, and PNC21 in THF.


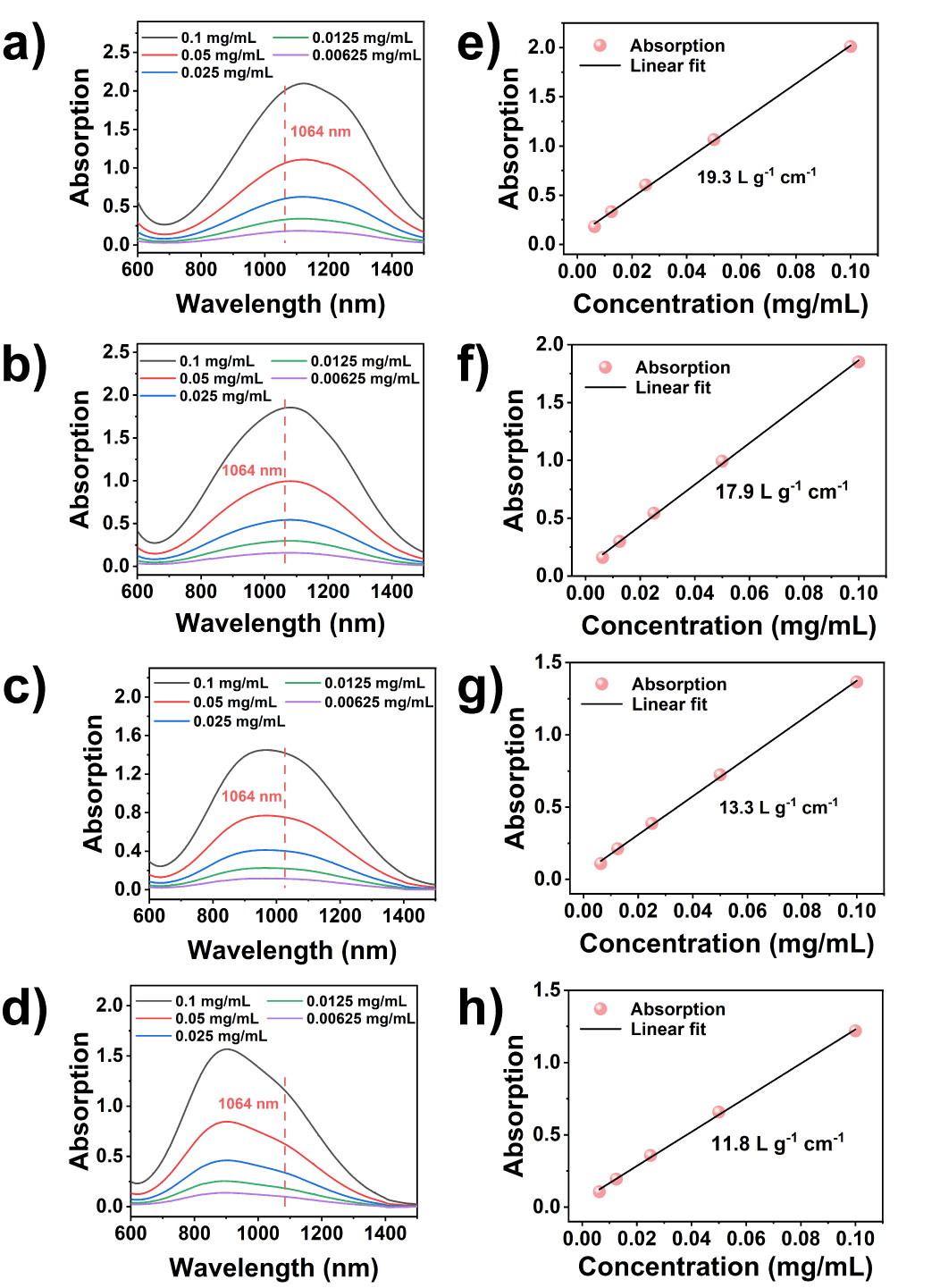


**Figure S16.** UV-vis-NIR absorption spectra of (a) PCP, (b) PNC12, (c) PNC11 and (d) PNC21 in THF at different concentrations, and the extinction coefficients (e) PCP, (f) PNC12, (g) PNC11 and (h) PNC21 at peak absorption, respectively.


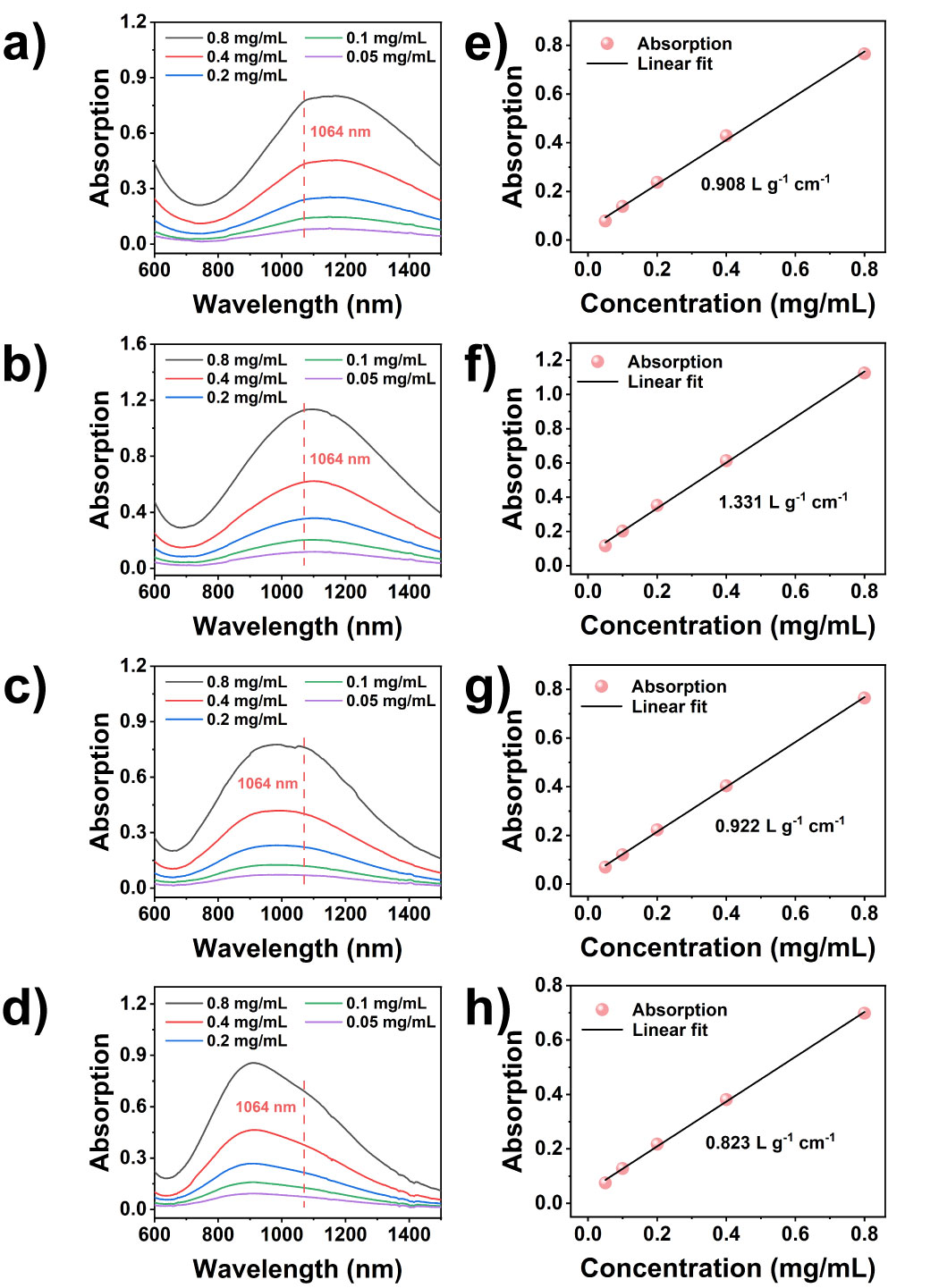


**Figure S17.** UV-vis-NIR absorption spectra of (a) PCPBA, (b) PNC12BA, (c) PNC11BA and (d) PNC21BA in THF at different concentrations, and the extinction coefficients (e) PCPBA, (f) PNC12BA, (g) PNC11BA and (h) PNC21BA at corresponding peaks of 1064 nm, respectively.


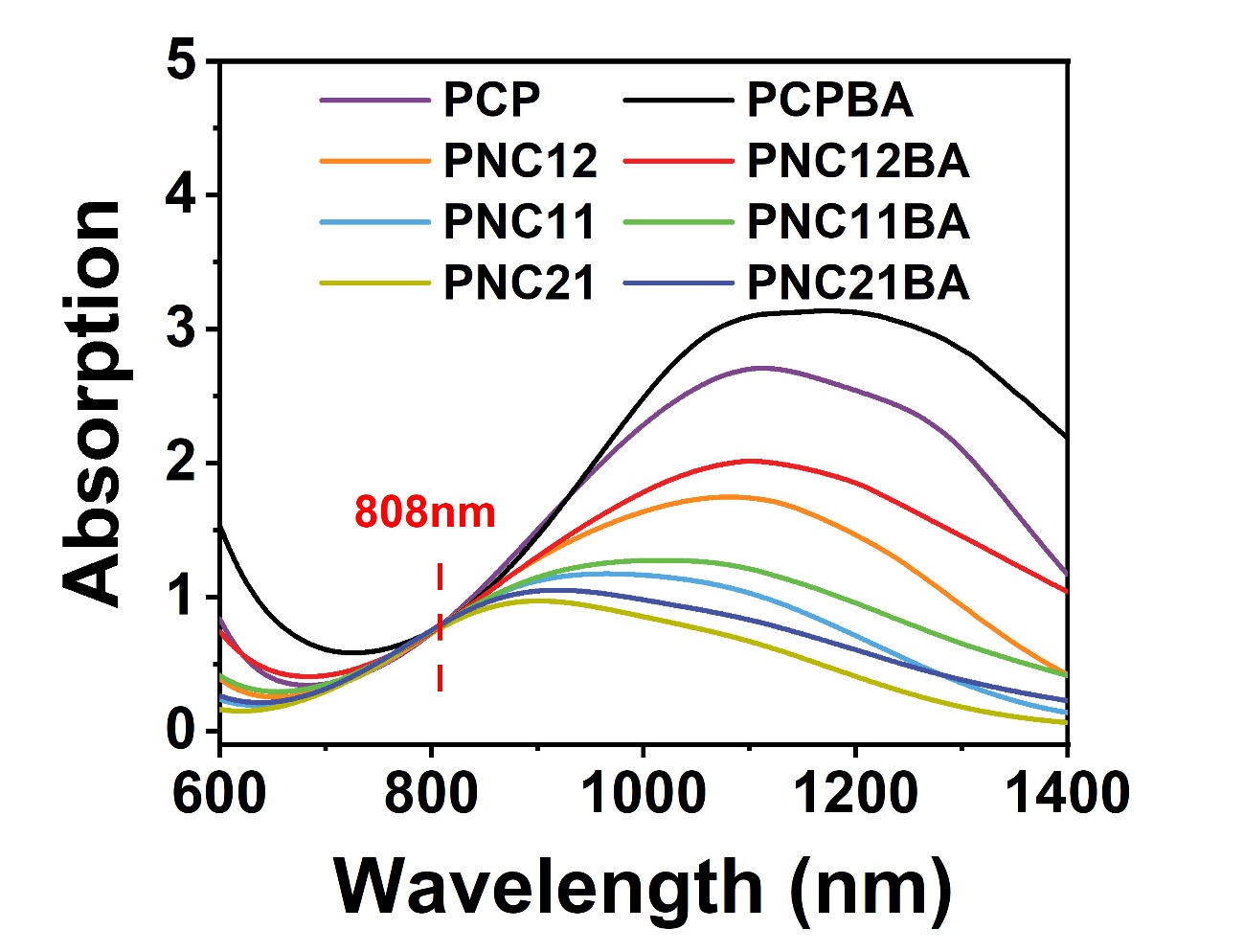


**Figure S18.** Absorption spectra of these eight polymers with the same absorbance at 808 nm.


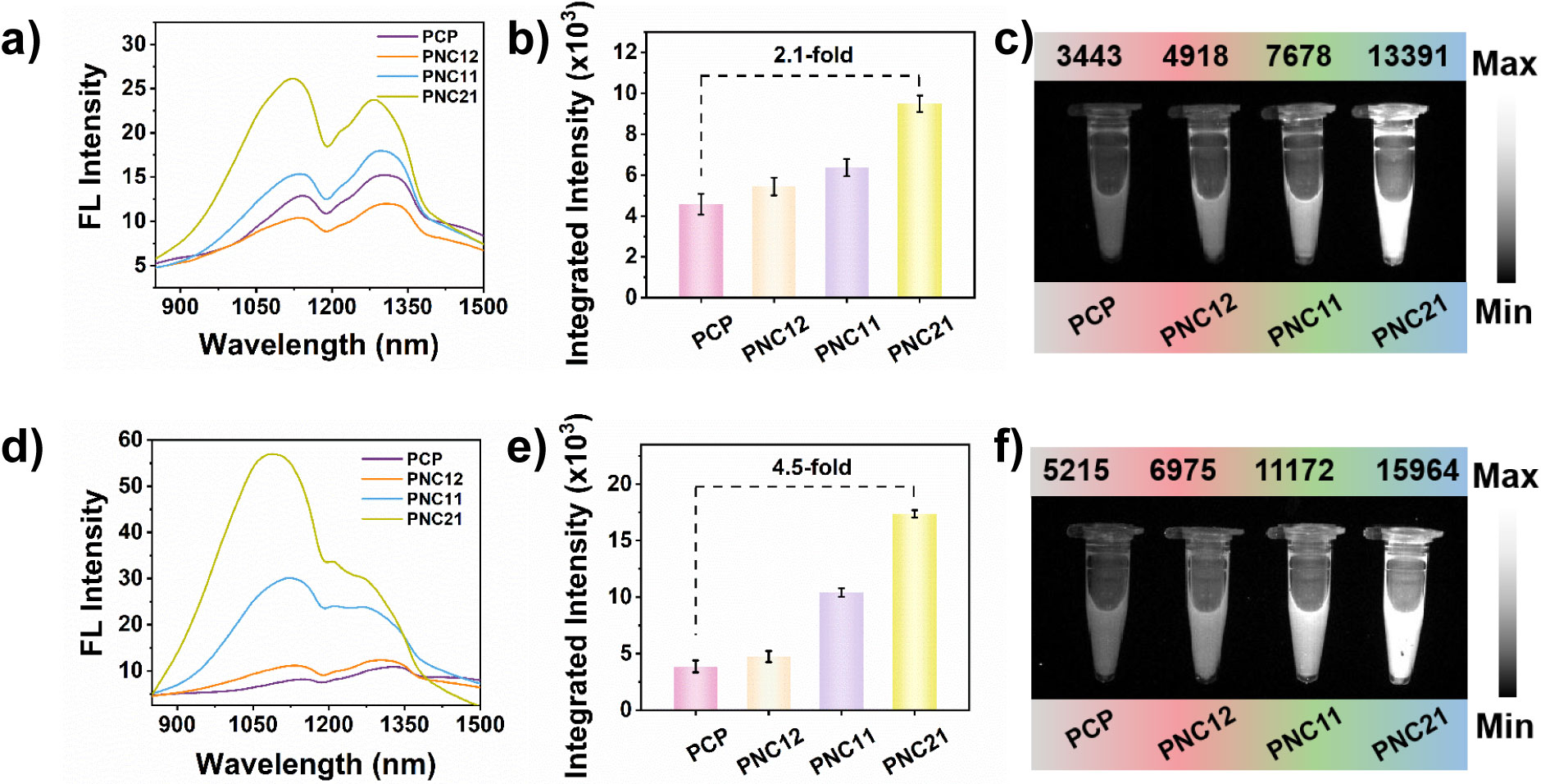


**Figure S19.** Characterization of PCP, PNC12, PNC11, and PNC21 in THF. (a) NIR-II fluorescence spectra, (b) Corresponding quantified integrated intensity (range, 900-1400 nm), and (c) NIR-II fluorescence images (808 nm laser excitation and 1064 nm long-pass filter) of four polymers with same concentration. (d) NIR-II fluorescence spectra, (e) Corresponding quantified integrated intensity (range, 900-1400 nm), and (f) NIR-II fluorescence images (808 nm laser excitation and 1064 nm long-pass filter) of four polymers with same absorbance at 808 nm. Error bars, mean ± SD.


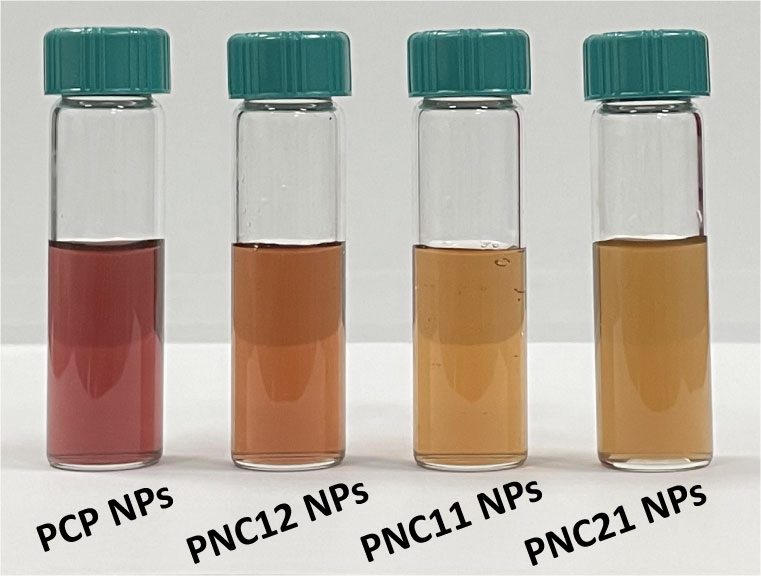


**Figure S20.** Photograph of water-soluble nanoparticles (0.1 mg mL^-1^).


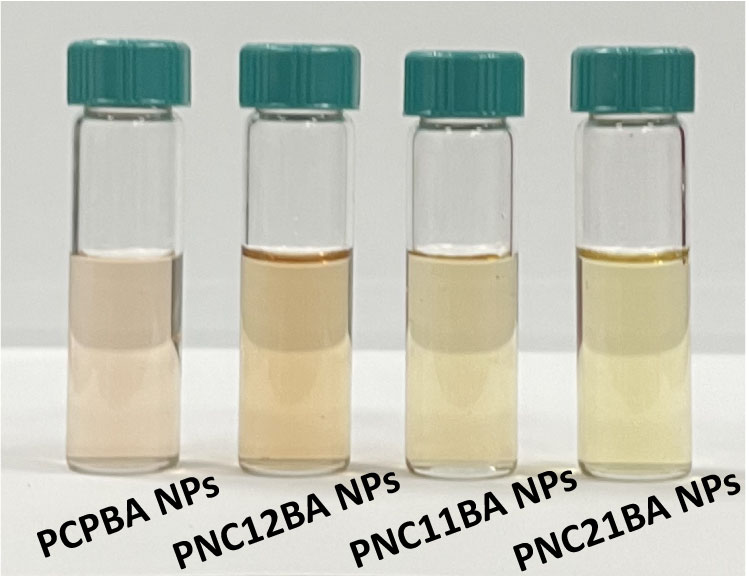


**Figure S21.** Photograph of water-soluble nanoparticles (0.1 mg mL^-1^).


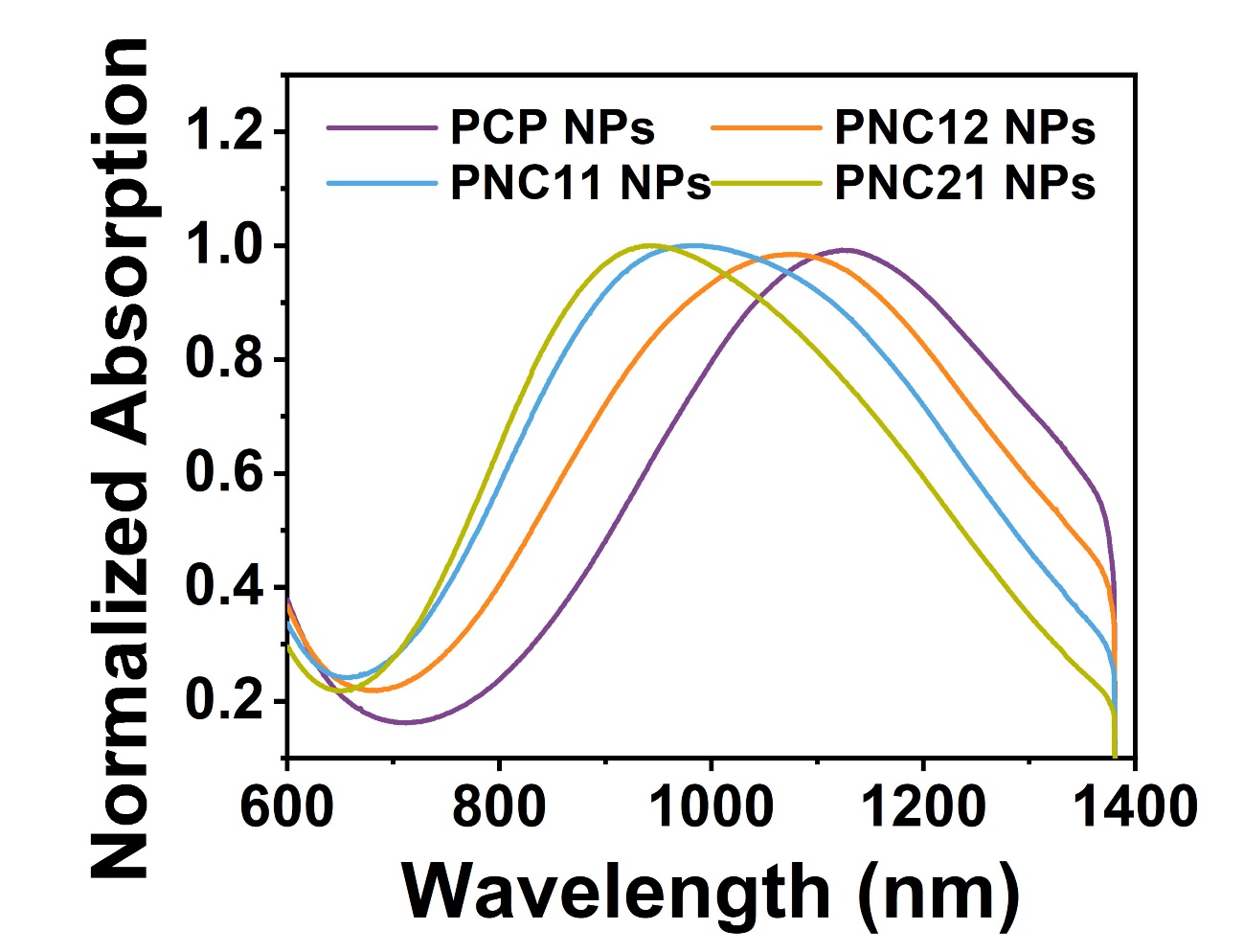


**Figure S22.** Normalized absorption spectra of PCP NPs, PNC12 NPs, PNC11 NPs, and PNC21 NPs in water.


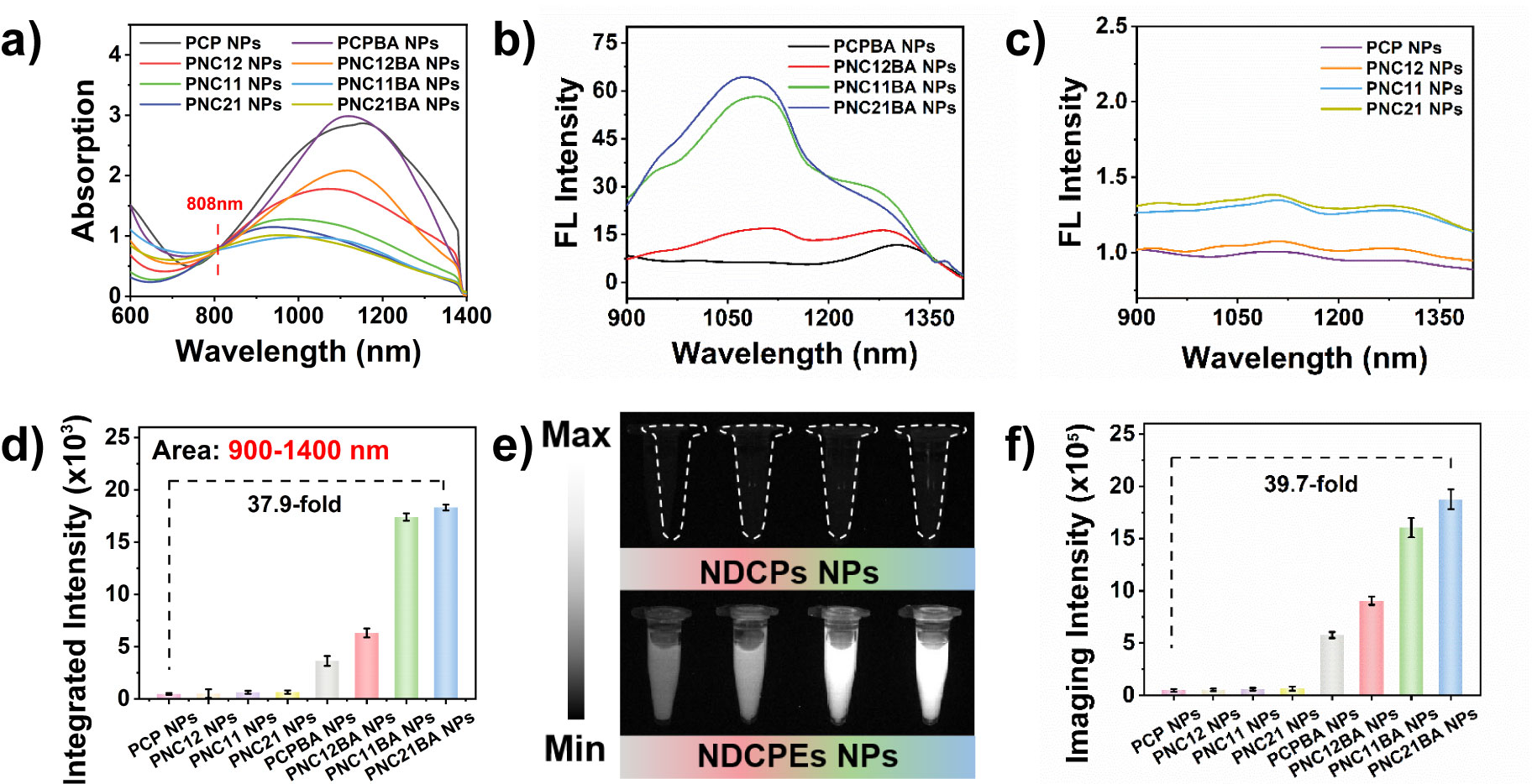


**Figure S23.** (a) Absorption spectra of NDCPEs NPs and NDCPs NPs with the same absorbance at 808 nm. NIR-II fluorescence spectra of (b) NDCPEs NPs and (c) NDCPs NPs with the same absorbance at 808 nm (under 808 nm light excitation). (d) Corresponding quantified fluorescence integrated intensity (range, 900-1400 nm) of (b) and (c). (e) NIR-II fluorescence images (808 nm laser excitation and 1064 nm long-pass filter) and (f) corresponding imaging intensities of NDCPs NPs (up, left to right: PCP NPs, PNC12 NPs, PNC11 NPs, and PNC21 NPs) and NDCPEs NPs (down, left to right: PCPBA NPs, PNC12BA NPs, PNC11BA NPs, and PNC21BA NPs) with the same absorbance at 808 nm. Error bars, mean ± SD.


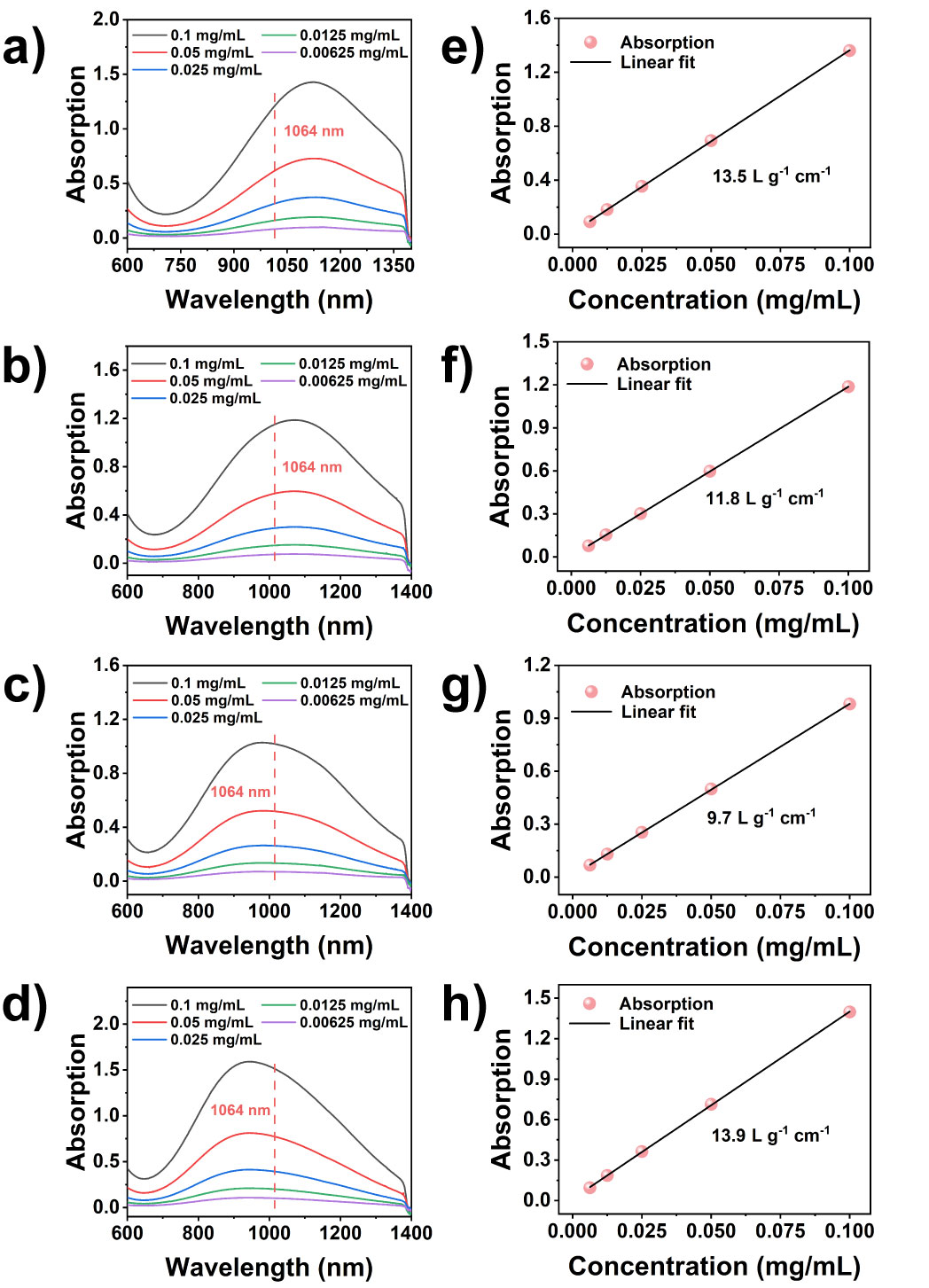


**Figure S24.** UV-vis-NIR absorption spectra of (a) PCP NPs, (b) PNC12 NPs, (c) PNC11 NPs and (d) PNC21 NPs in water at different concentrations, and the extinction coefficients (e) PCP NPs, (f) PNC12 NPs, (g) PNC11 NPs and (h) PNC21 NPs at corresponding peaks of 1064 nm, respectively.


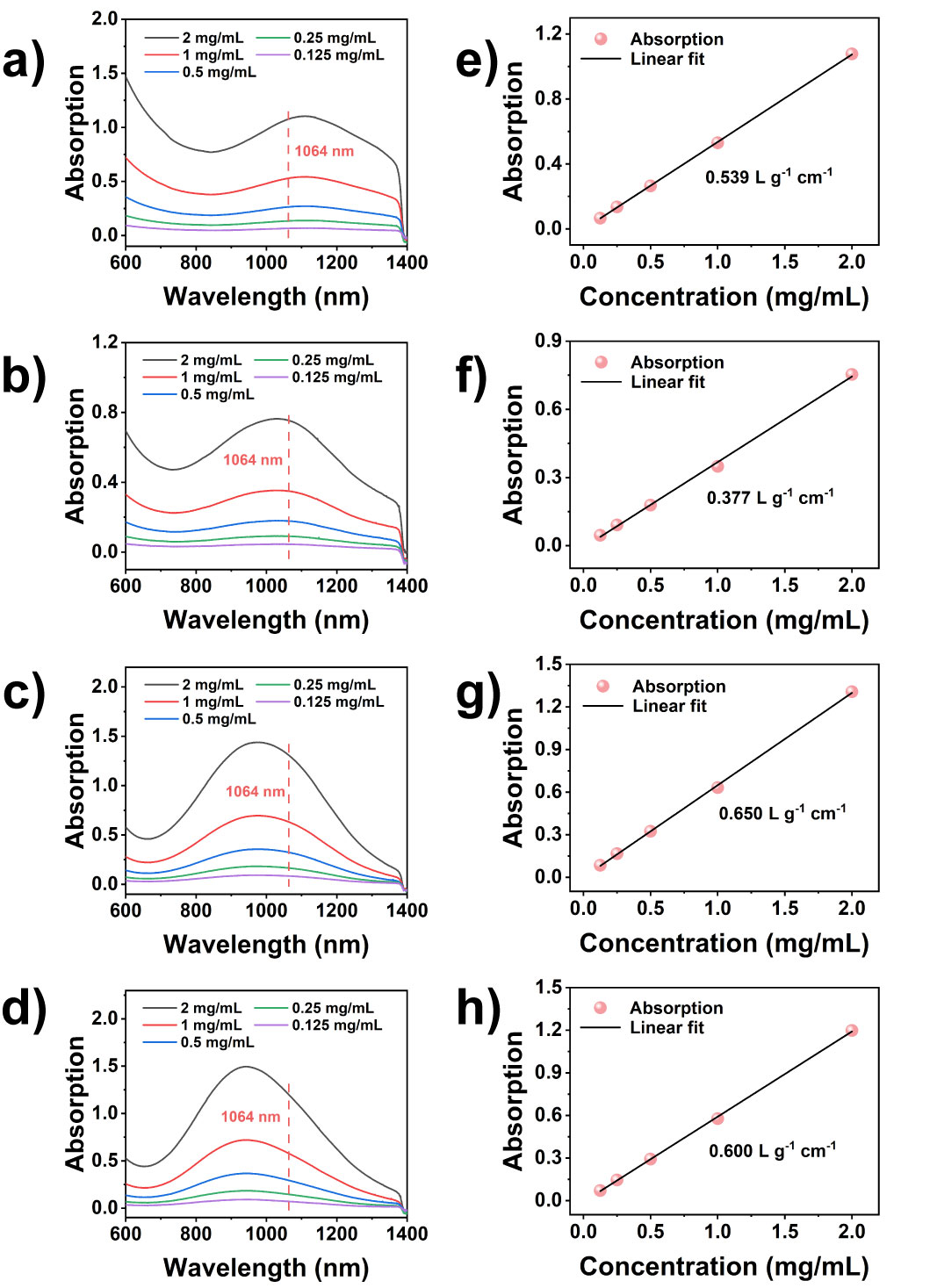


**Figure S25.** UV-vis-NIR absorption spectra of (a) PCPBA NPs, (b) PNC12BA NPs, (c) PNC11BA NPs and (d) PNC21BA NPs in water at different concentrations, and the extinction coefficients (e) PCPBA NPs, (f) PNC12BA NPs, (g) PNC11BA NPs and (h) PNC21BA NPs at corresponding peaks of 1064 nm, respectively.


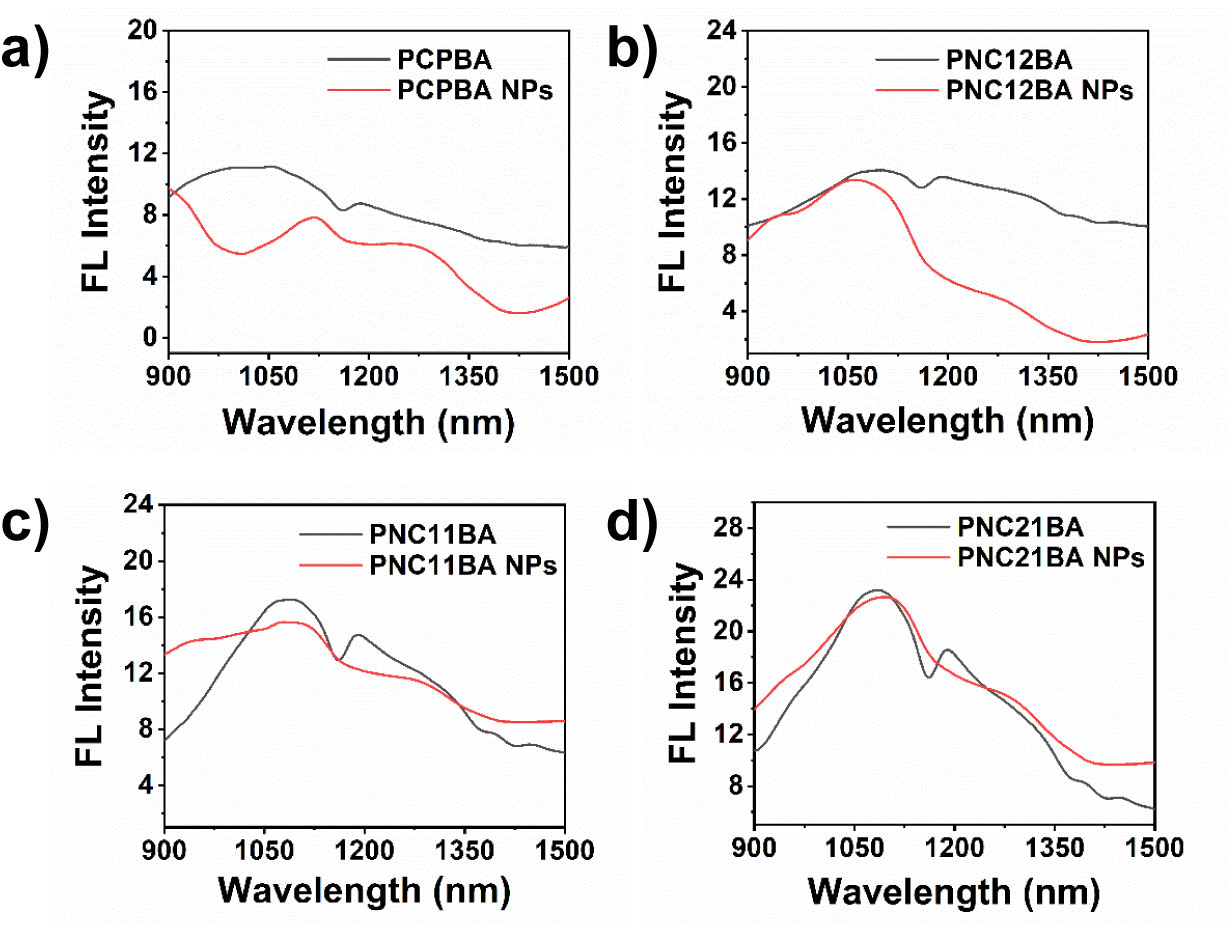


**Figure S26.** NIR-II fluorescence intensity performance of PCPBA NPs, PNC12BA NPs, PNC11BA NPs, and PNC21BA NPs in aqueous solution with PCPBA, PNC12BA, PNC11BA, and PNC21BA in DCM solution.


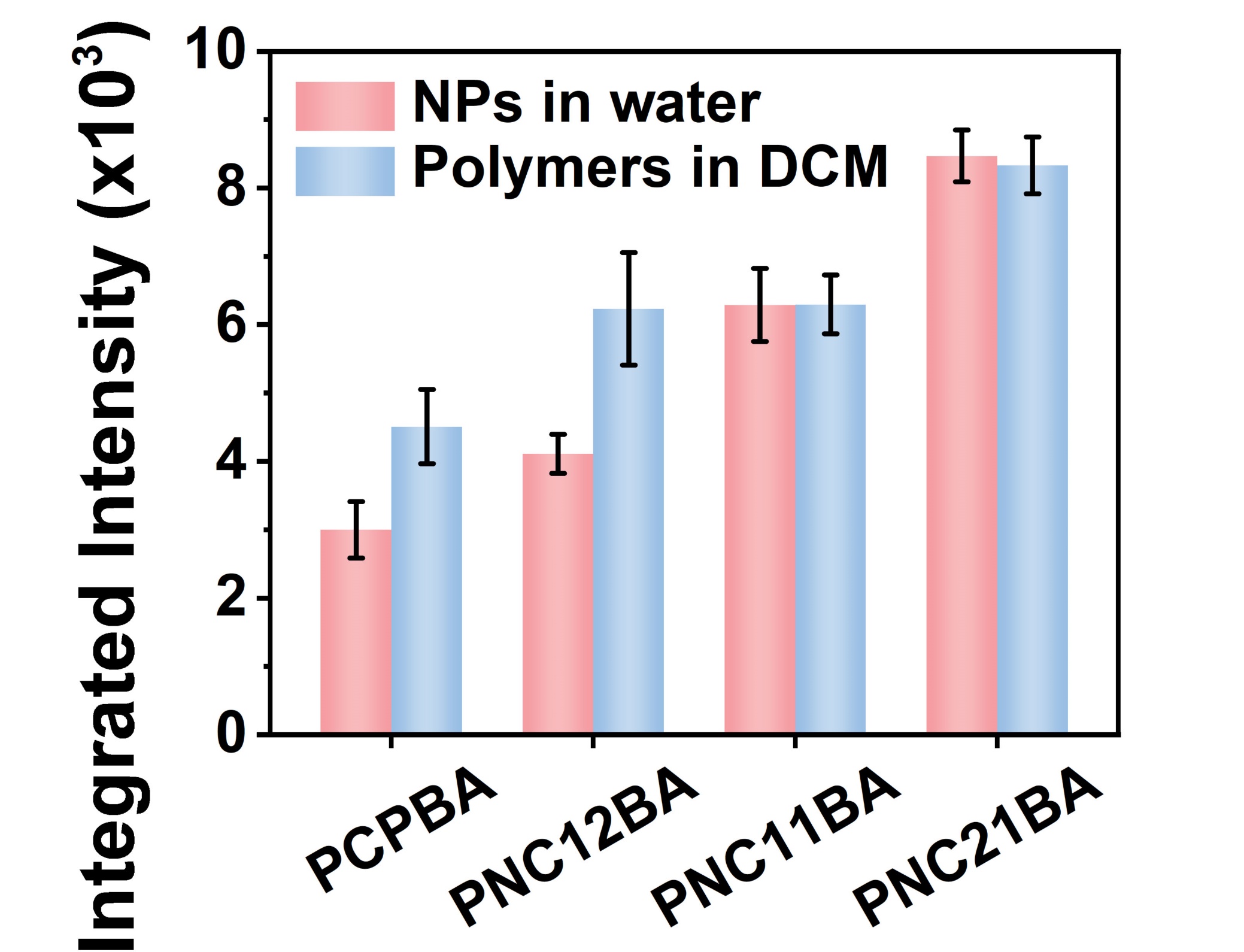


**Figure S27.** The NIR-II fluorescence integrated intensity (range, 900-1400 nm) of NDCPEs in DCM solution and in aqueous solution at same concentration. Error bars, mean ± SD.


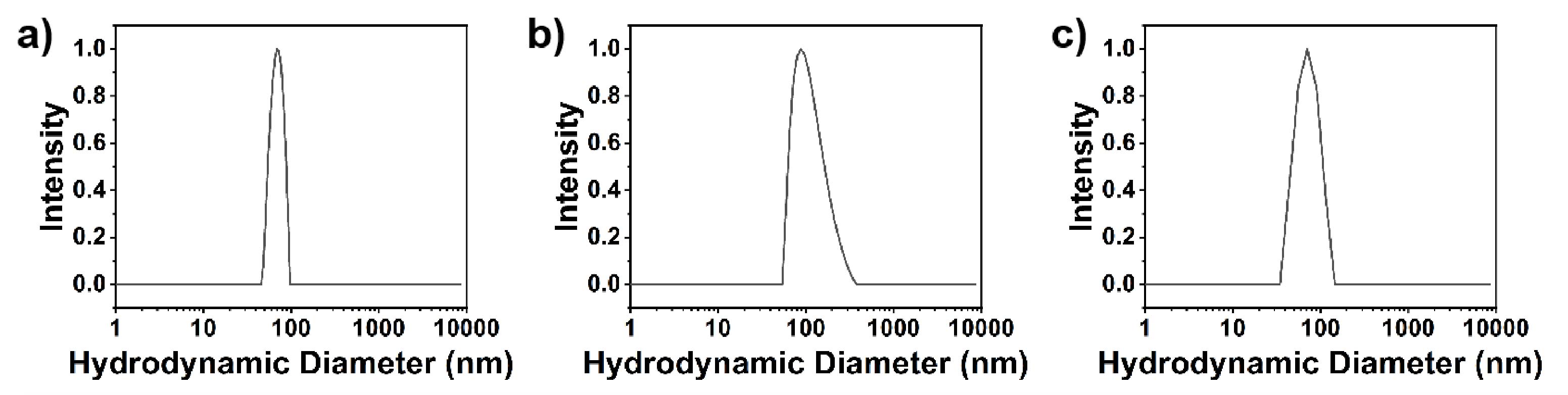


**Figure S28.** *D*_h_ of (a) PBT, (b) PBT/NO and (c) PBT/Pt measured by DLS.


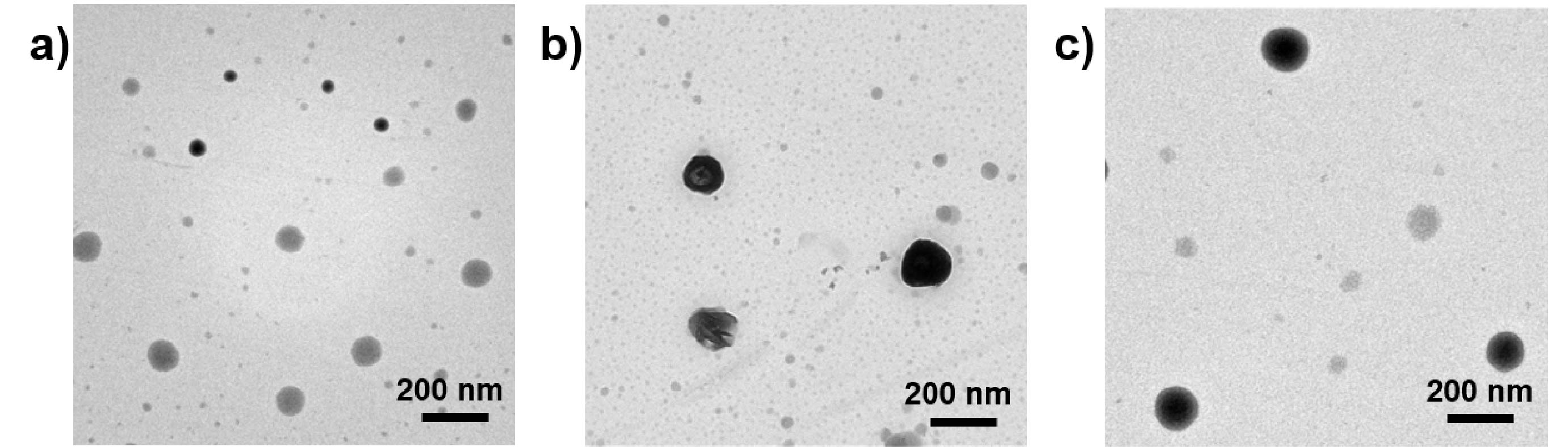


**Figure S29.**  TEM images of (a) PBT, (b) PBT/NO and (c) PBT/Pt. (Scale bar: 200 nm).


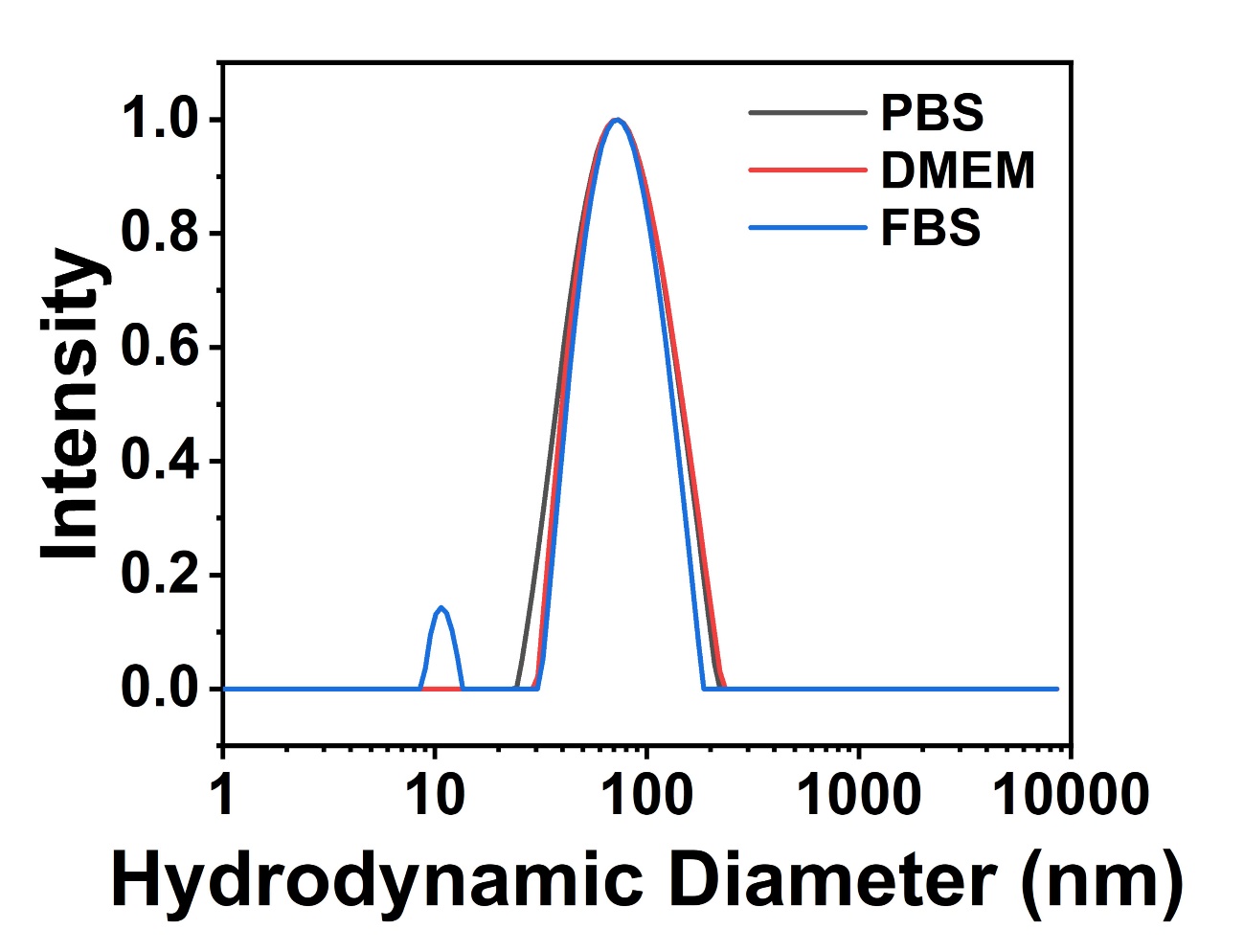


**Figure S30.** Average hydrodynamic diameter (*D*_h_) of PBT/NO/Pt was measured in different solutions (DMEM, FBS, and PBS) using DLS.


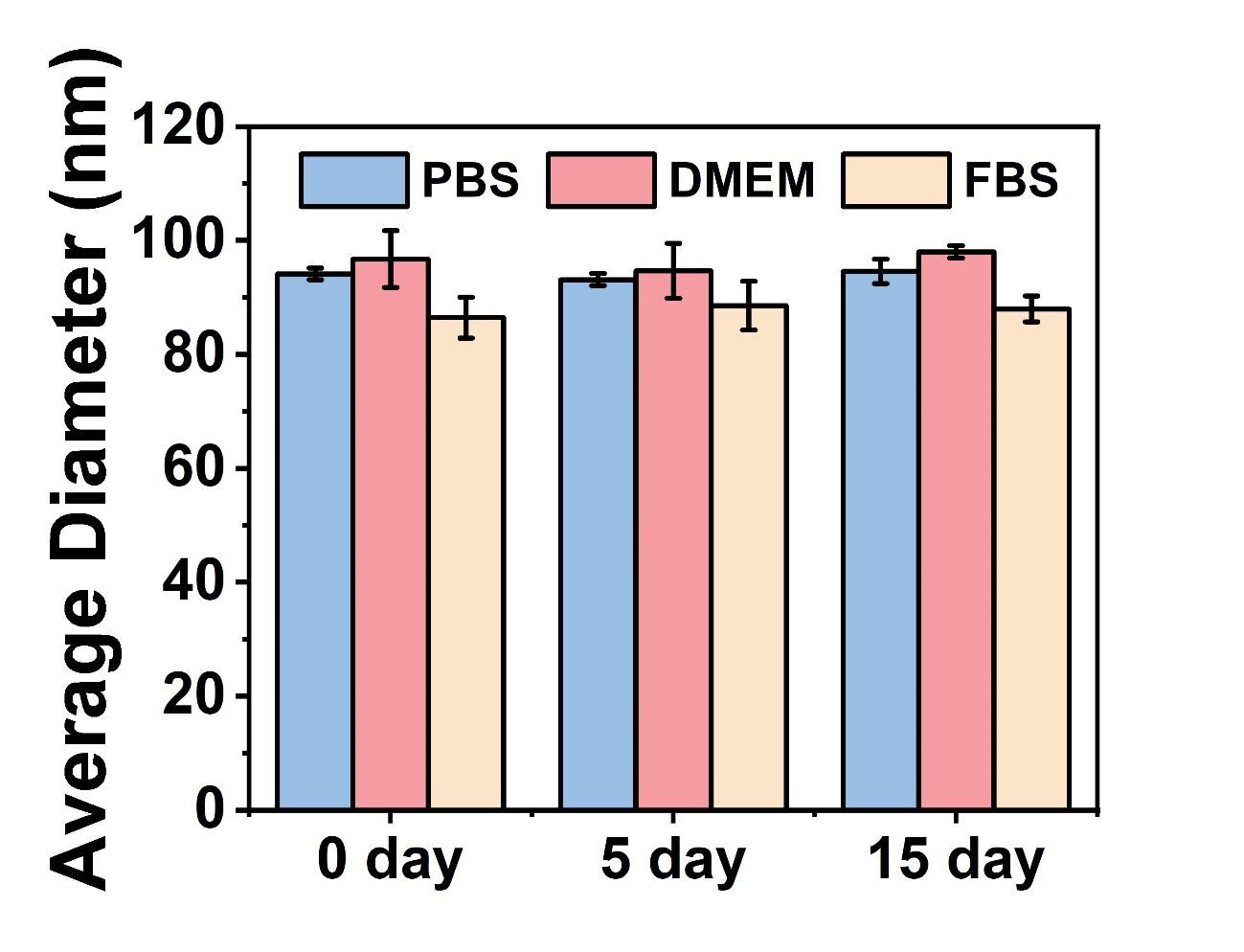


**Figure S31.** Average hydrodynamic diameter (*D*_h_) of PBT/NO/Pt in PBS, DMEM, or FBS for different time periods. Error bars, mean ± SD (n = 3).


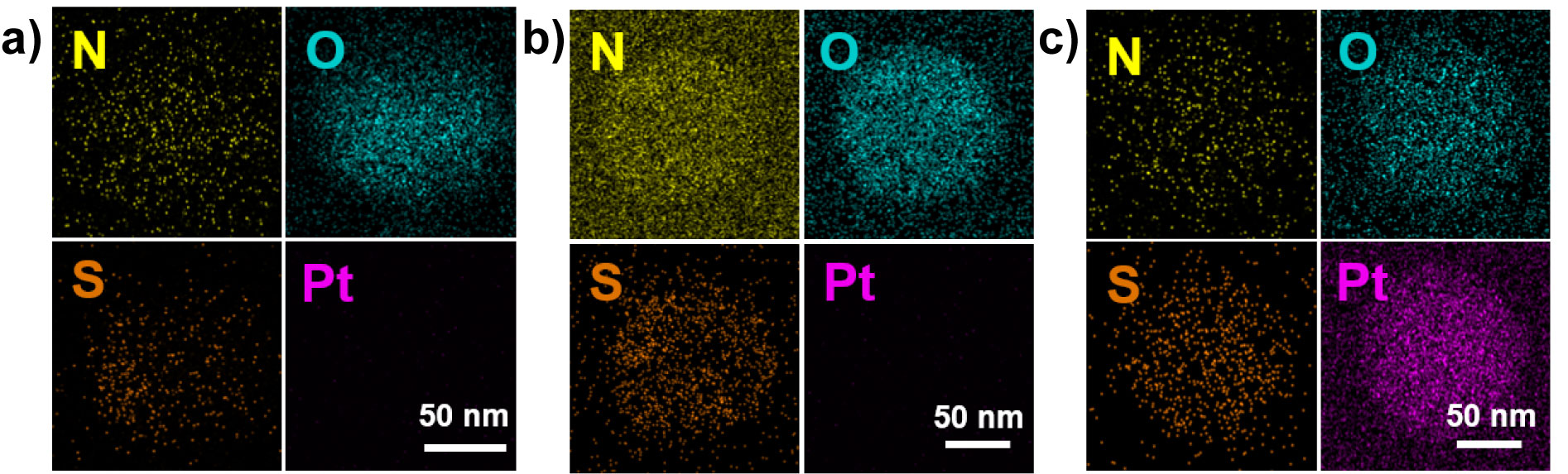


**Figure S32.** TEM-EDS element mapping of (a) PBT, (b) PBT/NO and (c) PBT/Pt. (Scale bar: 50 nm).


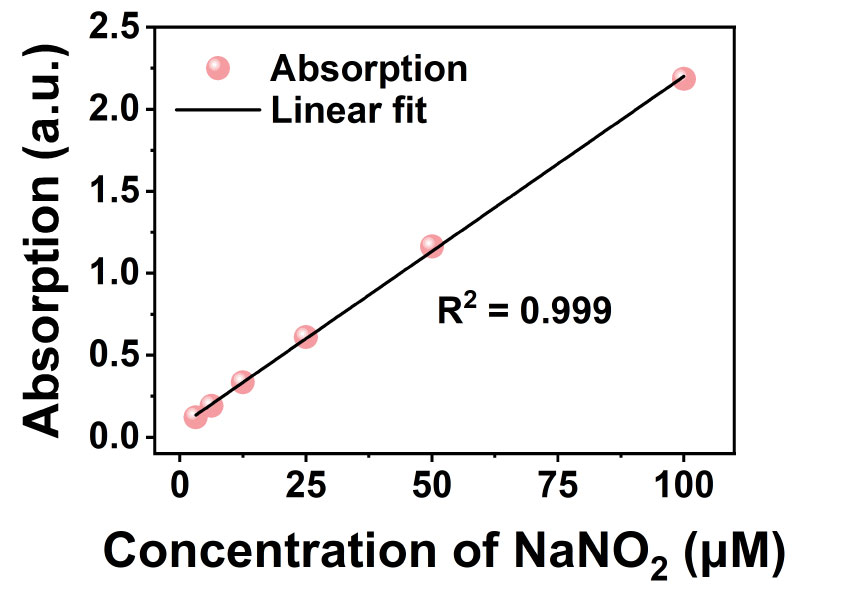


**Figure S33.** The standard curves obtained with different concentration by microplate reader.


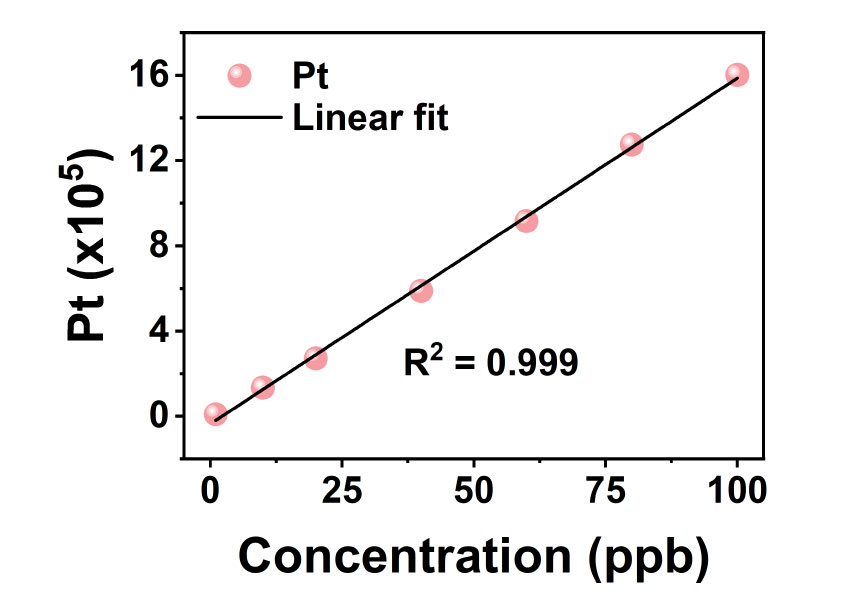


**Figure S34.** The standard curves are obtained with different concentration by inductively coupled plasma mass spectrometer (ICP-MS).


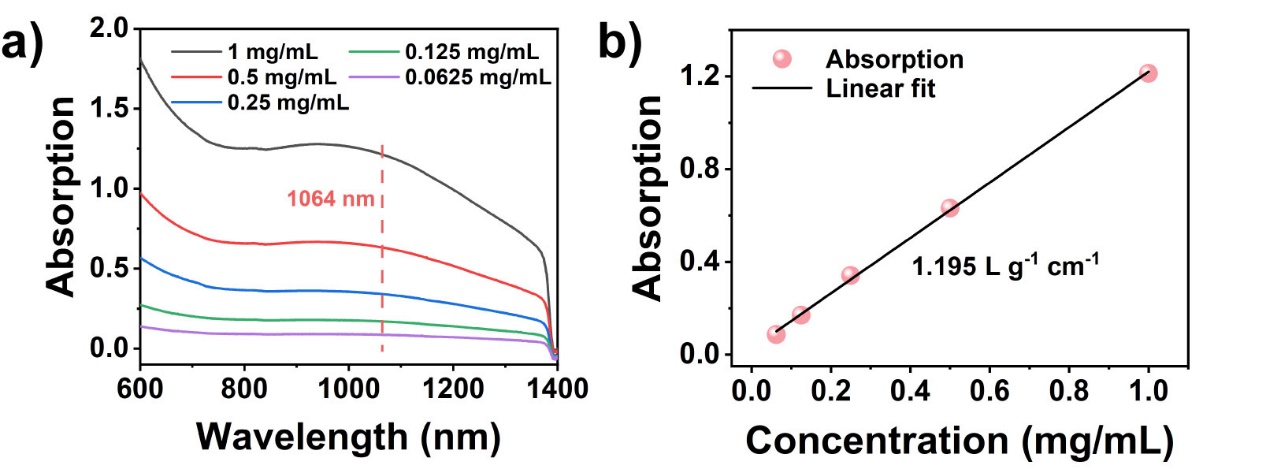


**Figure S35.** (a) UV-vis-NIR absorption spectra of PBT/NO/Pt at different concentrations, (b) and the extinction coefficients at the corresponding peaks of 1064 nm.


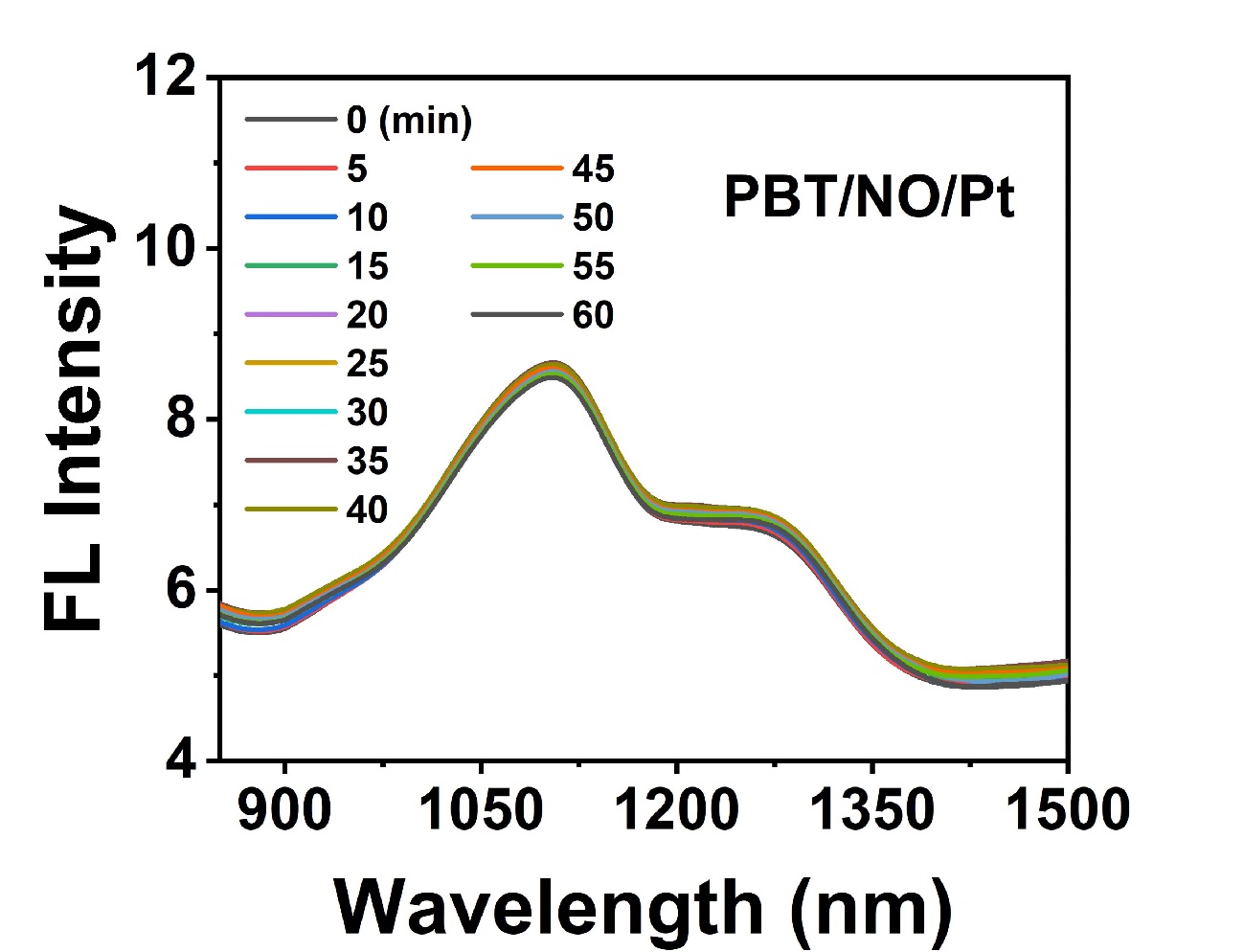


**Figure S36.** Fluorescence emission spectra recorded for PBT/NO/Pt after exposed to continuous illumination at 808 nm (0.5 W cm^−2^) for 60 min.


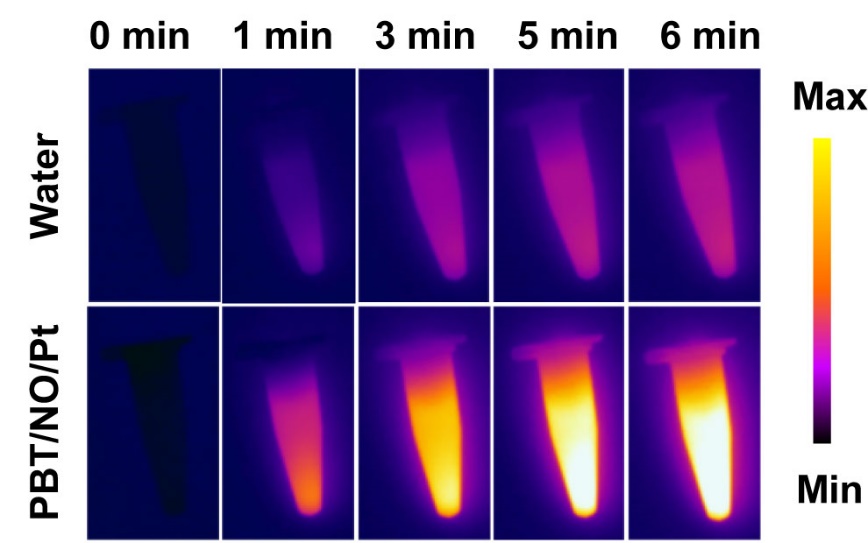


**Figure S37.** Photothermal experiment real-time imaging of water and PBT/NO/Pt.


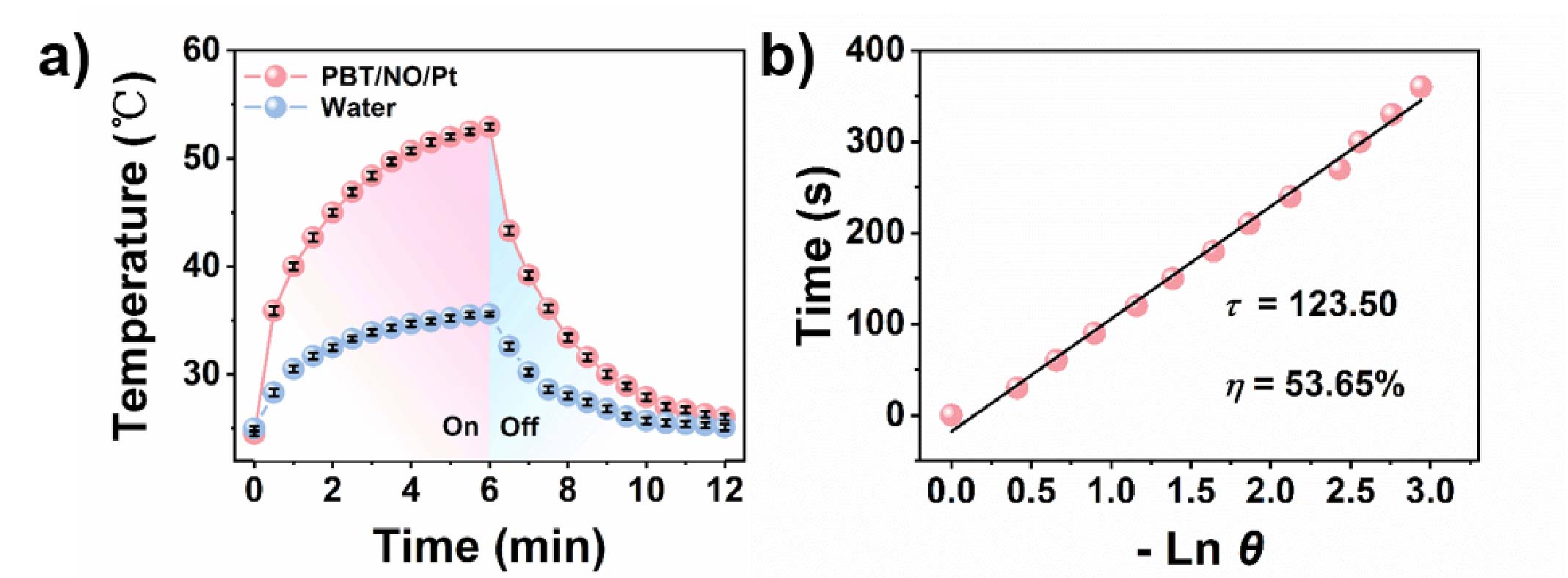


**Figure S38.** (a) Photothermal heating curves of water and PBT/NO/Pt (0.1 mg mL^−1^) under 1064 nm (1.0 W cm^−2^) laser irradiation, respectively. Error bars, mean ± SD (n = 3). (b) Cooling time versus negative natural logarithm of the driving force temperature.


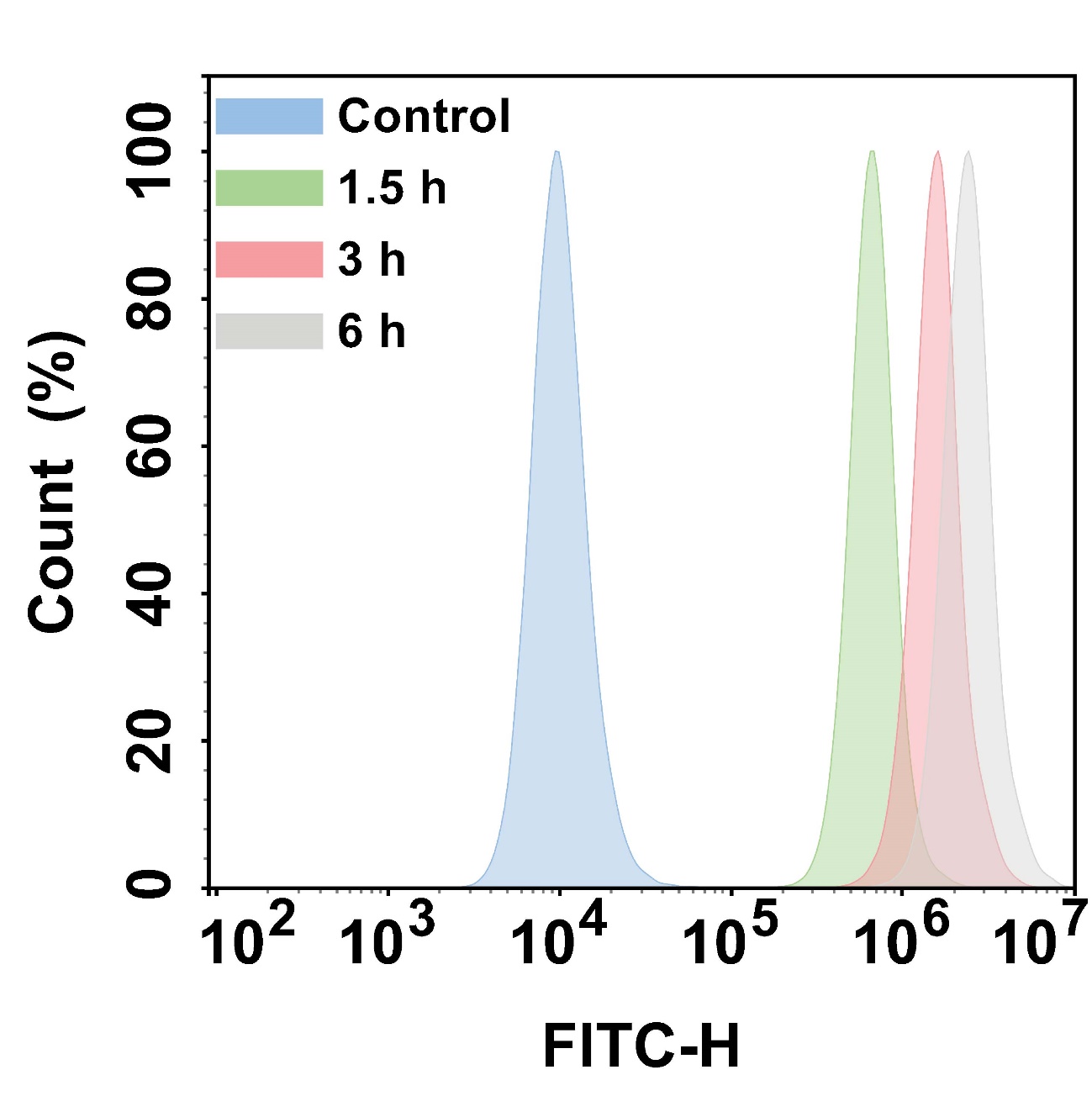


**Figure S39.** Cellular uptake of the PBT/NO/Pt after various incubation times is determined by flow cytometry.


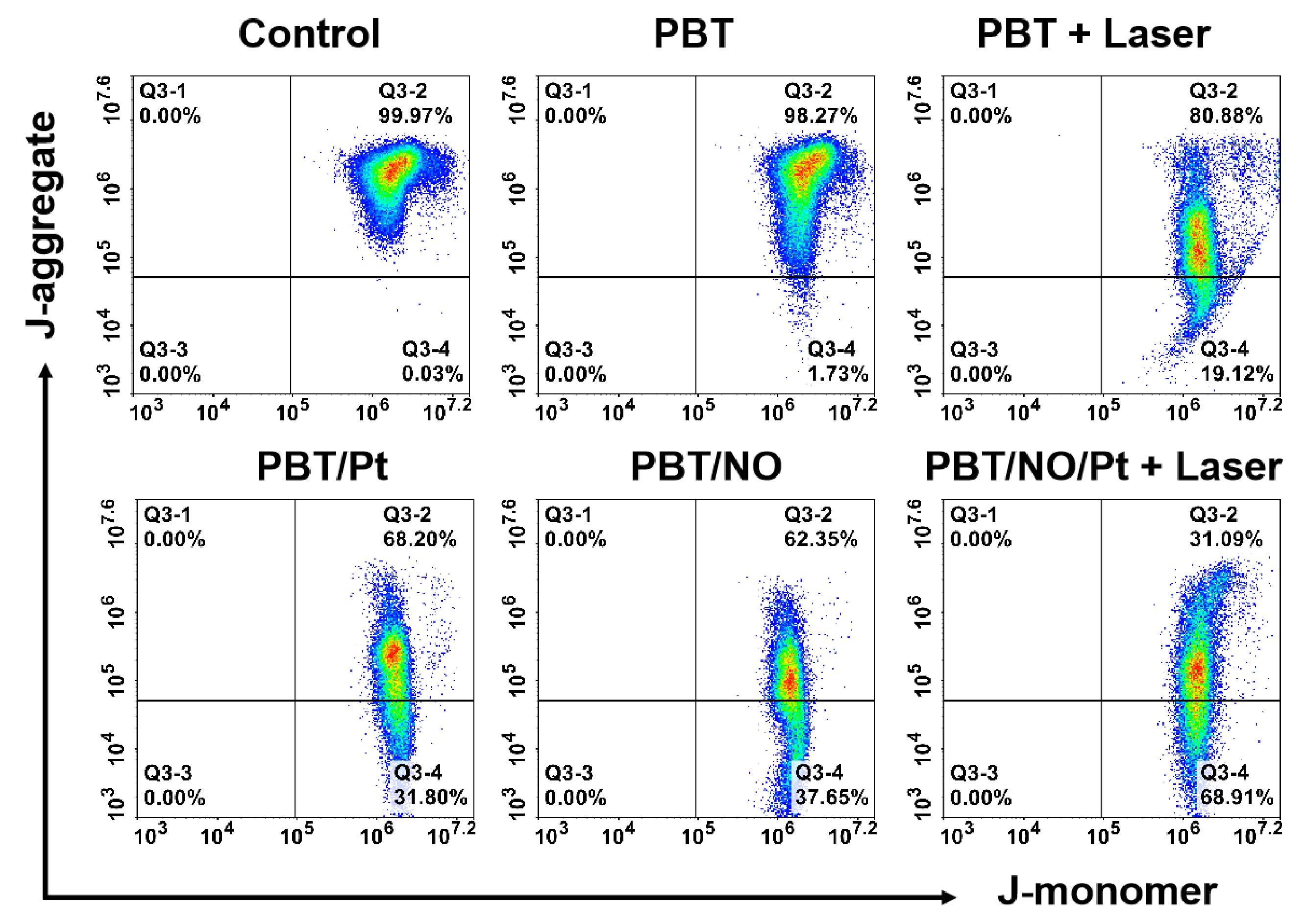


**Figure S40.** JC-1 stained of SKOV3/DDP cells after different treatments determined by flow cytometry.


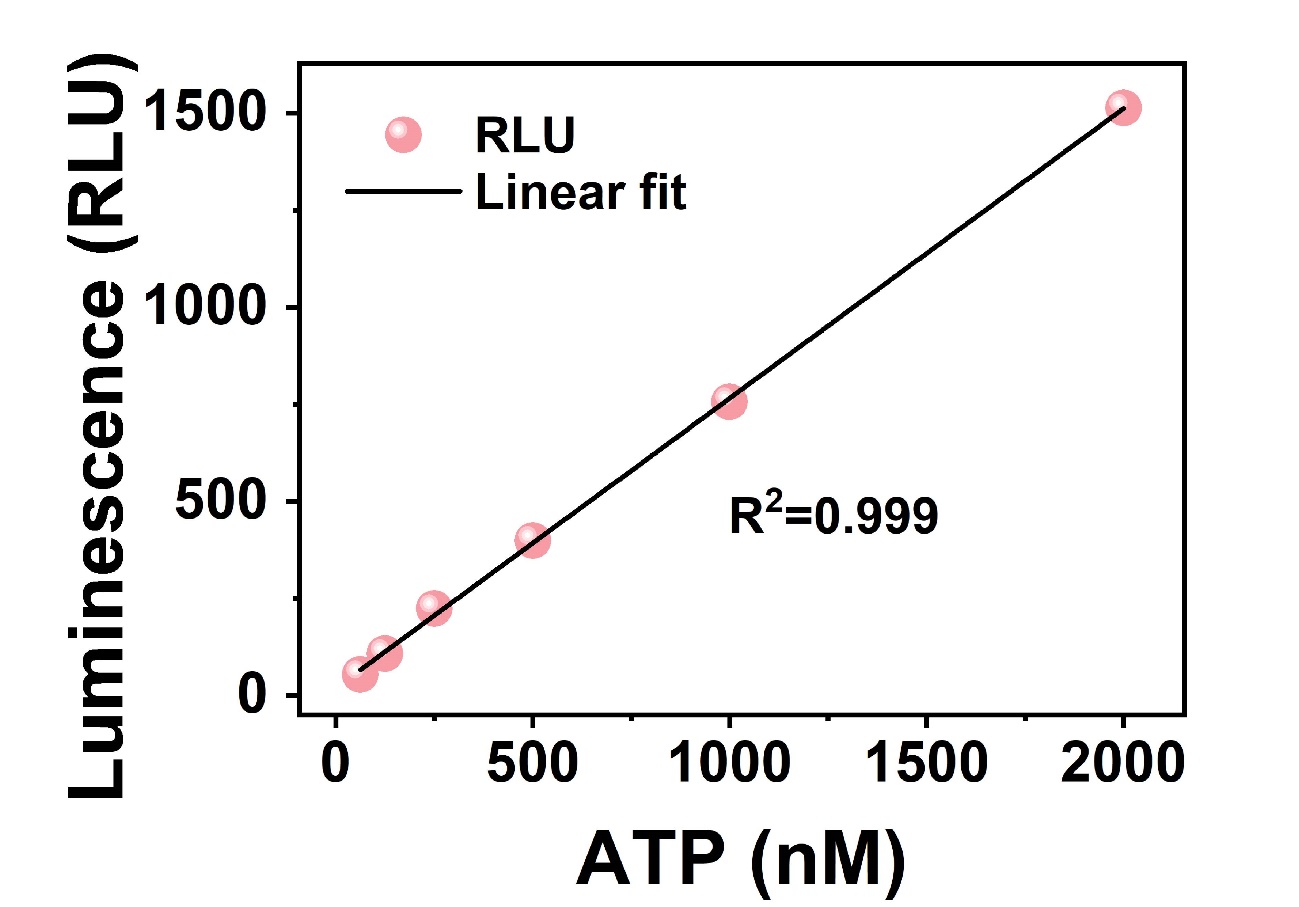


**Figure S41.** The standard curves of ATP obtained with different concentration by microplate reader.


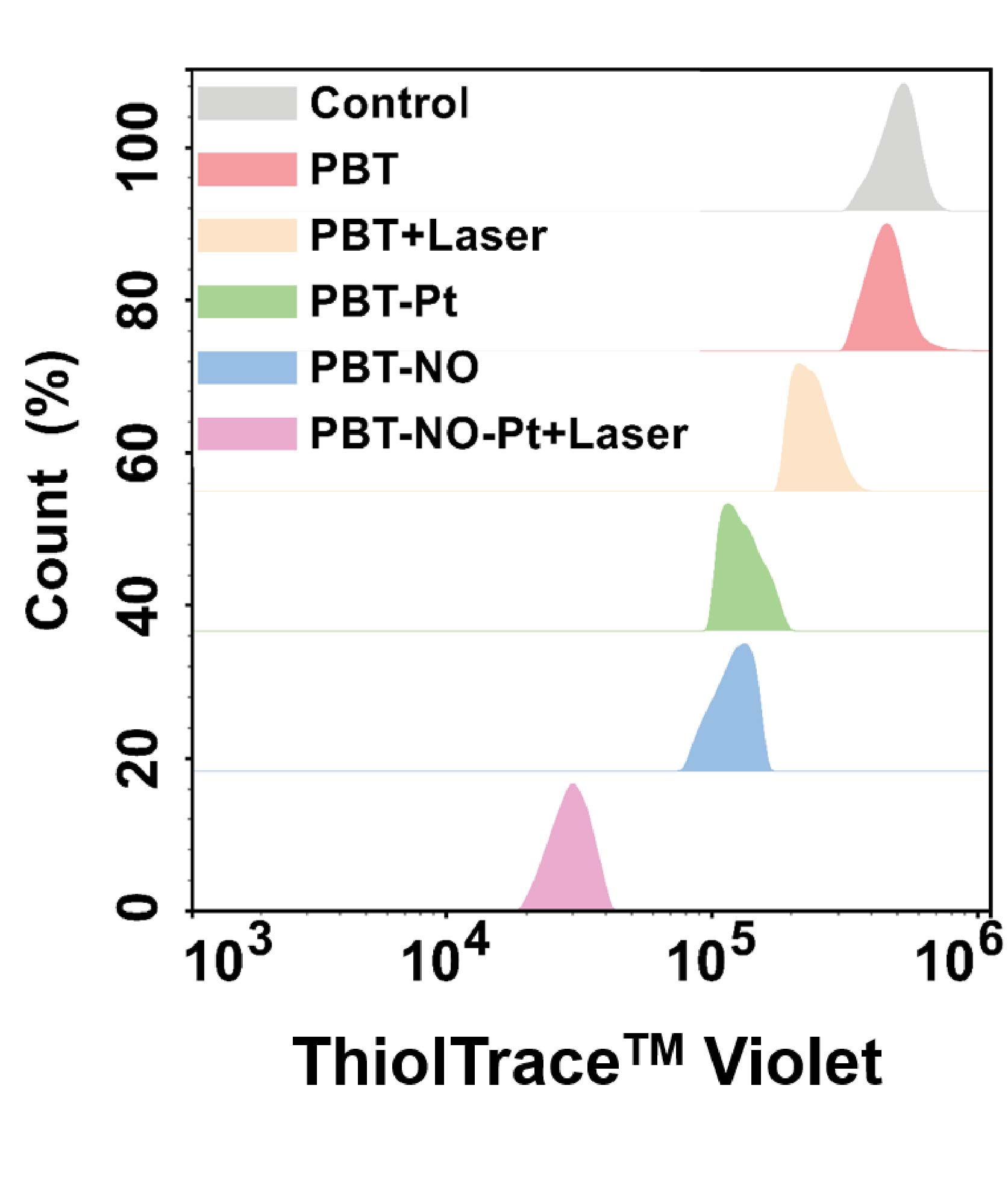


**Figure S42.** ThioTracker Violet stained of SKOV3/DDP cells after different treatments determined by flow cytometry.

**
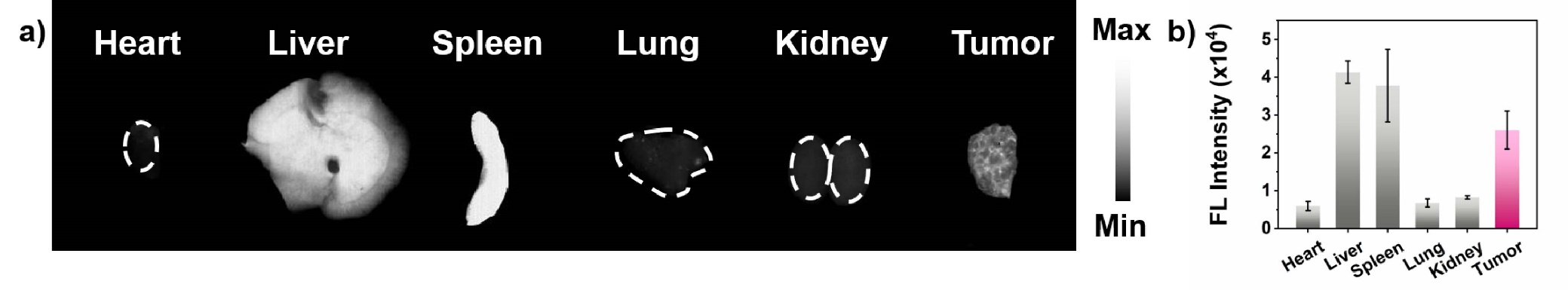
**

**Figure S43.** a) *Ex vivo* NIR-II fluorescence images of the tumor and main organs of PBT/NO/Pt after 24 h intravenous administration. b) Corresponding signal quantification. Error bars, mean ± SD (n = 3).


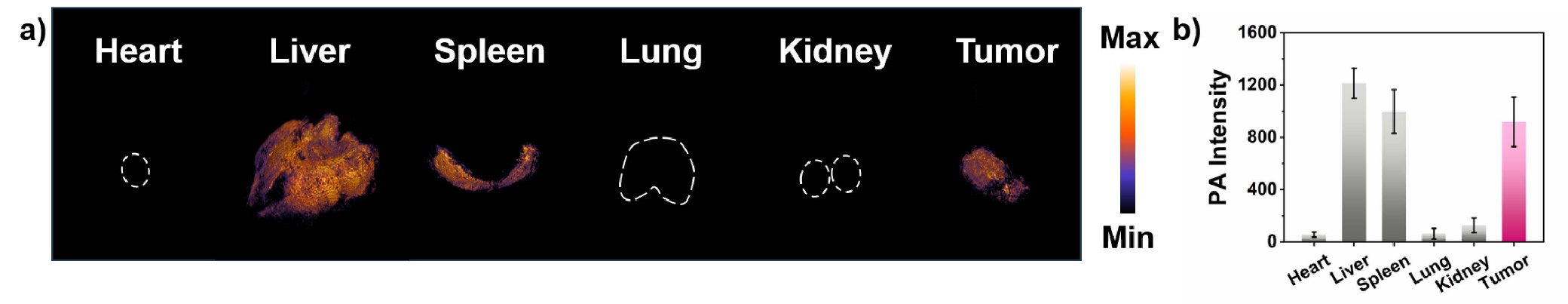


**Figure S44.** a) *Ex vivo* NIR-II PA images of the tumor and main organs of PBT/NO/Pt after 24 h intravenous administration. b) Corresponding signal quantification. Error bars, mean ± SD (n = 3).


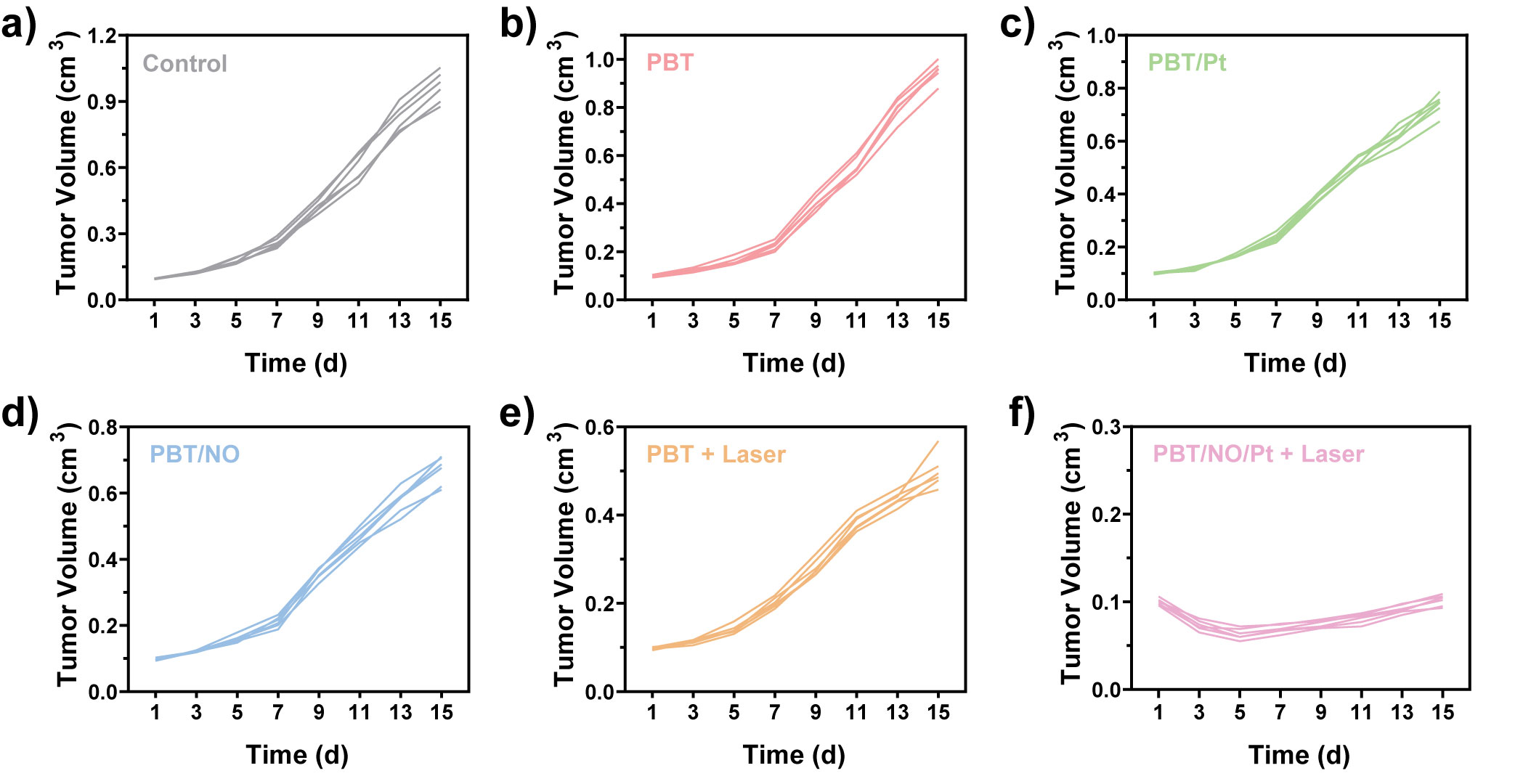


**Figure S45**. Tumor volume change of SKOV3 tumor-bearing mice for each individual animal after treated with a) PBS, b) PBT, c) PBT/Pt, d) PBT/NO, e) PBT + 1064 nm Laser and f) PBT/NO/Pt + 1064 nm Laser (1.0 W cm^-2^).


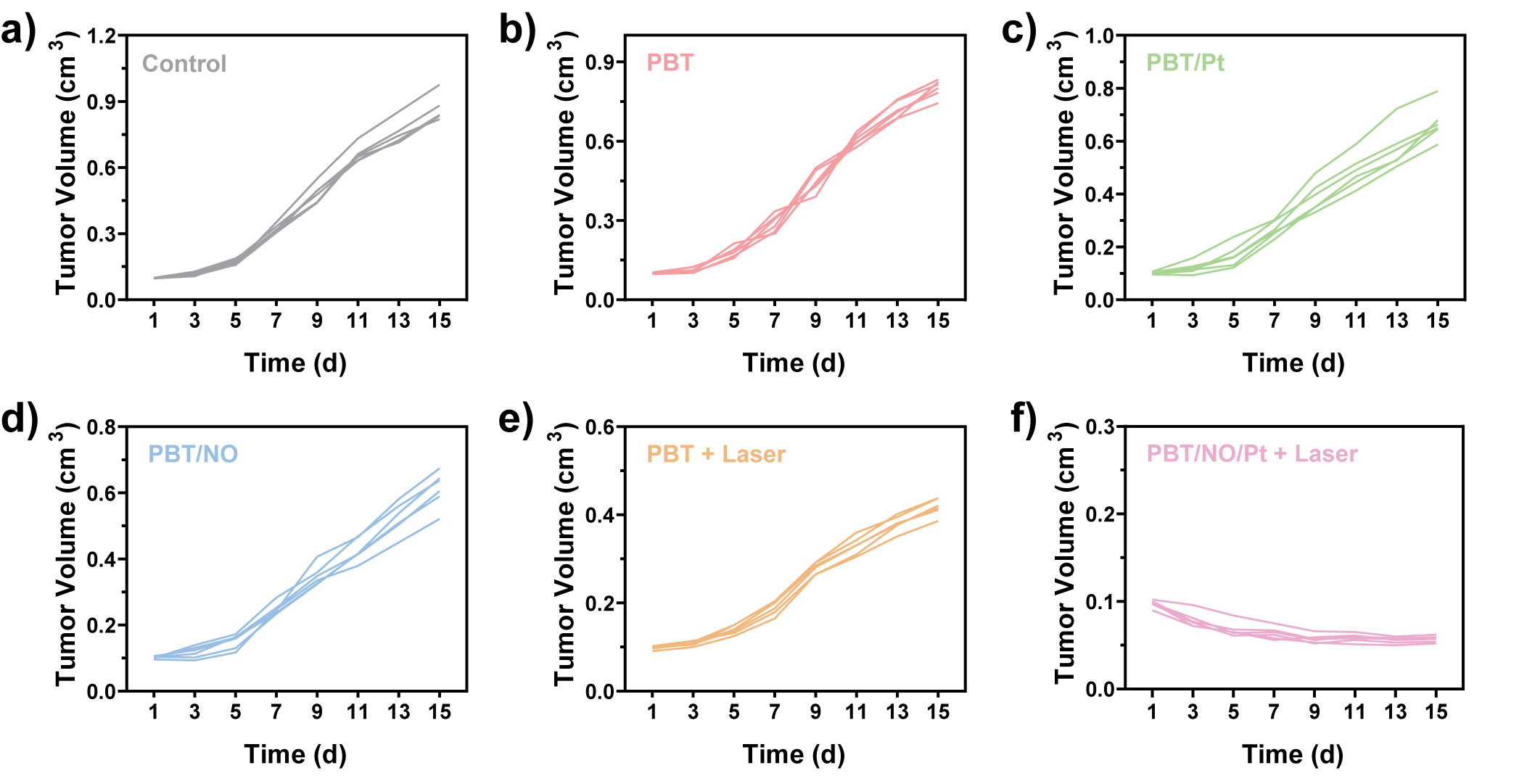


**Figure S46**. Tumor volume change of SKOV3/DDP tumor-bearing mice for each individual animal after treated with a) PBS, b) PBT, c) PBT/Pt, d) PBT/NO, e) PBT + 1064 nm Laser and f) PBT/NO/Pt + 1064 nm Laser (1.0 W cm^-2^).


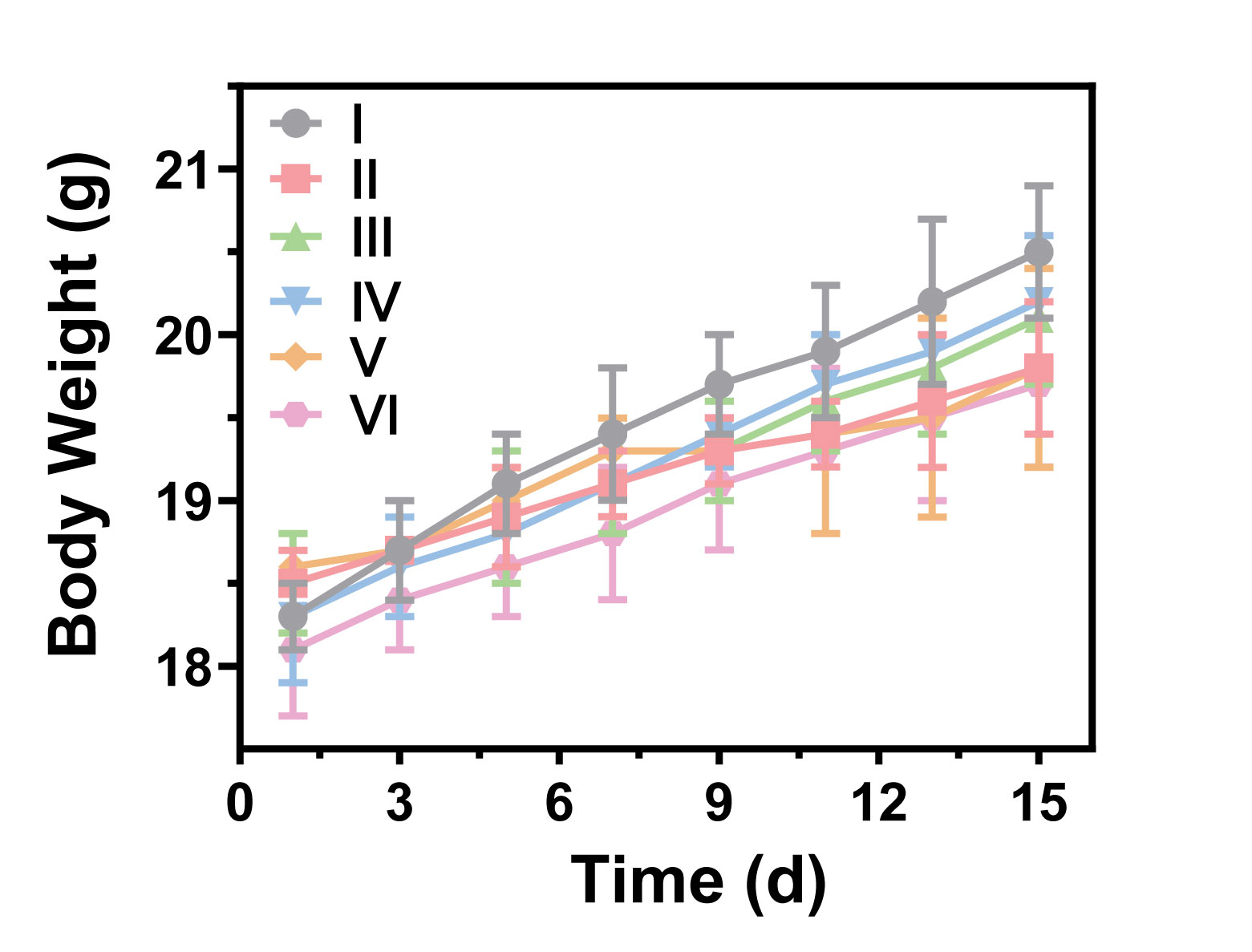


**Figure S47.** Body weight of mice with SKOV3 tumor under different treatment (I: PBS, II: PBT, III: PBT/Pt, IV: PBT/NO, Ⅴ: PBT + 1064 nm Laser, Ⅵ: PBT/NO/Pt + 1064 nm Laser, 1.0 W cm^-2^). Error bars, mean ± SD (n = 6).


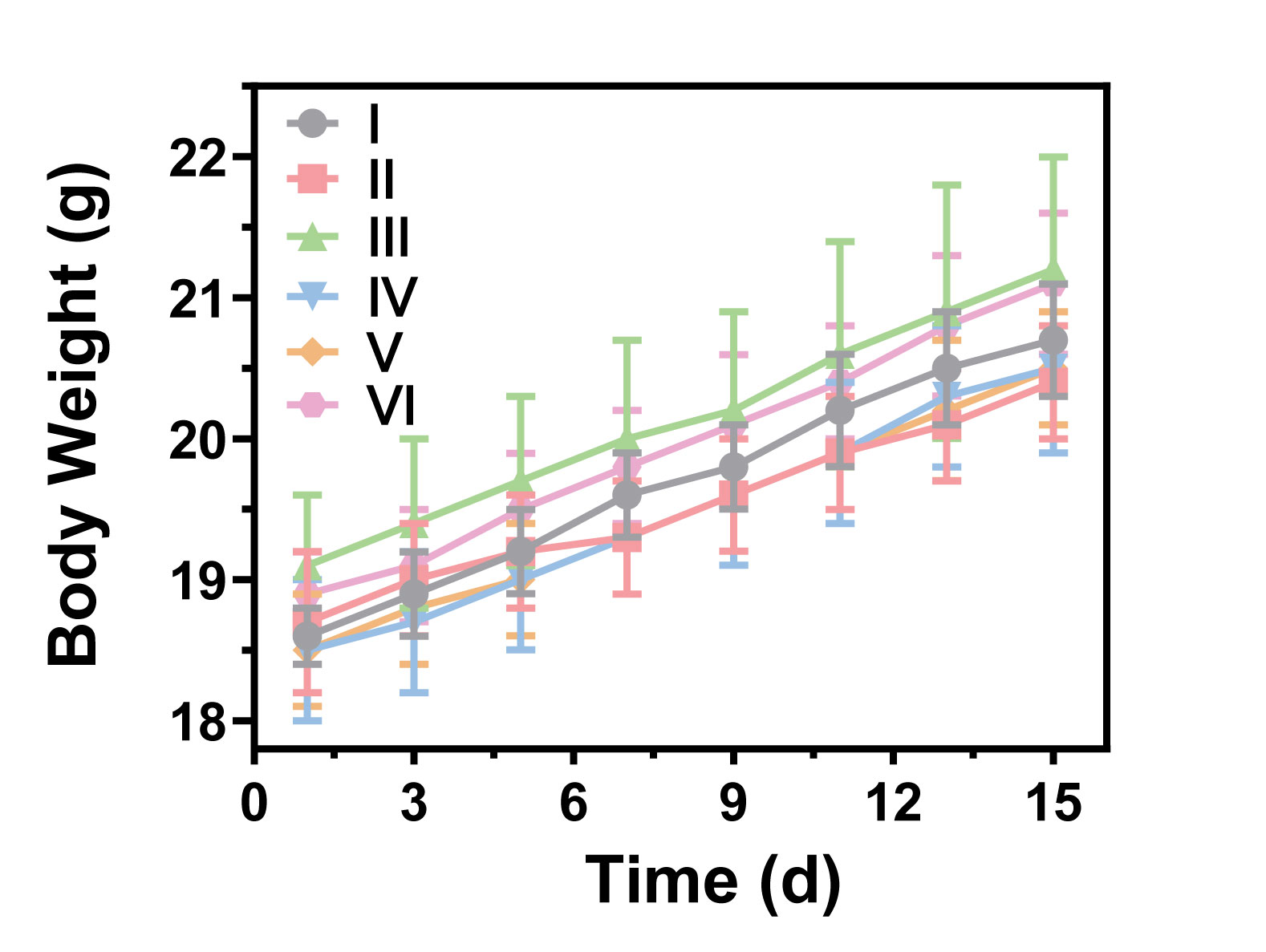


**Figure S48.** Body weight of mice with SKOV3/DDP tumor under different treatment (I: PBS, II: PBT, III: PBT/Pt, IV: PBT/NO, Ⅴ: PBT + 1064 nm Laser, Ⅵ: PBT/NO/Pt + 1064 nm Laser, 1.0 W cm^-2^). Error bars, mean ± SD (n = 6).


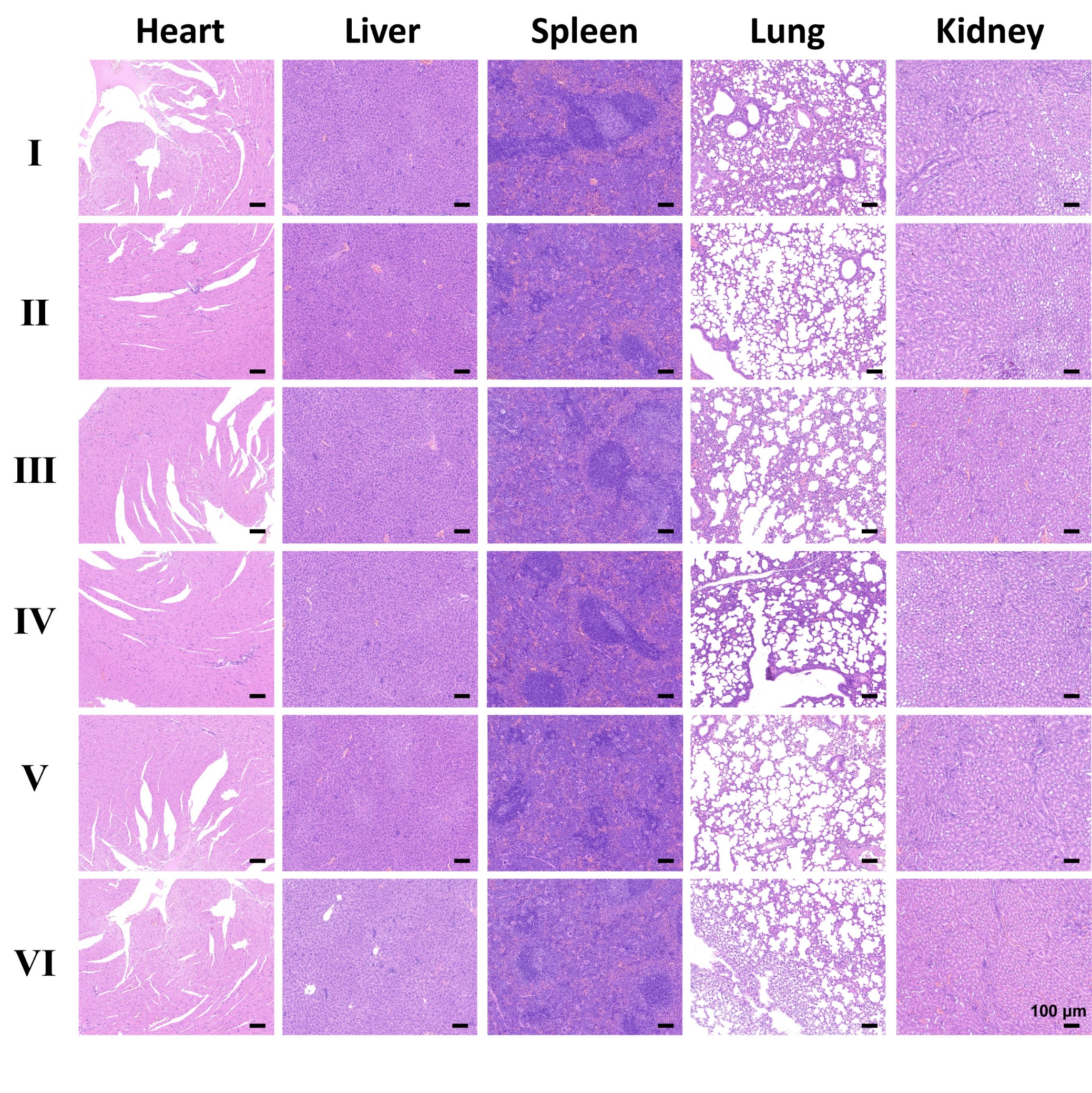


**Figure S49.** H&E staining images of major organs in SKOV3 tumor mice (I: PBS, II: PBT, III: PBT/Pt, IV: PBT/NO, Ⅴ: PBT + 1064 nm Laser, Ⅵ: PBT/NO/Pt + 1064 nm Laser, 1.0 W cm^-2^). (Scale bar: 100 μm).


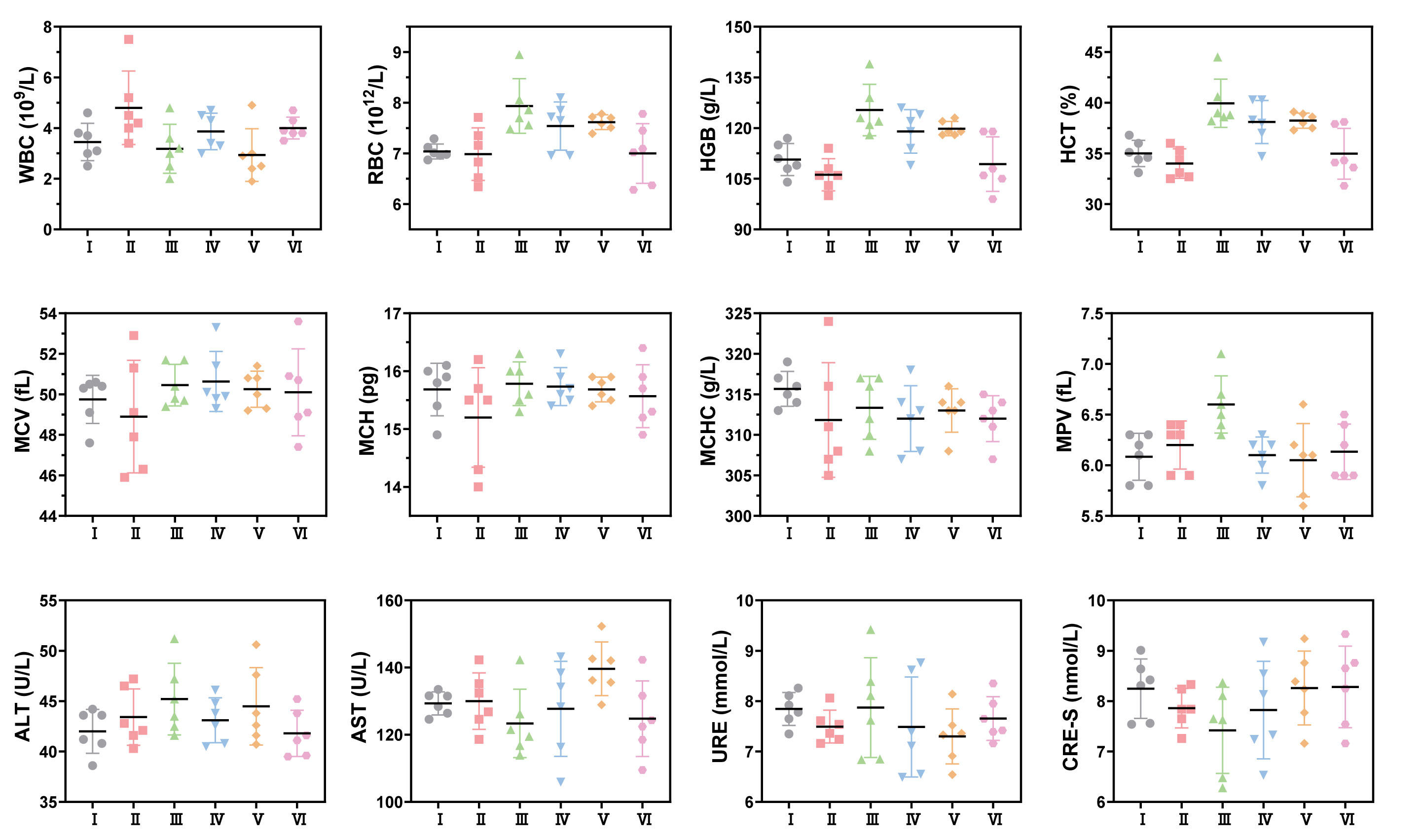


**Figure S50.** Blood analysis of the mice (I: PBS, II: PBT, III: PBT/Pt, IV: PBT/NO, Ⅴ: PBT + 1064 nm Laser, Ⅵ: PBT/NO/Pt + 1064 nm Laser, 1.0 W cm^-2^). Error bars, mean ± SD (n = 6).
